# Microbiome composition and metabolic pathways in shallow and deep periodontal pockets

**DOI:** 10.1038/s41598-025-97531-0

**Published:** 2025-04-15

**Authors:** Jale Moradi, Ellen Berggreen, Dagmar F. Bunæs, Anne Isine Bolstad, Randi Jacobsen Bertelsen

**Affiliations:** 1 Oral Health Center of Expertise in Western Norway, Bergen, Norway; 2 Department of Biomedicine, University of Bergen, Bergen, Norway; 3 Department of Clinical Dentistry, University of Bergen, Bergen, Norway; 4 Department of Clinical Science, University of Bergen, Bergen, Norway

**Keywords:** Periodontal pockets, Subgingival microbiome, Dysbiosis, Aging adults, Microbiology, Diseases, Medical research, Molecular medicine, Risk factors

## Abstract

In periodontal diseases, a dysbiotic subgingival microbiome interacts complexly with the host immune response and is strongly considered a risk factor for various systemic conditions. The high prevalence of both periodontal and systemic diseases in older adults highlights the importance of characterizing the subgingival microbiome in this age group. This study specifically characterizes the composition of the subgingival microbiome and investigates the interactions between microbial niches in shallow and deep periodontal pockets in individuals in their early 70s. We collected 1928 samples from 1287 participants, all born between 1950 and 1951. Participants had either shallow (≤ 4 mm) periodontal pockets or both shallow and deep (≥ 5 mm) periodontal pockets. Distinct microbial patterns were observed in shallow and deep periodontal pockets within the same oral cavity. Deep pockets exhibited a significantly higher abundance of species from genera such as *Prevotella*, *Centipeda*, *Treponema*, and *Fusobacterium*, while shallow pockets were enriched with species from *Actinomyces*, *Pauljensenia*, *Streptococcus*, and *Gemella*. The top significant species associated with deep pockets included *Fretibacterium fastidiosum*, *Tannerella forsythia*, and *Treponema denticola*, whereas shallow pockets were predominantly associated with *Actinomyces* species and *Rothia dentocariosa*. Additionally, shallow pockets in individuals with both pocket types showed a positive association with *Tannerella forsythia*, *Porphyromonas gingivalis*, and *Fusobacterium nucleatum* compared to shallow pockets in individuals with only shallow pockets. Metabolic pathways showed significant variation with pocket depth, with pathways such as lipopolysaccharide metabolism, lipid metabolism, and polyamine biosynthesis being positively associated with deep pockets. Overall, this study provides comprehensive microbiome analyses of periodontal pockets in aging adults, contributing to a better understanding of periodontal health and its potential impact on reducing systemic health risks in aging populations.

## Introduction

The human oral cavity hosts the second most abundant microbiome following the gastrointestinal tract^1^. Research on the oral microbiome has revealed a wide array of more than 700 bacterial species, primarily sourced from several dozen genera spanning seven phyla: *Actinomycetota, Bacteroidota, Bacillota, Fusobacteriota, Pseudomonadota, Saccharibacteria (TM7), and Spirochaetota*^2^. These diverse microbial communities play a critical role in preserving oral health, with profound implications for systemic health^3^.

Periodontitis is a prevalent condition among aging adults, affecting a significant portion of the population over the age of 65–74^4^. This chronic inflammatory disease leads to tooth loss and is linked to systemic conditions such as cardiovascular disease, diabetes, and respiratory infections^5^. Its progression in aging adults can exacerbate these conditions, highlighting the need to understand microbial dynamics^6^. Investigating the subgingival microbiome can reveal how microbial shifts drive local and systemic inflammation^7^. Furthermore, recent studies have linked microbial metabolites to disease development, highlighting the subgingival microbiome’s role in periodontal health^8^. Changes in the oral microenvironment can alter microbial metabolism, increasing pathogenicity and contributing to oral diseases^9^. Understanding these interactions may guide new diagnostic and therapeutic strategies for both periodontal and systemic diseases^10^.

Different niches in the oral cavity, including shallow and deep periodontal pockets, create distinct microbial environments influenced by host factors and disease progression^11^. Profiling the microbiome in these pockets is crucial for understanding periodontal health, disease mechanisms, and potential systemic implications^12^.

High-throughput sequencing has transformed our understanding of the oral microbiome, enabling the identification of diverse microorganisms and their functional roles^13^. Beyond known bacterial complexes like the ‘red’ and ‘orange’ complexes^14^, it has uncovered emerging pathogens such as *Filifactor alocis* and *Fretibacterium fastidiosum*, expanding insights into periodontal microbial ecology^15,16^.

In this study, we investigate the microbial dynamics within shallow and deep periodontal pockets using shotgun metagenomics. Specifically, we compare microbial composition and metabolic activities between these pockets to understand how pocket depth influences microbiome diversity and function. Additionally, we assess how deep pockets influence the microbial composition of shallow pockets within the same oral environment. This analysis provides insights into microbial shifts associated with periodontal disease progression in aging adults.

## Results

### Richness and diversity

**Fig. 1:**
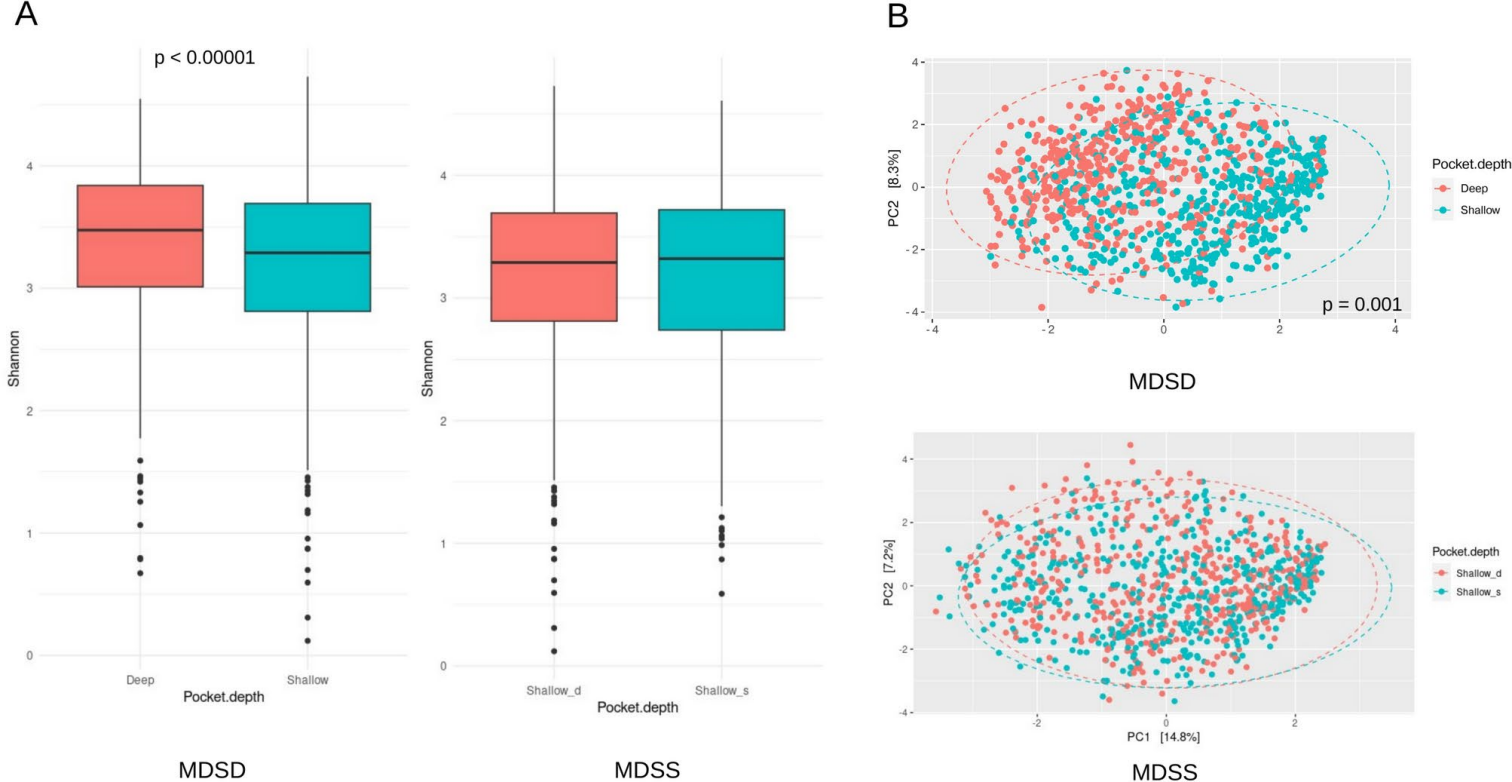
Richness and diversity of microbiome samples from deep and shallow periodontal pockets. (**A**) Alpha diversity measured using Shannon indices. (**B**) Beta diversity assessed using Aitchison distances. MDSD represents microbiome diversity comparison between deep and shallow pockets within the same oral cavity, while MDSS compares microbiome diversity between shallow pockets from individuals with both shallow and deep pockets (Shallow_d) and those with only shallow pockets (Shallow_s).

The oral microbiome was analyzed based on periodontal pocket depth. A significantly higher species richness was observed in deep pockets compared to shallow pockets within individuals, indicating a strong correlation between pocket depth and microbial diversity (p < 0.00001; Fig. 1A). This was further supported by beta diversity analysis, which revealed distinct microbial communities in deep versus shallow pockets within the same individuals (p = 0.001; Fig. 1B).

No significant differences in alpha and beta diversity were detected between shallow pockets in individuals with both shallow and deep pockets (Shallow_d) and those with only shallow pockets (Shallow_s). Although alpha diversity analysis suggested numerical differences in microbial richness and evenness, these differences were not statistically significant (Fig. 1A). Similarly, beta diversity analysis, which examines the variation in microbial community composition, showed no significant dissimilarities between Shallow_d and Shallow_s (Fig. 1B). However, subsequent analyses did reveal specific microbial species with significant differences, indicating nuanced variations in microbial communities not captured by overall diversity metrics.

### Microbiome composition in shallow and deep periodontal pocket types

**Fig. 2:**
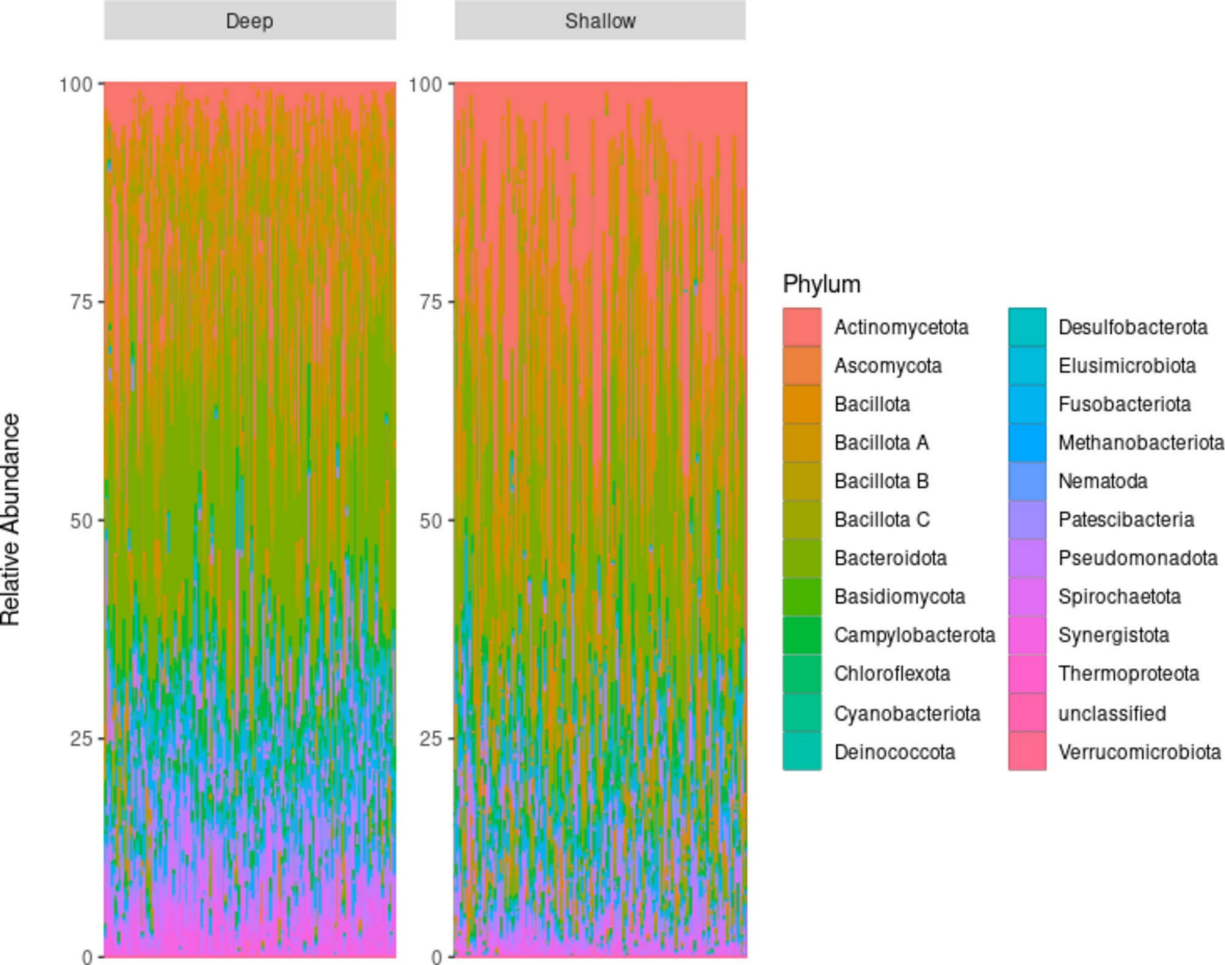
Taxonomic analysis of microbiome composition in periodontal pockets. The bar plot illustrates the relative abundance of taxa at the phylum level, comparing microbiome composition in deep and shallow pockets within the same oral cavity. *Actinomycetota*, *Bacteroidota*, and *Spirochaetota* were the dominant phyla in both deep and shallow pockets.

This comparative analysis of microbiome composition focused on deep and shallow periodontal pockets within the same oral environment. We first assessed microbial abundance at the phylum level, where boxplot analysis of pocket depth revealed distinct abundance patterns across various taxa (Fig. 2).

In both periodontal pockets, *Actinomycetota*, *Bacteroidota*, and *Spirochaetota* had significantly higher median abundances than other phyla. *Pseudomonadota*, *Fusobacteriota*, *Patescibacteria*, *Bacillota*, *Synergistota*, and *Campylobacterota* showed medium to low median abundances. In contrast, several taxa exhibited minimal or negligible abundances in both shallow and deep pockets. These included bacterial phyla (*Chloroflexota*, *Cyanobacteriota*, *Deinococcota*, *Elusimicrobiota*, *Verrucomicrobiota*), fungal taxa (*Ascomycota*, *Basidiomycota*), archaeal groups (*Methanobacteriota*, *Thermoproteota*), and parasitic taxa (*Nematoda*), along with some unclassified taxa (Supplementary Fig. 1).

**Fig. 3:**
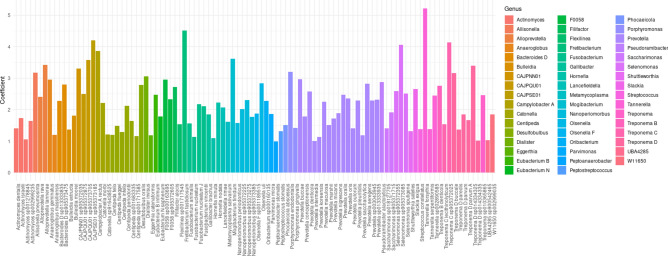
Top significant taxa associated with deep and shallow periodontal pockets in the same oral cavity. The scatter plot displays the top 100 species significantly associated with periodontal pocket depth. Each bar represents a species, positioned according to its coefficient and significance level. A positive coefficient indicates a species positively associated with deep pockets, while a negative coefficient indicates an association with shallow pockets. The analysis was performed using MaAsLin2. Notably, all top 100 significant species have positive coefficients, indicating a strong association with deep pockets.

Significant taxa associated with pocket type at the genus and species levels were identified using the MaAsLin2 multivariate linear model. The analysis initially focused on the relationship between pocket type and the relative abundance of bacterial taxa (Supplementary Table 1). A total of 85 significant genera and 233 species were identified, with *Prevotella* (25 species) being the most prevalent, followed by *Actinomyces* (18 species), *Centipeda* (10 species), and *Pauljensenia* (8 species). These genera, along with “*Saccharimonas”* and *Fusobacterium*, were among the most prevalent (Fig. 3).

Of the 233 significant species identified, 161 species (belonging to 66 distinct genera) were positively associated with deep pockets, while 72 species (from 30 distinct genera) were negatively associated with deep pockets, being positively correlated with shallow pockets (Supplementary Figs. 2, 3). The most prevalent genus in deep pockets was *Prevotella* (25 species), followed by *Centipeda* (9 species), *Treponema D* (6 species), and *Fusobacterium* (6 species). Among the top 20 significant species positively related to deep pockets were *Fretibacterium fastidiosum*, *Tannerella forsythia*, *Treponema denticola*, *Campylobacter rectus*, *Fusobacterium nucleatum*, *Mogibacterium timidum*, and *Dialister invisus*. In contrast, the most diverse genera in shallow pockets were *Actinomyces* (14 species), *Pauljensenia* (8 species), *Streptococcus* (5 species), and *Gemella* (4 species). The most significant species positively associated with shallow pockets were primarily *Actinomyces* species, including *Actinomyces oris* and *Actinomyces naeslundii*, as well as *Rothia dentocariosa*, *Lautropia mirabilis*, and *Streptococcus intermedius*. While many genera and species were unique to each pocket type, eleven genera—*Actinomyces*, *Streptococcus*, *Capnocytophaga*, “*Saccharimonas*”, *Leptotrichia*, *Campylobacter*, *Parvimonas*, *Centipeda*, *Fusobacterium*, and *Lancefieldella*—were significant in both pocket types.

The analysis identified 55 genera exclusively associated with deep pockets, indicating a stronger presence in deep pockets compared to shallow pockets. Conversely, nineteen genera were exclusively associated with shallow pockets, indicating a positive correlation with shallow pockets.

Regarding the positive associations with deep pockets, species of *Prevotella* (*P. buccae*, *P. intermedia*, *P. veroralis*, *P. marshii*, *P. oris*, *Prevotella* sp003043945, *Prevotella* sp013333935, *P. seregens*, *P. conceptionensis*, *Prevotella* sp018127805, and *Prevotella* sp000467895) were highly enriched in deep pockets. Similarly, *Centipeda* (*C. flueggei*, *C. infelix*, *C. felix*, *C. periodontii*, *C. timonae*, *Centipeda* sp001717585, *Centipeda* sp001683335, and *Centipeda* sp905372865), *Treponema* (*T. denticola*, *T. lecithinolyticum*, *Treponema* sp010365865, *Treponema* sp905373565, *Treponema* sp905372025), *Treponema_D* (*Treponema_D* sp014334325, *Treponema_D* paredis, *Treponema_D* parvum, *Treponema_D* buccale, *Treponema_D* sp014334335), and *Fusobacterium* (*F. animalis*, *F. nucleatum* D, *F. vincentii*, *F. nucleatum*) were significantly associated with deep pockets. All associations mentioned were statistically significant (*p* < 0.0001; Supplementary Table 1).

Regarding the negative associations with deep pockets, several *Actinomyces* species (*A. oris*, *A. johnsonii*, *A. naeslundii*, *A. massiliensis*, *Actinomyces* sp000195595, *Actinomyces* sp900323545, *Actinomyces* sp915069725) were significantly enriched in shallow pockets. Among *Pauljensenia*, *P. hongkongensis*, *P. pyogenes_A*, *Pauljensenia* sp000185285, and *P. odontolytica_A* were also more abundant in shallow pockets (all *p* < 0.01; Supplementary Table 1). Additionally, *Streptococcus* (*S. intermedius*, *S. gordonii*, and *S. sanguinis*) showed negative associations with deep pockets and positive associations with shallow pockets (all *p* < 0.0001; Supplementary Table 1).

### Microbial composition in shallow periodontal pockets

Alpha and beta diversity analyses did not reveal significant differences between *Shallow_d* and *Shallow_s*. However, MaAsLin2 analysis identified significant differences in their microbial composition.

**Fig. 4:**
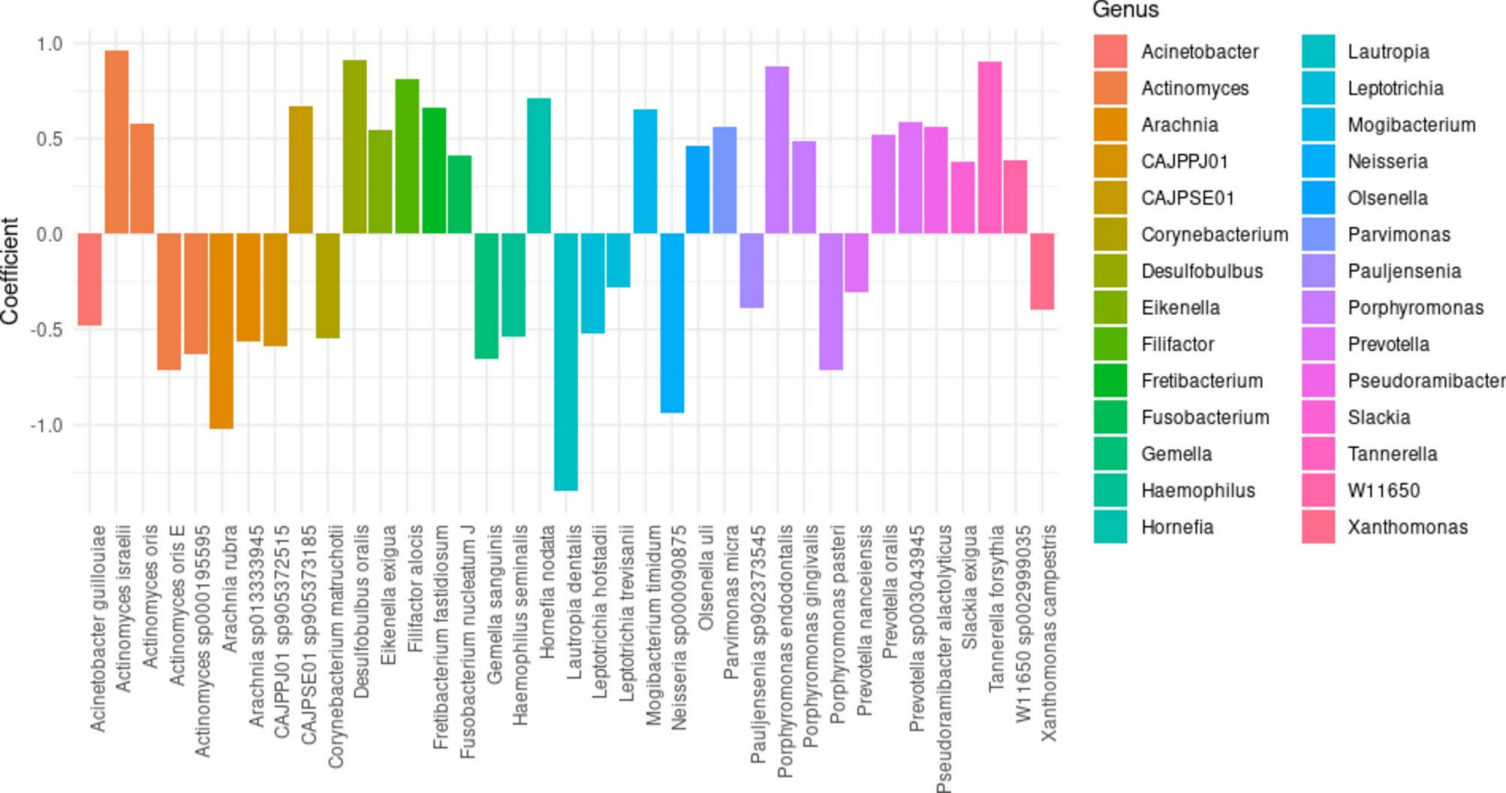
Significant taxa associated with shallow periodontal pockets in different oral cavity contexts. The scatter plot shows the significant species associated with shallow pockets in individuals with both deep and shallow pockets (*Shallow_d*) and those with only shallow pockets (*Shallow_s*). Positive coefficients represent species positively associated with *Shallow_d*, while negative coefficients indicate species positively associated with *Shallow_s*. The analysis was conducted using MaAsLin2.

20 species from 17 genera showed a positive association with *Shallow_d*. In contrast, 17 species from 14 genera were negatively associated with *Shallow_d*, indicating a preference for *Shallow_s* (Fig. 4). Among the 20 species positively associated with *Shallow_d*, *T. forsythia*, *Porphyromonas gingivalis*, *F. nucleatum*, *F. fastidiosum*, and *F. alocis* were notably significant. Conversely, among the 17 species negatively associated with *Shallow_d*, *Gemella sanguinis*, *Actinomyces oris*, and *Lautropia dentalis* were particularly significant.

### Microbial functional composition in shallow and deep periodontal pocket types

The analysis revealed associations between pocket depth and microbial metabolic pathways within the periodontal microbiome (Supplementary Table 3). A total of 125 significant pathways across 22 categories were associated with pocket depth. Carbohydrate metabolism was the predominant category, comprising 14 pathways related to central carbohydrate metabolism and 13 in other carbohydrate metabolism subcategories.

**Fig. 5:**
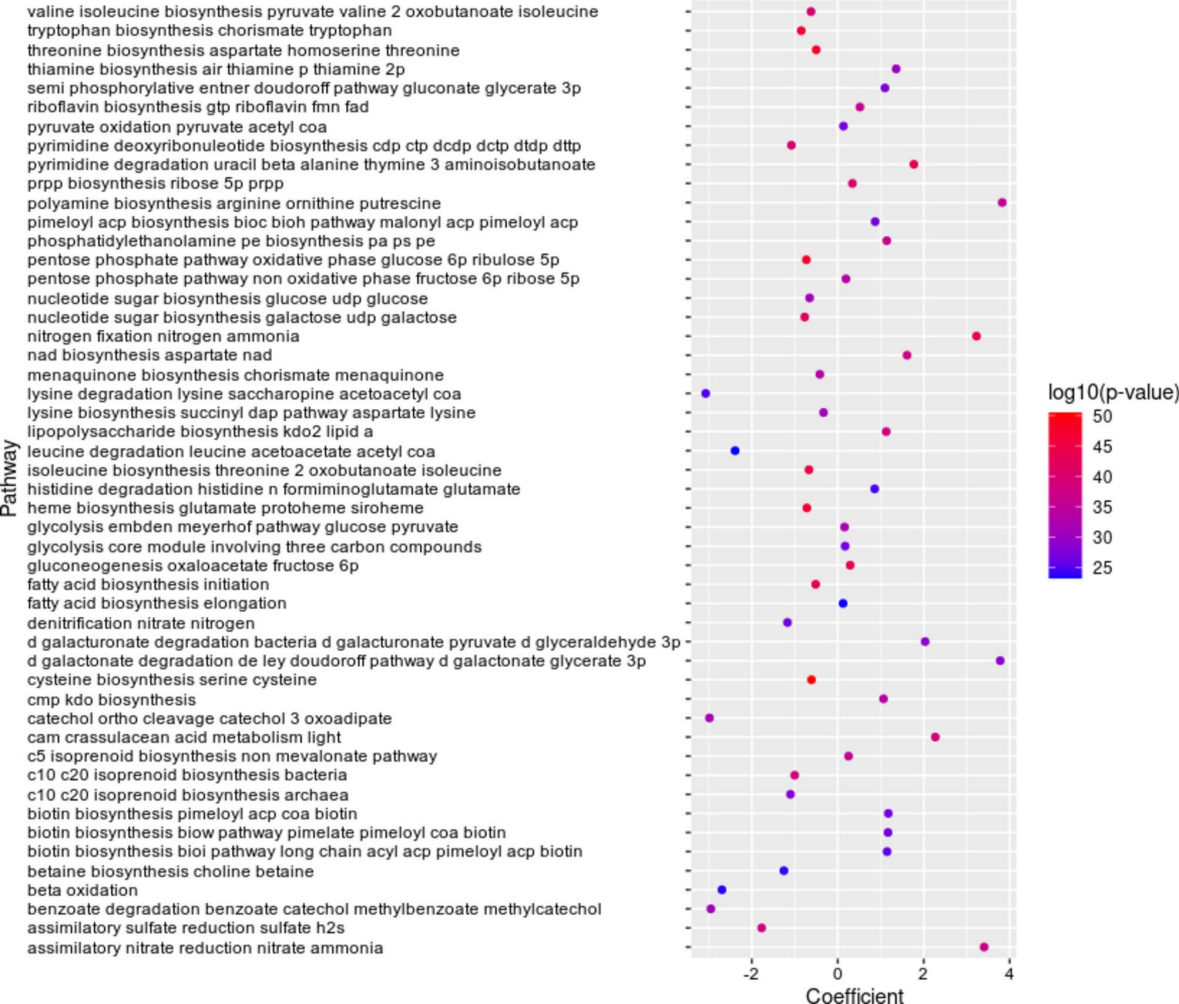
Significant microbial pathways correlated with the depth of periodontal pockets. The scatter plot shows the top 50 microbial pathways associated with periodontal pocket depth in individuals with both deep and shallow pockets. A positive coefficient indicates pathways positively associated with deep pockets, while a negative coefficient indicates pathways positively associated with shallow pockets. The analysis was conducted using MaAsLin2.

Several carbohydrate metabolism pathways correlated positively with pocket depth, including gluconeogenesis (oxaloacetate to fructose-6-phosphate, coef = 0.289) and trehalose biosynthesis (D-glucose-1-phosphate to trehalose, coef = 0.307). Conversely, pathways such as the pentose phosphate pathway (oxidative phase: glucose-6-phosphate to ribulose-5-phosphate, coef = −0.731), glycolysis (Embden-Meyerhof pathway: glucose to pyruvate, coef = −0.158), and pyruvate oxidation (pyruvate to acetyl-CoA, coef = −0.134; Fig. 5) exhibited negative correlations.

The cofactor and vitamin biosynthesis category was notably represented, with 18 pathways showing either positive or negative correlations with pocket depth. Positive correlations were observed for NAD biosynthesis (coef = 1.613), riboflavin biosynthesis (coef = 0.516), thiamine biosynthesis (coef = 1.358), biotin biosynthesis (coef = 1.176), and pyridoxal biosynthesis (coef = 2.146). In contrast, heme biosynthesis (coef = −0.719) and menaquinone biosynthesis (coef = −0.417) correlated negatively with deep pockets.

Amino acid metabolism pathways were also prominent. Positively correlated pathways included nitrogen fixation (nitrogen to ammonia, coef = 3.228), pyrimidine degradation (uracil to beta-alanine; thymine to 3-aminoisobutanoate, coef = 1.771), and polyamine biosynthesis (arginine to ornithine to putrescine, coef = 3.828). Pathways negatively associated with deep pockets included cysteine biosynthesis (serine to cysteine, coef = −0.611), threonine biosynthesis (aspartate to homoserine to threonine, coef = −0.500), and tryptophan biosynthesis (chorismate to tryptophan, coef = −0.850).

Additionally, some pathway categories were exclusively positively correlated with pocket depth, including lipopolysaccharide metabolism (coef = 1.012, *p* < 0.001), lipid metabolism (coef = 1.082, *p* < 0.001), polyamine biosynthesis (coef = 0.792, *p* < 0.001), pyrimidine metabolism (coef = 0.155, *p* < 0.001), and carbon fixation (coef = 0.133, *p* < 0.001; Fig. 5).

## Discussion

This study provides a comparative analysis of microbiome composition in different periodontal pocket types by examining both deep and shallow pockets within the same oral cavity. It explores the relationship between pocket depth and microbial diversity in subgingival environments.

Investigating the subgingival microbiome is essential not only for understanding local periodontal disease but also for its implications in systemic diseases through mechanisms such as transient bacteremia, microbial toxin circulation, and systemic inflammation^17^. This study explores whether the presence of deep pockets affects the microbial composition of shallow pockets by analyzing shallow_d (shallow pockets in individuals with both deep and shallow pockets) and shallow_s (shallow pockets in individuals with only shallow pockets). This approach provides insights into how different periodontal niches influence each other, which is essential for understanding disease progression and early microbial shifts^18^.

This study focuses on individuals in their 70s, providing a relevant cohort for investigating age-related changes in the oral microbiome and their potential systemic implications. Analyzing microbial diversity and composition in relation to periodontal pocket depth may reveal patterns associated with chronic conditions such as cardiovascular disease and diabetes^19^. Understanding these microbial shifts could inform targeted strategies for periodontal health management in aging populations and contribute to mitigating systemic health risks linked to oral microbiome alterations^7^.

In this study, *Actinomycetota*, *Bacteroidota*, and *Spirochaetota* exhibited higher median abundances in both deep and shallow pockets, indicating the dominance of disease-associated bacteria characteristic of periodontal disease^20^. The relatively lower abundance of health-associated phyla such as *Bacillota*, which are typically prevalent in healthy oral environments, suggests a microbial imbalance or dysbiosis even in shallow pockets^2,21^.

Additionally, several phyla with low abundance, including *Fusobactriota*, *Campylobacterota*, *Pseudomonadota*, and *Synergistota*, have previously been implicated in periodontal diseases^22–24^. The negligible detection of fungal taxa, such as *Ascomycota* and *Basidiomycota*, is consistent with prior studies emphasizing the minor role of fungi in the oral microbiome compared to bacteria^25,26^. While fungi are present in the oral cavity, their overall contribution to microbial composition and function appears to be limited.

Furthermore, the detection of certain taxa in low abundance, including environmentally associated bacterial groups (*Patescibacteria*, *Elusimicrobiota*, *Chloroflexota*, and *Verrucomicrobiota*), archaeal lineages (*Methanobacteriota* and *Thermoproteota*), and parasitic taxa (*Nematoda*), suggests potential environmental influences on microbial colonization in the oral cavity^27–29^.

The top significant species positively correlated with deep pockets were *F. fastidiosum*, *T. forsythia*, *T. denticola*, *C. rectus*, *F. nucleatum*, *M. timidum*, and *D. invisus*. These well-documented periodontal pathogens are known to induce inflammation and tissue destruction through potent virulence factors, such as adhesion and invasion of epithelial cells, production of proteolytic enzymes, and immune evasion strategies, which significantly contribute to the progression of periodontitis^30–32^. The presence of *C. rectus* and *F. nucleatum* further underscores the complexity of the biofilm in deep pockets, as these species are known for their synergistic interactions with other pathogens, enhancing biofilm stability and pathogenicity^33^. *M. timidum* and *D. invisus* also contribute to the pathogenic environment through their metabolic activities that support the growth of other anaerobes and their roles in modulating the host immune response^34,35^.

Several genera emerged as prevalent in deep pockets, with *Prevotella* species, particularly *P. intermedia* and *P. oris*, standing out. These findings are consistent with previous research linking *Prevotella* species to periodontal diseases, owing to their ability to thrive in anaerobic environments ^36,37^. Although *C. periodontii* has been associated with periodontitis and is significantly linked to deep pockets, its role in disease progression or adaptation to anaerobic conditions within periodontal pockets requires further exploration^38^. Additionally, other *Centipeda* species identified in this study, such as *Centipeda* sp001717585, *C. flueggei*, and *C. infelix*, have received limited attention in relation to periodontal diseases, indicating a need for further investigation into their potential contributions to disease progression.

Significant associations were observed with several *Treponema* species, including *T. denticola*, *T. parvum*, and *T. lecithinolyticum*, which have long been implicated in periodontal diseases. These findings reaffirm their roles as key pathogens in periodontitis^39–41^. Furthermore, associations with newly identified *Treponema* species highlight the necessity for further research into their specific contributions to both periodontal health and disease progression.

Among the *Fusobacterium* species, significant associations with deep pockets were identified, particularly for *F. animalis* and *F. vincentii*. These results highlight the continued importance of *Fusobacterium* in the pathogenesis of periodontitis, reinforcing its role as one of the major contributor to periodontal disease^42,43^.

In contrast, the top significant species positively associated with shallow pockets were predominantly *Actinomyces* species, including *A. oris* and *A. naeslundii*, along with *R. dentocariosa*, *L. mirabilis*, and *S. intermedius*. These species generally support gingival health by producing protective extracellular polysaccharides, maintaining microbial homeostasis, and inhibiting pathogen overgrowth^44^. Although some of these species can act as opportunistic pathogens under specific conditions, their presence in shallow pockets suggests a more balanced microbial profile compared to the dysbiotic environment typically found in deep pockets.

The genera that were significantly positively associated with shallow pockets indicate a preference for shallower environments, with reduced presence in deeper pockets. *Actinomyces* species were significantly associated with shallow pockets, suggesting their potential role in maintaining periodontal health or in the early stages of disease. These findings align with the commensal nature of *Actinomyces* in the oral cavity, where they contribute to biofilm formation and dental plaque development^45,46^. Similarly, *Pauljensenia* species exhibited significant associations with shallow pockets. While less studied than *Actinomyces*, *Pauljensenia* species have been implicated in the composition of the oral microbiome^47^. Additionally, *Streptococcus* species were significantly negatively associated with deep pockets^48^. These results emphasize the distinct microbial ecosystems in deep and shallow periodontal pockets. The existence of more significant commensal species in shallow pockets plays a protective role in maintaining oral health, whereas pathogenic species in deep pockets contribute to disease progression through mechanisms that enhance their survival and pathogenicity in anaerobic conditions.

The analysis of microbial associations with shallow periodontal pockets in shallow_d and shallow_s revealed distinct patterns. While alpha and beta diversity analyses did not show significant differences between the shallow_d and shallow_s pockets, MaAsLin2 analysis identified notable differences in specific microbial species. This suggests that, although overall microbial diversity and community structure may remain similar, certain microbial species display differential profiles in these distinct microenvironments.

Notably, *P. gingivalis*, *T. forsythia*, and *F. fastidiosum* were positively associated with shallow pockets in individuals with both pocket types, consistent with their well-established roles as key pathogens in periodontitis^49–51^. Additionally, *A. israelii*, *D. oralis*, and *H. nodata* were uniquely associated with shallow pockets in individuals with both pocket types. *A. israelii*, a commensal of periodontal pockets, has been linked to periodontitis patients^52^, while *Hornefia*, primarily studied in the context of neonatal enteric infections, exhibited a positive association with periodontal pockets in this study^53^.

Conversely, *G. sanguinis* and *Neisseria* species showed negative associations with shallow pockets in individuals with both pocket types. These species have been previously linked to periodontal health and are rarely reported as indicators of periodontal disease^54,55^. Other taxa negatively associated with shallow pockets in the presence of deep pockets—including *Arachnia*, *Xanthomonas*, *Haemophilus*, *Corynebacterium*, *Pauljensenia*, *Leptotrichia*, and *Acinetobacter*—are primarily components of the oral microbiome, though their specific roles in disease progression remain unclear.

These findings suggest that the microbial environment of deep pockets may influence that of shallow pockets, reflecting complex dynamics within the oral microbiome. Understanding these interactions is essential for developing targeted therapeutic strategies for periodontal health management. The taxa associated with shallow pockets in individuals with both pocket types indicate a potential shift toward a disease-associated profile compared to those with only shallow pockets.

The metabolic pathways of the periodontal microbiome play a key role in shaping the dysbiotic microbiota associated with gingivitis and periodontitis. These inflammatory conditions arise and persist due to polymicrobial dental plaque/biofilm, which thrives in the sheltered environment of the periodontal pocket, protected from natural cleansing mechanisms like saliva. The paradoxical nature of the heightened inflammatory response facilitates the colonization of slow-growing anaerobic bacteria—primarily Gram-negative species with proteolytic metabolism—thereby driving disease progression^56,57^.

In this study, deep pockets showed significant positive correlations with several microbial pathway categories, including lipopolysaccharide metabolism, lipid metabolism, polyamine biosynthesis, and pyrimidine metabolism. Lipopolysaccharides from periodontal bacteria act as potent immunostimulatory molecules, driving inflammation and tissue destruction within the periodontium^58^. The observed associations between these pathways and deep pockets reinforce the concept that microbial dysbiosis in periodontal pockets contributes to increased virulence and tissue damage.

The significant elevation of metabolic pathways, including lipid metabolism, polyamine biosynthesis, pyrimidine metabolism, and carbon fixation, in deep periodontal pockets compared to shallow pockets underscores their potential as indicators of periodontal disease. Additionally, lipid-derived metabolites produced by periodontal bacteria may contribute to dysbiosis, influencing the composition and function of the subgingival microbiota^59^.

Similarly, polyamine biosynthesis pathways, which produce bioactive molecules such as putrescine and spermine, have been found in higher concentrations at periodontitis-affected sites and decrease following treatment^56^. Additionally, elevated pyrimidine metabolism, essential for nucleotide synthesis and bacterial proliferation, may indicate increased microbial turnover within deep pockets, further contributing to disease progression^60^.

In summary, this study provides a comprehensive analysis of the microbial composition and metabolic pathways in shallow and deep periodontal pockets among individuals in their 70s, emphasizing the role of the oral microbiome in aging populations. We identified distinct microbial profiles and metabolic activities associated with pocket depth, with deep pockets exhibiting a unique microbial composition and heightened metabolic activity compared to shallow pockets. Furthermore, the presence of deep pockets appeared to influence the microbial environment of shallow pockets. Given the high prevalence of periodontitis in older adults and its associations with systemic diseases such as cardiovascular disease and diabetes, our findings highlight the importance of studying the oral microbiome in this age group.

## Materials and methods

### Study population, clinical examination, sampling, and analysis strategy

This study is part of the HUSK-T (HUSK Dental Health) population-based study in Western Norway, which includes 1928 samples collected from 1287 participants born in 1950–1951^61^. These participants underwent comprehensive dental and periodontal examinations (Supplementary Table 4). Oral examinations were performed by a dentist and included X-rays (2BW and OPG), a mucosal examination, dental status recording, caries assessment, and periodontal measurements. Periodontal probing depth was measured at six sites per tooth to assess pocket depth and bleeding on probing (BOP).

Subgingival bacterial samples were collected from two periodontal pockets of distinct teeth, primarily from the upper jaw. Participants were instructed to fast for 1 h prior to sample collection. The sampling area was dried, and supragingival plaque was carefully removed using cotton rolls. Subgingival plaque samples were collected using sterile paper points (COLTENE, USA), which were inserted into each selected pocket and left in place for 20s. Each sample consisted of pooled material from two periodontal sites and was categorized into two groups based on pocket depth and bleeding on probing: shallow pockets (≤ 4 mm) without bleeding on probing and deep pockets (≥ 5 mm) with bleeding on probing. For participants with both shallow and deep pockets, one sample was collected from each type of pocket. For those with only shallow pockets, a single sample was collected.

All samples were immediately stored in FluidX tubes at −80 °C for preservation. Blank control samples were also collected from every 20 participants to monitor potential contamination.

To investigate microbiome variations within different periodontal niches, the analysis was divided into two parts. The first part focused on intra-individual microbiome differences between shallow and deep pockets. By examining these intra-individual differences, the study aimed to understand the dynamic interactions and influences between microbial communities in varying pocket depths.

The second part compared microbiome differences between shallow pockets in individuals with both shallow and deep pockets and those with only shallow pockets. This comparison aimed to determine if the presence of deep pockets in the oral environment influences the microbial composition of shallow pockets. By assessing these differences, the study sought to provide insights into the ecological relationships within the oral microbiome.

The HUSK-T registry (No. 279585) was approved by the Norwegian Centre for Research Data (NSD) and by the data protection officer in Western Norway County. This study was also approved by the Norwegian Regional Committee for Medical and Health Research Ethics (REK No. 263006). Written informed consent was obtained from all participants, and the study was conducted in accordance with the principles of the Declaration of Helsinki.

### Shotgun metagenomics sequencing, preprocessing and profiling

Samples (n = 1928) were shipped on dry ice for DNA extraction and shotgun sequencing at Clinical Microbiomics AS (Copenhagen, Denmark). DNA was extracted using the NucleoSpin Soil 96 kit, and sequencing was performed on an Illumina NovaSeq 6000 platform, generating 2 × 150 bp paired-end reads with an average of 33.6 million read pairs per sample.

Raw sequencing data underwent quality control to remove host contamination and low-quality reads. Host contamination was filtered by discarding read pairs in which either read mapped to the human genome (GRCh38) using Bowtie2 in local alignment mode. Adapter sequences and bases with a Phred score below 30 were trimmed using AdapterRemoval. Read pairs were retained if both reads passed filtering and had a length of at least 100 bp. These high-quality non-host (HQNH) reads were used for downstream analyses.

HQNH reads were mapped to the HMR05 gene catalog using BWA mem^62^. Reads were classified as uniquely mapped if they aligned to a single gene with ≥ 95% identity over ≥ 100 bp and had a mapping quality (MAPQ) score ≥ 20. Reads were classified as multi-mapped if they aligned to multiple genes but did not meet the criteria for unique mapping, and as unmapped if neither read aligned to any gene. Only uniquely mapped reads were used for species abundance profiling.

Species abundance was calculated based on signature genes using a negative binomial distribution model, accounting for effective gene length, mapping alignment criteria, and read dispersion. Abundances were normalized such that the total species abundance for each sample summed to 100%. Taxonomic classification was performed using the genome taxonomy database (GTDB).

Functional potential was assessed by mapping genes to orthologous groups using EggNOG-mapper and functional databases such as the KEGG Orthology (KO). Functional profiles were computed based on the proportion of mapped reads assigned to specific KO categories. Functional Gene Sets (FGS) were used to define KEGG modules, encompassing the necessary genes for module functionality.

### Richness, diversity, and abundance analyses

Species richness and diversity were assessed using the ‘estimate_richness’ function from the R package ‘phyloseq,’ incorporating observed species, Chao1, and Shannon indices. Alpha diversity variations across categorical variables were statistically evaluated using Wilcoxon rank-sum tests. Beta diversity was quantified using Aitchison distances, calculated as Euclidean distances on CLR-transformed species-level data. Redundancy analysis (RDA) was performed on the Aitchison distances to visualize community-level differences. PERMANOVA, using the adonis function from the ‘vegan’ package, was applied to test for significant differences in beta diversity across groups.

To analyze taxonomic composition at specific levels, agglomerated reads were processed using ‘phyloseq’ and relative abundance for each variable was visualized. Box plots, generated with the ‘phyloseq::melt’ function, displayed the distribution of relative abundance for each taxonomic level. The HMP (Human Microbiome Project) package was used to assess differences in abundance at specific taxonomic levels. This was achieved through a multivariate test aimed at discerning differences in the overall composition between groups of samples^63^.

### Statistical analysis of microbiome data associations with metadata variables

Statistical analyses were conducted to elucidate associations between taxonomic and functional microbial features and metadata variables using the MaAsLin2 package from the bioBakery suite in R/Bioconductor^64^. The investigation encompassed both taxonomic and functional profiling, providing a comprehensive exploration of microbial interactions with metadata. The outcomes were visually represented through scatter plots, offering a nuanced understanding of the relationships between microbial features and metadata variables.

In the MaAsLin2 analysis, a comprehensive strategy with default parameters was implemented, including normalization using the TSS method, log transformation, LM analysis method, and BH correction method. The significance threshold was set at a q-value < 0.25, and features (species and pathways) with a q-value < 0.15 (nominal p-value < 0.05) were selected for increased confidence. The minimum abundance for each feature was set at 0.001 (0.1%). Prevalence thresholds were set at 0.3 (30%) for bacteria and 0.1 (10%) for pathways.

## Supplementary Information

Supplementary Information.

## Data Availability

All data generated or analyzed in this study are included in this published article and its supplementary information files. The raw metagenomic sequencing data are publicly available in the European Nucleotide Archive (ENA) under the accession number PRJEB87865.
