# Supplementary material for: Microbiome composition and metabolic pathways in shallow and deep periodontal pockets
Source: Sci Rep. 2025 Apr 15;15:12926. doi: 10.1038/s41598-025-97531-0 (PMC12000285; doi:10.1038/s41598-025-97531-0)
Supplement: Supplementary file 1 — Supplementary Information. [file 41598_2025_97531_MOESM1_ESM.pdf]

Supplementary information

Supplementary Figure 1. Comparison of microbial relative abundance at the phylum level between deep and shallow pockets within the same oral cavity.

Supplementary Table 1. Significant taxa identified by MaAsLin2 analysis, indicating their association with deep and shallow pockets in the same oral cavity.

Supplementary Table 2. Significant taxa identified by MaAsLin2 analysis, indicating their association with shallow pockets within samples from individuals with only shallow pockets and samples from individuals with both shallow and deep pockets. "shallow_s" refers to shallow pockets in individuals with only shallow pockets, and "Shallow_d" refers to shallow pockets in individuals with both shallow and deep pockets.

Supplementary Table 3. Significant microbial pathways identified by MaAsLin2 analysis, indicating their association with deep and shallow pockets within the same oral cavity.

Supplementary Figure 2. Significant species associated with pocket depth. The scatter plot shows the significant species which positively associated with deep pocket (and negatively associated with shallow pockets).

Supplementary Figure 3. Significant species associated with pocket depth. The scatter plot shows the significant species which negatively associated with deep pocket (and positively associated with shallow pockets).

Supplementary Table 4. Metadata including demographic information and dental clinical examination findings. Periodontal parameters include probing pocket depth (PPD), gingival recession (GR), and bleeding on probing (BOP), measured at six sites per tooth (excluding third molars). Clinical attachment loss (CAL) was calculated as PPD + GR, except at sites with restorations or gingival enlargement. Note: metadata reflects the 1646 successfully sequenced samples used in the analysis. A total of 282 samples were excluded from sequencing due to insufficient DNA mass.

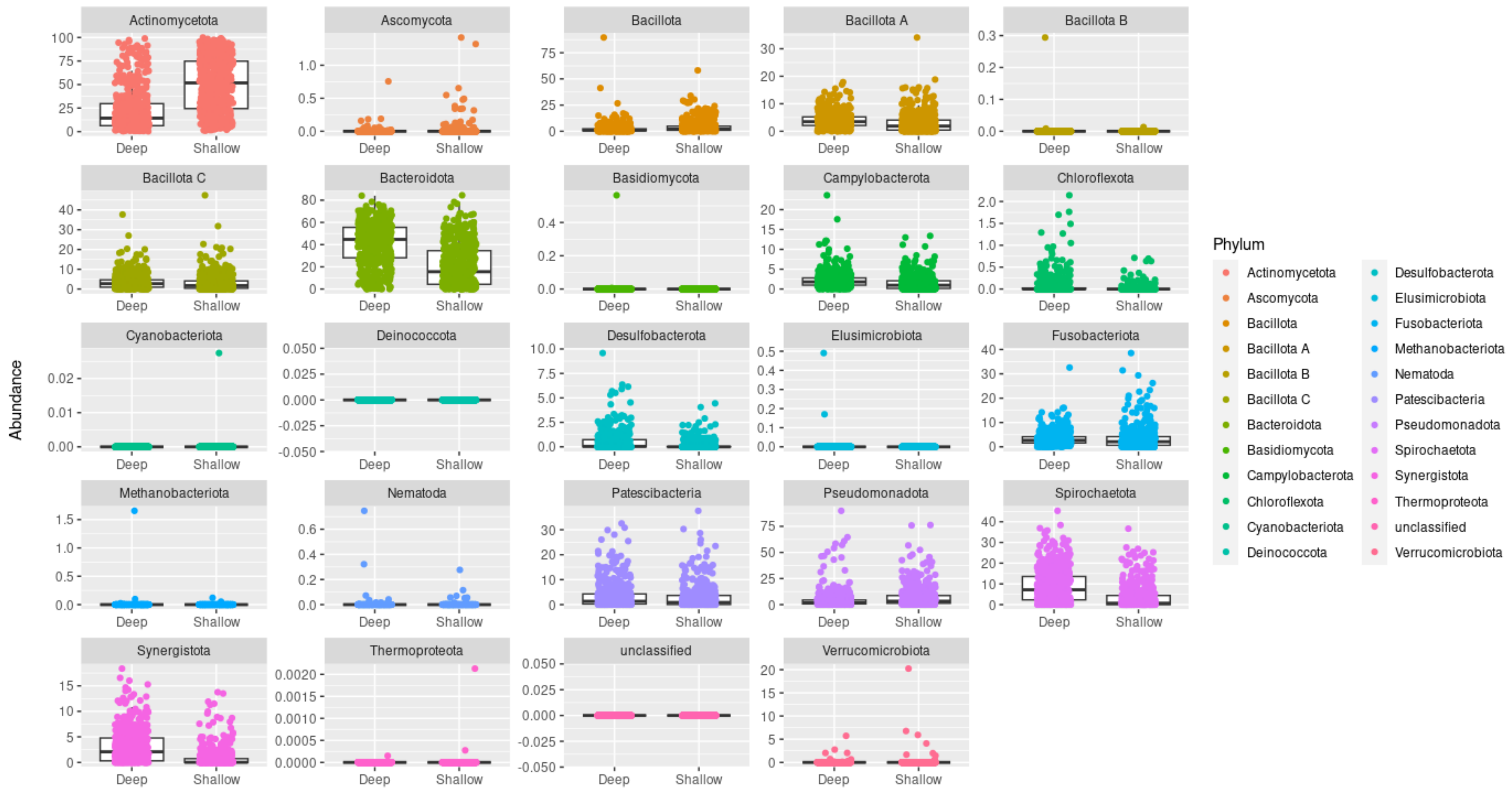

Supplementary Figure 1. Comparison of microbial relative abundance at the phylum level between deep and shallow periodontal pockets.

Supplementary Table 1: Significant taxa associated with shallow and deep periodontal pocket depths

superkingdom	phylum	order	family	genus	species	metadata	value	coef	stderr	N.not.0	pval	qval
Bacteria	Spirochaetota	Treponematales	Treponemataceae	Treponema D	Treponema D sp014334325	Pocket.depth	Deep	3.40228968277	0.202863665427	567	8.09E-56	2.7E-53
Bacteria	Synergistota	Synergistales	Aminobacteriaceae	Fretibacterium	Fretibacterium fastidiosum	Pocket.depth	Deep	4.51003896677	0.287680596448	591	1.08E-49	1.8E-47
Bacteria	Bacillota C	Selenomonadales	Selenomonadaceae	CAJPQU01	CAJPQU01 sp905373705	Pocket.depth	Deep	3.57624581166	0.237830126781	502	3.18E-46	3.5E-44
Bacteria	Bacteroidota	Bacteroidales	Tannerellaceae	Tannerella	Tannerella forsythia	Pocket.depth	Deep	5.22122490499	0.350907272999	770	2.2E-45	1.8E-43
Bacteria	Bacillota C	Selenomonadales	Selenomonadaceae	Selenomonas	Selenomonas sp905373085	Pocket.depth	Deep	4.06068670802	0.279004053045	494	1.14E-43	7.7E-42
Bacteria	Actinomycetota	Coriobacteriales	Atopobiaceae	Olsenella	Olsenella uli	Pocket.depth	Deep	2.83322487201	0.204954971792	398	6.67E-40	3.2E-38
Bacteria	Fusobacteriota	Fusobacteriales	Fusobacteriaceae	Fusobacterium	Fusobacterium nucleatum J	Pocket.depth	Deep	2.17461970751	0.160457653741	399	1.54E-38	5.7E-37
Bacteria	Spirochaetota	Treponematales	Treponemataceae	Treponema D	Treponema D buccale	Pocket.depth	Deep	3.16671251639	0.233869009694	721	1.77E-38	5.9E-37
Bacteria	Actinomycetota	Coriobacteriales	Eggerthellaceae	Slackia	Slackia exigua	Pocket.depth	Deep	2.65340427383	0.197472453815	516	5.83E-38	1.8E-36
Bacteria	Bacillota A	Peptostreptococcales	Anaerovoracaceae	CAJPNN01	CAJPNN01 sp905372035	Pocket.depth	Deep	3.30505416854	0.250979419156	504	1.23E-36	3.4E-35
Bacteria	Synergistota	Synergistales	Aminobacteriaceae	CAJPSE01	CAJPSE01 sp905373185	Pocket.depth	Deep	4.19654144481	0.318872865438	482	1.35E-36	3.5E-35
Bacteria	Bacillota A	Peptostreptococcales	Anaerovoracaceae	Mogibacterium	Mogibacterium timidum	Pocket.depth	Deep	3.6213220545	0.275708049424	539	1.8E-36	4.3E-35
Bacteria	Bacillota C	Veillonellales	Dialisteraceae	Allisonella	Allisonella pneumosinta	Pocket.depth	Deep	3.17844721815	0.253164956278	474	1.12E-33	2.5E-32
Bacteria	Bacteroidota	Bacteroidales	Bacteroidaceae	Prevotella	Prevotella buccae	Pocket.depth	Deep	2.96859901439	0.250435816818	381	2E-30	4.2E-29
Bacteria	Bacillota C	Veillonellales	Dialisteraceae	Dialister	Dialister invisus	Pocket.depth	Deep	3.05241702029	0.259140543543	646	4.39E-30	8.7E-29
Bacteria	Spirochaetota	Treponematales	Treponemataceae	Treponema C	Treponema C sp905372025	Pocket.depth	Deep	4.13128251162	0.355095375392	595	2E-29	3.7E-28
Bacteria	Bacillota C	Selenomonadales	Selenomonadaceae	Selenomonas	Selenomonas sputigena	Pocket.depth	Deep	2.50971516379	0.215949612798	458	2.24E-29	4E-28
Bacteria	Campylobacterota	Campylobacterales	Campylobacteraceae	Campylobacter A	Campylobacter A rectus	Pocket.depth	Deep	3.85970135987	0.337526687553	487	1.52E-28	2.5E-27
Bacteria	Actinomycetota	Actinomycetales	Actinomycetaceae	Actinomyces	Actinomyces oris	Pocket.depth	Deep	-2.78079974254	0.246442983516	712	7E-28	1.1E-26
Bacteria	Bacillota A	Eubacteriales	Eubacteriaceae	Pseudoramibacter	Pseudoramibacter alactolyticus	Pocket.depth	Deep	2.87671359935	0.257543187673	368	2.21E-27	3.4E-26
Bacteria	Spirochaetota	Treponematales	Treponemataceae	Treponema B	Treponema B denticola	Pocket.depth	Deep	2.7640417025	0.248097548962	429	3E-27	4.3E-26
Bacteria	Bacillota C	Veillonellales	Megasphaeraceae	Anaeroglobus	Anaeroglobus geminatus	Pocket.depth	Deep	2.94925570512	0.264857848776	404	3.13E-27	4.4E-26
Bacteria	Bacillota A	Peptostreptococcales	Anaerovoracaceae	Eubacterium B	Eubacterium B infirmum	Pocket.depth	Deep	2.46845668973	0.225849224948	371	2.4E-26	3.2E-25
Bacteria	Bacillota C	Selenomonadales	Selenomonadaceae	CAJPQU01	CAJPQU01 sp905372875	Pocket.depth	Deep	2.50422069784	0.236776210151	400	7.45E-25	9.6E-24
Bacteria	Bacteroidota	Bacteroidales	Paludibacteraceae	F0058	F0058 sp905372605	Pocket.depth	Deep	2.33515530119	0.226536365943	373	9.52E-24	1.1E-22
Bacteria	Spirochaetota	Treponematales	Treponemataceae	Treponema D	Treponema D parvum	Pocket.depth	Deep	1.84642595608	0.179092516186	234	9.36E-24	1.1E-22
Bacteria	Spirochaetota	Treponematales	Treponemataceae	Treponema D	Treponema D parvum A	Pocket.depth	Deep	1.66590618113	0.161692942001	231	1E-23	1.2E-22
Bacteria	Bacteroidota	Bacteroidales	Bacteroidaceae	Alloprevotella	Alloprevotella tannerae	Pocket.depth	Deep	3.41921822535	0.332450171274	609	1.18E-23	1.3E-22
Bacteria	Bacteroidota	Bacteroidales	Bacteroidaceae	Prevotella	Prevotella seregens	Pocket.depth	Deep	2.82395767312	0.276492765332	302	2.31E-23	2.5E-22
Bacteria	Bacteroidota	Bacteroidales	Tannerellaceae	Tannerella	Tannerella sp002890585	Pocket.depth	Deep	2.4422185466	0.241095259107	342	5E-23	5.3E-22
Bacteria	Actinomycetota	Actinomycetales	Micrococcaceae	Rothia	Rothia dentocariosa	Pocket.depth	Deep	-2.2962627476	0.231361785011	958	3.31E-22	3.4E-21
Bacteria	Bacteroidota	Bacteroidales	Bacteroidaceae	Alloprevotella	Alloprevotella rava	Pocket.depth	Deep	2.409768515	0.247442825206	346	1.79E-21	1.8E-20
Bacteria	Actinomycetota	Actinomycetales	Actinomycetaceae	Actinomyces	Actinomyces sp915069725	Pocket.depth	Deep	-2.78900394292	0.288273500605	666	3.17E-21	3E-20
Bacteria	Actinomycetota	Actinomycetales	Actinomycetaceae	Actinomyces	Actinomyces massiliensis	Pocket.depth	Deep	-2.61302511741	0.275537045198	783	1.73E-20	1.6E-19
Bacteria	Bacteroidota	Bacteroidales	Porphyromonadaceae	Porphyromonas	Porphyromonas endodontalis	Pocket.depth	Deep	3.20552973406	0.339571505626	561	2.54E-20	2.2E-19
Bacteria	Spirochaetota	Treponematales	Treponemataceae	Treponema D	Treponema D paredis	Pocket.depth	Deep	1.367899264	0.148158175591	494	1.53E-19	1.3E-18

Bacteria	Bacteroidota	Bacteroidales	Paludibacteraceae	F0058	F0058 sp000163695	Pocket.depth	Deep	2.95346171054	0.326799207648	759	8.11E-19	6.6E-18
Bacteria	Bacillota	Erysipelotrichales	Coprobacillaceae	Eggerthia	Eggerthia catenaformis	Pocket.depth	Deep	1.18476535623	0.132411238479	135	1.73E-18	1.3E-17
Bacteria	Bacteroidota	Bacteroidales	Bacteroidaceae	Prevotella	Prevotella sp003043945	Pocket.depth	Deep	2.28597548616	0.255470681832	284	1.72E-18	1.3E-17
Bacteria	Bacillota	Lactobacillales	Aerococcaceae	Granulicatella	Granulicatella adiacens	Pocket.depth	Deep	-2.1799312596	0.244906460974	677	2.55E-18	1.9E-17
Bacteria	Bacillota C	Selenomonadales	Selenomonadaceae	Selenomonas	Selenomonas sp905372355	Pocket.depth	Deep	2.59527048947	0.291880445751	588	2.76E-18	2E-17
Bacteria	Chloroflexota	Anaerolineales	Anaerolineaceae	Flexilinea	Flexilinea sp001717545	Pocket.depth	Deep	1.53472695512	0.176697597095	205	1.51E-17	1.1E-16
Bacteria	Bacteroidota	Bacteroidales	Bacteroidaceae	Prevotella	Prevotella sp013333935	Pocket.depth	Deep	2.31551835058	0.268086195208	586	2.25E-17	1.5E-16
Bacteria	Bacillota A	Lachnospirales	Lachnospiraceae	Catonella	Catonella morbi	Pocket.depth	Deep	2.21655524181	0.256802956887	642	2.36E-17	1.6E-16
Bacteria	Bacillota A	Peptostreptococcales	Filifactoraceae	Filifactor	Filifactor alocis	Pocket.depth	Deep	2.72475601822	0.316441812989	395	2.79E-17	1.8E-16
Bacteria	Pseudomonadota	Pseudomonadales	Moraxellaceae	Acinetobacter	Acinetobacter guillouiae	Pocket.depth	Deep	-1.72699202967	0.200810615231	184	3E-17	2E-16
Bacteria	Bacillota A	Peptostreptococcales	Anaerovoracaceae	Hornefia	Hornefia nodata	Pocket.depth	Deep	2.22343873931	0.259723869667	342	4.18E-17	2.6E-16
Bacteria	Bacillota A	Lachnospirales	Vallitaleaceae	W11650	W11650 sp002999035	Pocket.depth	Deep	1.84308638978	0.216296369497	248	5.75E-17	3.6E-16
Bacteria	Actinomycetota	Actinomycetales	Actinomycetaceae	Actinomyces	Actinomyces naeslundii	Pocket.depth	Deep	-2.30336376766	0.271627641451	778	8E-17	4.9E-16
Bacteria	Pseudomonadota	Burkholderiales	Neisseriaceae	Kingella B	Kingella B oralis	Pocket.depth	Deep	-2.04253421142	0.243899411506	709	1.86E-16	1.1E-15
Bacteria	Desulfobacterota	Desulfobulbales	Desulfobulbaceae	Desulfobulbus	Desulfobulbus oralis	Pocket.depth	Deep	2.77963863412	0.3346865672	391	3.21E-16	1.9E-15
Bacteria	Bacillota C	Selenomonadales	Selenomonadaceae	Centipeda	Centipeda periodontii	Pocket.depth	Deep	2.12137805729	0.255483014142	323	3.26E-16	1.9E-15
Bacteria	Bacillota A	Tissierellales	Peptoniphilaceae	Parvimonas	Parvimonas micra	Pocket.depth	Deep	1.85676916983	0.224657560848	726	4.41E-16	2.5E-15
Bacteria	Patescibacteria	Saccharimonadales	Saccharimonadaceae	Saccharimonas	Saccharimonas sp905371715	Pocket.depth	Deep	1.90986661641	0.23361331741	413	8.88E-16	4.9E-15
Bacteria	Patescibacteria	Saccharimonadales	Nanoperiomorbaceae	Nanoperiomorbus	Nanoperiomorbus sp905373385	Pocket.depth	Deep	1.76557787804	0.215909985146	326	8.74E-16	4.9E-15
Bacteria	Bacteroidota	Bacteroidales	Bacteroidaceae	Prevotella	Prevotella micans	Pocket.depth	Deep	1.72295628702	0.210830282099	253	9.09E-16	4.9E-15
Bacteria	Spirochaetota	Treponematales	Treponemataceae	Treponema	Treponema sp010365865	Pocket.depth	Deep	2.45746528866	0.302264736736	547	1.26E-15	6.6E-15
Bacteria	Fusobacteriota	Fusobacteriales	Fusobacteriaceae	Fusobacterium	Fusobacterium vincentii	Pocket.depth	Deep	2.10193545768	0.258548849212	706	1.26E-15	6.6E-15
Bacteria	Actinomycetota	Coriobacteriales	Atopobiaceae	Lancefieldella	Lancefieldella rimae	Pocket.depth	Deep	2.06620341364	0.254495399563	428	1.38E-15	7.1E-15
Bacteria	Bacteroidota	Bacteroidales	CAJPTX01	Bacteroides D	Bacteroides D sp905373475	Pocket.depth	Deep	2.8037353402	0.347543484025	348	2E-15	1E-14
Bacteria	Bacteroidota	Bacteroidales	Bacteroidaceae	Prevotella	Prevotella oralis	Pocket.depth	Deep	2.47588225811	0.307777493918	451	2.44E-15	1.2E-14
Bacteria	Patescibacteria	Saccharimonadales	Nanoperiomorbaceae	Nanoperiomorbus	Nanoperiomorbus sp905372225	Pocket.depth	Deep	2.01960744608	0.251103673151	338	2.47E-15	1.2E-14
Bacteria	Patescibacteria	Saccharimonadales	Nanoperiomorbaceae	Nanoperiomorbus	Nanoperiomorbus periodonticus	Pocket.depth	Deep	1.57950942359	0.196737978972	321	2.76E-15	1.3E-14
Bacteria	Bacillota	Erysipelotrichales	Erysipelotrichaceae	Bulleidia	Bulleidia moorei	Pocket.depth	Deep	1.81009461824	0.225951619477	393	3.15E-15	1.5E-14
Bacteria	Bacillota A	Lachnospirales	Lachnospiraceae	Oribacterium	Oribacterium sp000160135	Pocket.depth	Deep	2.27699882111	0.287900095712	628	6.84E-15	3.2E-14
Bacteria	Pseudomonadota	Pseudomonadales	Moraxellaceae	Acinetobacter	Acinetobacter johnsonii	Pocket.depth	Deep	-1.43498557473	0.182528538564	151	9.77E-15	4.5E-14
Bacteria	Bacteroidota	Bacteroidales	Bacteroidaceae	Prevotella	Prevotella denticola	Pocket.depth	Deep	2.5817075808	0.328708682249	460	1E-14	4.7E-14
Bacteria	Bacteroidota	Bacteroidales	Bacteroidaceae	Prevotella	Prevotella maculosa	Pocket.depth	Deep	2.25476776958	0.287346435762	637	1.09E-14	4.8E-14
Bacteria	Bacteroidota	Bacteroidales	Bacteroidaceae	Prevotella	Prevotella marshii	Pocket.depth	Deep	1.51277794553	0.192771986987	282	1.09E-14	4.8E-14
Bacteria	Bacteroidota	Bacteroidales	Bacteroidaceae	Prevotella	Prevotella oris	Pocket.depth	Deep	2.35098709876	0.306050202695	759	3.73E-14	1.6E-13
Bacteria	Bacillota C	Selenomonadales	Selenomonadaceae	Centipeda	Centipeda sp001683335	Pocket.depth	Deep	1.63453911249	0.217309607506	322	1.2E-13	5.2E-13
Bacteria	Bacillota A	Lachnospirales	Lachnospiraceae	Shuttleworthia	Shuttleworthia satelles	Pocket.depth	Deep	1.31638492857	0.175782694289	196	1.53E-13	6.5E-13
Bacteria	Bacteroidota	Bacteroidales	CAJPTX01	Bacteroides D	Bacteroides D sp013333835	Pocket.depth	Deep	2.27416286199	0.305191596298	481	2E-13	8.3E-13
Bacteria	Patescibacteria	Saccharimonadales	Nanoperiomorbaceae	Nanoperiomorbus	Nanoperiomorbus sp905373275	Pocket.depth	Deep	2.29983189027	0.311749318774	432	3.39E-13	1.4E-12

Bacteria	Fusobacteriota	Fusobacteriales	Fusobacteriaceae	Fusobacterium	Fusobacterium animalis	Pocket.depth	Deep	1.5644450991	0.214509430259	673	6.15E-13	2.5E-12
Bacteria	Bacteroidota	Bacteroidales	Bacteroidaceae	Phocaeicola	Phocaeicola abscessus	Pocket.depth	Deep	1.50793600978	0.209444690797	165	1.18E-12	4.8E-12
Bacteria	Bacillota A	Peptostreptococcales	Anaerovoracaceae	Eubacterium N	Eubacterium N saphenum	Pocket.depth	Deep	1.78397885174	0.248807011824	234	1.46E-12	5.8E-12
Bacteria	Bacillota C	Selenomonadales	Selenomonadaceae	Centipeda	Centipeda flueggei	Pocket.depth	Deep	1.47883386961	0.209465622875	302	3.11E-12	1.2E-11
Bacteria	Actinomycetota	Coriobacteriales	Atopobiaceae	Olsenella F	Olsenella F sp001189515	Pocket.depth	Deep	1.87096330089	0.265271954078	692	3.26E-12	1.3E-11
Bacteria	Actinomycetota	Actinomycetales	Actinomycetaceae	Actinomyces	Actinomyces oris A	Pocket.depth	Deep	-1.99608169148	0.284080747936	541	3.91E-12	1.5E-11
Bacteria	Actinomycetota	Actinomycetales	Actinomycetaceae	Actinomyces	Actinomyces dentalis	Pocket.depth	Deep	1.40605474608	0.200968473719	913	4.8E-12	1.8E-11
Bacteria	Bacillota A	Lachnospirales	Lachnospiraceae	Catonella	Catonella sp916438525	Pocket.depth	Deep	1.20855580864	0.173282593463	276	5.57E-12	2.1E-11
Bacteria	Bacillota	Mycoplasmatales	Metamycoplasmataceae	Metamycoplasma	Metamycoplasma salivarium	Pocket.depth	Deep	1.61148043548	0.234994109966	341	1.23E-11	4.5E-11
Bacteria	Actinomycetota	Actinomycetales	Actinomycetaceae	Actinomyces	Actinomyces israelii	Pocket.depth	Deep	1.73335927609	0.253007395339	789	1.28E-11	4.6E-11
Bacteria	Bacteroidota	Bacteroidales	Bacteroidaceae	Prevotella	Prevotella koreensis	Pocket.depth	Deep	1.12872390587	0.165040859463	163	1.39E-11	4.9E-11
Bacteria	Bacillota A	Peptostreptococcales	Filifactoraceae	Peptoanaerobacter	Peptoanaerobacter stomatis	Pocket.depth	Deep	0.99248433772	0.14676812792	124	2.31E-11	8.1E-11
Bacteria	Bacillota A	Peptostreptococcales	Anaerovoracaceae	Gallibacter	Gallibacter brachus	Pocket.depth	Deep	1.8528719327	0.274504189386	773	2.5E-11	8.6E-11
Bacteria	Bacillota	Erysipelotrichales	Erysipelotrichaceae	Bulleidia	Bulleidia extructa	Pocket.depth	Deep	1.37186549051	0.204351708376	262	3.18E-11	1.1E-10
Bacteria	Bacillota A	Peptostreptococcales	Anaerovoracaceae	Hornefia	Hornefia minuta	Pocket.depth	Deep	1.08691037073	0.163547476797	136	4.95E-11	1.7E-10
Bacteria	Patescibacteria	Saccharimonadales	Saccharimonadaceae	Saccharimonas	Saccharimonas sp018127705	Pocket.depth	Deep	1.40828868406	0.215820719629	303	1.08E-10	3.5E-10
Bacteria	Bacillota A	Lachnospirales	Lachnospiraceae	UBA4285	UBA4285 sp900542465	Pocket.depth	Deep	1.03054072319	0.158570932171	156	1.27E-10	4.1E-10
Bacteria	Bacillota	Lactobacillales	Streptococcaceae	Streptococcus	Streptococcus sanguinis	Pocket.depth	Deep	-1.08192567901	0.169002374025	147	2.36E-10	7.4E-10
Bacteria	Bacillota C	Selenomonadales	Selenomonadaceae	Centipeda	Centipeda sp001717585	Pocket.depth	Deep	1.16452111901	0.182694757145	359	2.8E-10	8.8E-10
Bacteria	Bacteroidota	Bacteroidales	Bacteroidaceae	Prevotella	Prevotella enoeca	Pocket.depth	Deep	0.9911598999	0.156636013607	110	3.75E-10	1.2E-09
Bacteria	Actinomycetota	Mycobacteriales	Mycobacteriaceae	Corynebacterium	Corynebacterium durum	Pocket.depth	Deep	-2.1156590563	0.344937783739	682	1.24E-09	3.8E-09
Bacteria	Bacillota	Lactobacillales	Streptococcaceae	Streptococcus	Streptococcus constellatus	Pocket.depth	Deep	1.38056371473	0.226429020551	241	1.54E-09	4.6E-09
Bacteria	Bacillota A	Peptostreptococcales	Peptostreptococcaceae	Peptostreptococcus	Peptostreptococcus stomatis	Pocket.depth	Deep	1.30888825471	0.21487340701	215	1.59E-09	4.8E-09
Bacteria	Bacteroidota	Bacteroidales	Bacteroidaceae	Prevotella	Prevotella pleuritidis	Pocket.depth	Deep	2.2895300014	0.378830634178	555	2.12E-09	6.2E-09
Bacteria	Bacteroidota	Bacteroidales	Bacteroidaceae	Prevotella	Prevotella nigrescens	Pocket.depth	Deep	1.88802936512	0.318578605699	734	4.26E-09	1.2E-08
Bacteria	Bacillota C	Veillonellales	Megasphaeraceae	Anaeroglobus	Anaeroglobus massiliensis	Pocket.depth	Deep	1.2037054947	0.203149631899	172	4.29E-09	1.2E-08
Bacteria	Bacteroidota	Bacteroidales	Bacteroidaceae	Prevotella	Prevotella conceptionensis	Pocket.depth	Deep	1.7860942608	0.305984680851	681	7.17E-09	2E-08
Bacteria	Patescibacteria	Saccharimonadales	Saccharimonadaceae	Saccharimonas	Saccharimonas sp905372095	Pocket.depth	Deep	0.74464598887	0.128697486945	183	9.64E-09	2.6E-08
Bacteria	Spirochaetota	Treponematales	Treponemataceae	Treponema D	Treponema D sp014334335	Pocket.depth	Deep	1.02084024433	0.177309353551	107	1.14E-08	3.1E-08
Bacteria	Bacillota C	Selenomonadales	Selenomonadaceae	Centipeda	Centipeda infelix	Pocket.depth	Deep	1.28765393199	0.228489052223	464	2.27E-08	6E-08
Bacteria	Pseudomonadota	Burkholderiales	Burkholderiaceae A	Lautropia	Lautropia mirabilis	Pocket.depth	Deep	-1.8193266153	0.328965728277	589	4.08E-08	1.1E-07
Bacteria	Bacillota	Lactobacillales	Streptococcaceae	Streptococcus	Streptococcus gordonii	Pocket.depth	Deep	-1.67138159291	0.313612264727	745	1.22E-07	3.2E-07
Bacteria	Actinomycetota	Actinomycetales	Actinomycetaceae	Actinomyces	Actinomyces sp002999235	Pocket.depth	Deep	1.63908515414	0.309316354548	497	1.43E-07	3.7E-07
Bacteria	Pseudomonadota	Burkholderiales	Burkholderiaceae	Burkholderia	Burkholderia sp018375725	Pocket.depth	Deep	-1.09874616555	0.207978518089	363	1.56E-07	4E-07
Bacteria	Bacillota C	Selenomonadales	Selenomonadaceae	Centipeda	Centipeda felix	Pocket.depth	Deep	1.19715151538	0.228332959002	369	1.93E-07	4.9E-07
Bacteria	Campylobacterota	Campylobacterales	Campylobacteraceae	Campylobacter B	Campylobacter B gracilis A	Pocket.depth	Deep	0.86487371495	0.166012022369	422	2.3E-07	5.7E-07
Bacteria	Bacteroidota	Bacteroidales	Bacteroidaceae	Prevotella	Prevotella illustrans	Pocket.depth	Deep	0.60193757749	0.116886967745	103	3.14E-07	7.7E-07
Bacteria	Bacteroidota	Bacteroidales	Bacteroidaceae	Prevotella	Prevotella sp018127805	Pocket.depth	Deep	0.84834230638	0.166764618745	124	4.34E-07	1E-06

Bacteria	Campylobacterota	Campylobacterales	Campylobacteraceae	Campylobacter B	Campylobacter B sp905373215	Pocket.depth	Deep	0.92335803475	0.182994266659	581	5.36E-07	1.3E-06
Bacteria	Bacteroidota	Bacteroidales	Bacteroidaceae	Prevotella	Prevotella oulorum	Pocket.depth	Deep	1.4005612439	0.279383371777	467	6.33E-07	1.5E-06
Bacteria	Spirochaetota	Treponematales	Treponemataceae	Treponema C	Treponema C lecithinolyticum	Pocket.depth	Deep	1.54092654964	0.308776565879	426	7.1E-07	1.7E-06
Bacteria	Bacillota	Lactobacillales	Streptococcaceae	Streptococcus	Streptococcus intermedius	Pocket.depth	Deep	-1.42950807561	0.286763400494	631	7.3E-07	1.7E-06
Bacteria	Actinomycetota	Propionibacteriales	Propionibacteriaceae	Cutibacterium	Cutibacterium acnes	Pocket.depth	Deep	-1.00061409384	0.20288475353	215	9.53E-07	2.2E-06
Bacteria	Bacteroidota	Bacteroidales	Bacteroidaceae	Prevotella	Prevotella veroralis	Pocket.depth	Deep	0.74971497232	0.157241627068	141	2.14E-06	4.9E-06
Bacteria	Patescibacteria	Saccharimonadales	Saccharimonadaceae	Saccharimonas	Saccharimonas sp905373835	Pocket.depth	Deep	-1.03446864463	0.217979804457	252	2.38E-06	5.4E-06
Bacteria	Actinomycetota	Actinomycetales	Actinomycetaceae	Actinomyces	Actinomyces oris E	Pocket.depth	Deep	-1.32856024299	0.280120030908	528	2.41E-06	5.4E-06
Bacteria	Actinomycetota	Mycobacteriales	Mycobacteriaceae	Corynebacterium	Corynebacterium matruchotii	Pocket.depth	Deep	-1.22249849818	0.258093013813	881	2.49E-06	5.6E-06
Bacteria	Bacteroidota	Bacteroidales	Porphyromonadaceae	Porphyromonas	Porphyromonas gingivalis	Pocket.depth	Deep	1.42112726572	0.306447218266	203	4E-06	8.7E-06
Bacteria	Bacteroidota	Bacteroidales	Bacteroidaceae	Prevotella	Prevotella sp000467895	Pocket.depth	Deep	0.85847498261	0.185067225222	145	4E-06	8.7E-06
Bacteria	Bacillota C	Selenomonadales	Selenomonadaceae	AWVT01	AWVT01 sp916439735	Pocket.depth	Deep	0.94972412333	0.20587596538	186	4.48E-06	9.7E-06
Bacteria	Bacteroidota	Bacteroidales	Tannerellaceae	Tannerella	Tannerella serpentiformis	Pocket.depth	Deep	1.38411252053	0.300483036788	643	4.63E-06	1E-05
Bacteria	Bacillota C	Selenomonadales	Selenomonadaceae	Centipeda	Centipeda sp905372865	Pocket.depth	Deep	0.93184316728	0.202477265315	336	4.72E-06	1E-05
Bacteria	Fusobacteriota	Fusobacteriales	Fusobacteriaceae	Fusobacterium	Fusobacterium nucleatum D	Pocket.depth	Deep	0.49368716357	0.109465961367	134	7.25E-06	1.5E-05
Bacteria	Actinomycetota	Actinomycetales	Actinomycetaceae	Actinomyces	Actinomyces sp001278845	Pocket.depth	Deep	1.05614734959	0.24247361577	652	1.46E-05	3.1E-05
Bacteria	Actinomycetota	Actinomycetales	Bifidobacteriaceae	Bifidobacterium	Bifidobacterium dentium	Pocket.depth	Deep	0.94771232439	0.217778341479	167	1.49E-05	3.1E-05
Bacteria	Fusobacteriota	Fusobacteriales	Fusobacteriaceae	Fusobacterium	Fusobacterium nucleatum	Pocket.depth	Deep	1.13071139413	0.261222786944	496	1.65E-05	3.4E-05
Bacteria	Bacteroidota	Bacteroidales	Bacteroidaceae	Prevotella	Prevotella saccharolytica	Pocket.depth	Deep	1.1816130324	0.274429084388	511	1.83E-05	3.8E-05
Bacteria	Actinomycetota	Actinomycetales	Actinomycetaceae	Actinomyces	Actinomyces johnsonii	Pocket.depth	Deep	-1.15425099048	0.273460012285	628	2.65E-05	5.5E-05
Bacteria	Actinomycetota	Actinomycetales	Actinomycetaceae	Actinomyces	Actinomyces oris B	Pocket.depth	Deep	-1.40806105066	0.335462845984	703	2.94E-05	5.9E-05
Bacteria	Campylobacterota	Campylobacterales	Campylobacteraceae	Campylobacter B	Campylobacter B sp905373295	Pocket.depth	Deep	0.85305613789	0.204241115881	524	3.22E-05	6.4E-05
Bacteria	Campylobacterota	Campylobacterales	Campylobacteraceae	Campylobacter A	Campylobacter A massiliensis	Pocket.depth	Deep	0.47364458051	0.114868194197	157	4E-05	8E-05
Bacteria	Campylobacterota	Campylobacterales	Campylobacteraceae	Campylobacter B	Campylobacter B sp900539505	Pocket.depth	Deep	0.70084963241	0.170411492605	521	4.23E-05	8.3E-05
Bacteria	Patescibacteria	Saccharimonadales	Nanosynbacteraceae	Nanosynbacter	Nanosynbacter sp905373795	Pocket.depth	Deep	0.41943666316	0.102337488223	147	4.49E-05	8.8E-05
Bacteria	Patescibacteria	Saccharimonadales	SDRK01	SDRK01	SDRK01 sp007845205	Pocket.depth	Deep	0.92349058683	0.228219360782	205	5.6E-05	0.00011
Bacteria	Actinomycetota	Actinomycetales	Actinomycetaceae	Actinomyces	Actinomyces sp900323545	Pocket.depth	Deep	-0.89142059555	0.221245567079	150	6E-05	0.00012
Bacteria	Fusobacteriota	Fusobacteriales	Leptotrichiaceae	Leptotrichia	Leptotrichia sp002240055	Pocket.depth	Deep	0.67437639638	0.168094932043	229	6.47E-05	0.00012
Bacteria	Actinomycetota	Actinomycetales	Actinomycetaceae	Actinomyces	Actinomyces sp000195595	Pocket.depth	Deep	-1.14018189811	0.284489740969	436	6.58E-05	0.00013
Bacteria	Bacillota A	Peptostreptococcales	Anaerovoracaceae	Mogibacterium	Mogibacterium diversum	Pocket.depth	Deep	0.78116777051	0.195816581892	267	7.11E-05	0.00013
Bacteria	Patescibacteria	Saccharimonadales	Saccharimonadaceae	Saccharimonas	Saccharimonas sp013333675	Pocket.depth	Deep	0.45932794603	0.116281537452	119	8.36E-05	0.00016
Bacteria	Bacteroidota	Bacteroidales	Bacteroidaceae	Alloprevotella	Alloprevotella sp003639005	Pocket.depth	Deep	0.69617140451	0.178735746662	170	0.0001	0.00019
Bacteria	Bacillota	RF39	UBA660	CAJPPJ01	CAJPPJ01 sp905372515	Pocket.depth	Deep	0.5435501818	0.141731751256	106	0.00013	0.00025
Bacteria	Bacteroidota	Bacteroidales	Bacteroidaceae	Prevotella	Prevotella intermedia	Pocket.depth	Deep	0.99615324695	0.264527954232	230	0.00018	0.00032
Bacteria	Spirochaetota	Treponematales	Treponemataceae	Treponema	Treponema sp905373565	Pocket.depth	Deep	0.57064787804	0.154638213205	101	0.00024	0.00043
Bacteria	Patescibacteria	Saccharimonadales	Saccharimonadaceae	Saccharimonas	Saccharimonas sp905365145	Pocket.depth	Deep	0.32165449289	0.088778121408	101	0.00031	0.00056
Bacteria	Bacteroidota	Flavobacteriales	Flavobacteriaceae	Capnocytophaga	Capnocytophaga gingivalis	Pocket.depth	Deep	-0.98467933889	0.272337692431	733	0.00031	0.00057
Bacteria	Bacillota	Lactobacillales	Streptococcaceae	Streptococcus	Streptococcus sp000220065	Pocket.depth	Deep	-0.56438376084	0.157328463137	190	0.00035	0.00063

Bacteria	Bacillota C	Selenomonadales	Selenomonadaceae	Centipeda	Centipeda timonae	Pocket.depth	Deep	0.62383126092	0.174716731141	317	0.00037	0.00067
Bacteria	Actinomycetota	Coriobacteriales	Eggerthellaceae	Cryptobacterium	Cryptobacterium curtum	Pocket.depth	Deep	0.33950644051	0.095340024655	102	0.00039	0.00069
Bacteria	Pseudomonadota	Burkholderiales	Neisseriaceae	Eikenella	Eikenella halliae	Pocket.depth	Deep	0.58596013691	0.168288201997	223	0.00052	0.00092
Bacteria	Bacillota A	Lachnospirales	Lachnospiraceae	Johnsonella	Johnsonella sp905373785	Pocket.depth	Deep	0.58166050577	0.169862735645	195	0.00064	0.00112
Bacteria	Actinomycetota	Actinomycetales	Micrococcaceae	Rothia	Rothia aeria	Pocket.depth	Deep	-1.10875728957	0.32491318483	580	0.00067	0.00117
Bacteria	Pseudomonadota	Enterobacterales A	Pasteurellaceae	Aggregatibacter	Aggregatibacter sp000466335	Pocket.depth	Deep	0.95843825623	0.281753665619	440	0.0007	0.0012
Bacteria	Fusobacteriota	Fusobacteriales	Leptotrichiaceae	Leptotrichia	Leptotrichia sp013394795	Pocket.depth	Deep	0.45164461623	0.133111500532	157	0.00072	0.00123
Bacteria	Bacillota	Staphylococcales	Gemellaceae	Gemella	Gemella haemolysans A	Pocket.depth	Deep	-0.6404324954	0.18989316357	165	0.00077	0.00132
Bacteria	Bacillota C	Veillonellales	Megasphaeraceae	Anaeroglobus	Anaeroglobus micronuciformis	Pocket.depth	Deep	0.54096426906	0.160600604576	179	0.00078	0.00133
Bacteria	Campylobacterota	Campylobacterales	Campylobacteraceae	Campylobacter A	Campylobacter A curvus	Pocket.depth	Deep	0.47460554413	0.143432454803	128	0.00097	0.00164
Bacteria	Pseudomonadota	Cardiobacteriales	Cardiobacteriaceae	Cardiobacterium	Cardiobacterium hominis	Pocket.depth	Deep	-0.93366601567	0.294270588687	513	0.00156	0.00259
Bacteria	Actinomycetota	Actinomycetales	Actinomycetaceae	Actinomyces	Actinomyces oris D	Pocket.depth	Deep	-0.73072586033	0.238120584919	359	0.00221	0.00366
Bacteria	Patescibacteria	Saccharimonadales	Saccharimonadaceae	Saccharimonas	Saccharimonas sp015257665	Pocket.depth	Deep	-0.43753423931	0.143138842112	153	0.0023	0.00379
Bacteria	Bacillota	Lactobacillales	Streptococcaceae	Streptococcus	Streptococcus mutans	Pocket.depth	Deep	-0.84146349948	0.275503855727	310	0.00232	0.0038
Bacteria	Pseudomonadota	Burkholderiales	Neisseriaceae	Eikenella	Eikenella exigua	Pocket.depth	Deep	0.72951993926	0.246518663852	492	0.00316	0.00516
Bacteria	Actinomycetota	Propionibacteriales	Propionibacteriaceae	Arachnia	Arachnia rubra	Pocket.depth	Deep	-0.81109851099	0.278086816329	331	0.00362	0.00588
Bacteria	Bacillota A	Lachnospirales	Lachnospiraceae	Lachnoanaerobaculum	Lachnoanaerobaculum orale	Pocket.depth	Deep	0.53104355691	0.182685681098	208	0.00373	0.00604
Bacteria	Bacillota A	Lachnospirales	Lachnospiraceae	F0428	F0428 sp003043955	Pocket.depth	Deep	0.43420038629	0.15004764579	137	0.00389	0.00626
Bacteria	Actinomycetota	Actinomycetales	Actinomycetaceae	Pauljensenia	Pauljensenia odontolytica A	Pocket.depth	Deep	-0.43452679529	0.151285828879	179	0.00416	0.00667
Bacteria	Bacteroidota	Bacteroidales	Bacteroidaceae	Prevotella	Prevotella salivae	Pocket.depth	Deep	0.76819142205	0.269013068375	386	0.00438	0.00699
Bacteria	Actinomycetota	Actinomycetales	Actinomycetaceae	Pauljensenia	Pauljensenia sp000185285	Pocket.depth	Deep	-0.73065977027	0.256222780219	526	0.00444	0.00705
Bacteria	Pseudomonadota	Burkholderiales	Neisseriaceae	Neisseria	Neisseria elongata	Pocket.depth	Deep	-0.71114951224	0.256846254226	466	0.00573	0.00901
Bacteria	Actinomycetota	Actinomycetales	Actinomycetaceae	Pauljensenia	Pauljensenia pyogenes A	Pocket.depth	Deep	-0.60380437532	0.218530023621	212	0.00583	0.00913
Bacteria	Bacillota A	Peptostreptococcales	Filifactoraceae	Peptoanaerobacter	Peptoanaerobacter yurii	Pocket.depth	Deep	0.73759710897	0.274460536835	447	0.00732	0.01135
Bacteria	Actinomycetota	Actinomycetales	Actinomycetaceae	Pauljensenia	Pauljensenia hongkongensis	Pocket.depth	Deep	-0.6261712693	0.237488466102	598	0.0085	0.01313
Bacteria	Bacteroidota	Flavobacteriales	Weeksellaceae	JABCPE02	JABCPE02 sp013333875	Pocket.depth	Deep	-0.54284516654	0.207769603452	258	0.00912	0.01401
Bacteria	Fusobacteriota	Fusobacteriales	Leptotrichiaceae	Leptotrichia	Leptotrichia wadei A	Pocket.depth	Deep	0.58086049432	0.231229525217	257	0.01216	0.0186
Bacteria	Bacteroidota	Bacteroidales	Bacteroidaceae	Prevotella	Prevotella melaninogenica B	Pocket.depth	Deep	0.49588234703	0.200594906936	257	0.0136	0.02071
Bacteria	Patescibacteria	Saccharimonadales	Nanosyncoccaceae	Nanosyncoccus	Nanosyncoccus sp905372495	Pocket.depth	Deep	0.29405526521	0.120761849363	109	0.01507	0.02284
Bacteria	Campylobacterota	Campylobacterales	Campylobacteraceae	Campylobacter A	Campylobacter A showae E	Pocket.depth	Deep	0.35401734202	0.145874847372	159	0.01541	0.02325
Bacteria	Bacillota	Staphylococcales	Gemellaceae	Gemella	Gemella morbillorum	Pocket.depth	Deep	-0.5961752938	0.252225649328	617	0.01829	0.02747
Bacteria	Bacteroidota	Bacteroidales	Porphyromonadaceae	Porphyromonas	Porphyromonas sp000467855	Pocket.depth	Deep	0.59891990182	0.255008619122	242	0.01904	0.02847
Bacteria	Actinomycetota	Actinomycetales	Actinomycetaceae	Actinomyces	Actinomyces sp000220835	Pocket.depth	Deep	-0.80255493839	0.34199695044	565	0.01914	0.02849
Bacteria	Bacillota A	Peptostreptococcales	Filifactoraceae	Peptoanaerobacter	Peptoanaerobacter margaretiae	Pocket.depth	Deep	0.31107133055	0.138405828561	163	0.02482	0.03672
Bacteria	Actinomycetota	Coriobacteriales	Atopobiaceae	Lancefieldella	Lancefieldella sp015258715	Pocket.depth	Deep	-0.44098373587	0.196290351694	264	0.02488	0.03672
Bacteria	Bacteroidota	Flavobacteriales	Weeksellaceae	JABCPE02	JABCPE02 sp013333255	Pocket.depth	Deep	-0.34975403741	0.156099032841	190	0.02527	0.03713
Bacteria	Pseudomonadota	Enterobacterales A	Pasteurellaceae	Haemophilus	Haemophilus seminalis	Pocket.depth	Deep	-0.40629984897	0.181592226487	202	0.02548	0.03727
Bacteria	Actinomycetota	Actinomycetales	Actinomycetaceae	Actinomyces	Actinomyces gerencseriae	Pocket.depth	Deep	-0.54919851278	0.246165972008	846	0.0259	0.03756

Bacteria	Bacteroidota	Bacteroidales	Bacteroidaceae	Alloprevotella	Alloprevotella sp905369775	Pocket.depth	Deep	0.35255936884	0.159580045947	168	0.02738	0.03954
Bacteria	Campylobacterota	Campylobacterales	Campylobacteraceae	Campylobacter A	Campylobacter A showae D	Pocket.depth	Deep	0.29423591632	0.133779802025	128	0.02808	0.04037
Bacteria	Fusobacteriota	Fusobacteriales	Fusobacteriaceae	Fusobacterium	Fusobacterium polymorphum	Pocket.depth	Deep	-0.49536575576	0.226007527605	664	0.02862	0.04098
Bacteria	Actinomycetota	Actinomycetales	Actinomycetaceae	Pauljensenia	Pauljensenia sp000278725	Pocket.depth	Deep	-0.42165169227	0.195849768266	253	0.03156	0.04484
Bacteria	Patescibacteria	Saccharimonadales	Nanosynbacteraceae	Nanosynbacter	Nanosynbacter sp905373315	Pocket.depth	Deep	-0.55025790292	0.255622688996	325	0.03159	0.04484
Bacteria	Fusobacteriota	Fusobacteriales	Leptotrichiaceae	Leptotrichia	Leptotrichia hongkongensis	Pocket.depth	Deep	-0.42040715884	0.199806502578	400	0.03562	0.04972
Bacteria	Fusobacteriota	Fusobacteriales	Leptotrichiaceae	Leptotrichia	Leptotrichia buccalis	Pocket.depth	Deep	0.41420940335	0.196820264885	282	0.03558	0.04972
Bacteria	Bacillota	Staphylococcales	Gemellaceae	Gemella	Gemella haemolysans B	Pocket.depth	Deep	-0.34086205203	0.162316690237	128	0.03598	0.05001
Bacteria	Actinomycetota	Actinomycetales	Actinomycetaceae	Pauljensenia	Pauljensenia sp916439125	Pocket.depth	Deep	-0.23146943341	0.111211987632	104	0.03766	0.05213
Bacteria	Actinomycetota	Actinomycetales	Actinomycetaceae	Pauljensenia	Pauljensenia georgiae	Pocket.depth	Deep	-0.47061939385	0.227203916766	366	0.03858	0.05319
Bacteria	Campylobacterota	Campylobacterales	Campylobacteraceae	Campylobacter B	Campylobacter B gracilis	Pocket.depth	Deep	0.41487430503	0.204825458772	838	0.04308	0.05915
Bacteria	Pseudomonadota	Burkholderiales	Burkholderiaceae A	Lautropia	Lautropia dentalis	Pocket.depth	Deep	-0.50819870214	0.25147766552	293	0.04356	0.05956
Bacteria	Bacillota C	Selenomonadales	Selenomonadaceae	Centipeda	Centipeda artemidis	Pocket.depth	Deep	-0.55136602228	0.27347322061	525	0.04405	0.05999
Bacteria	Bacteroidota	Flavobacteriales	Flavobacteriaceae	Capnocytophaga	Capnocytophaga sp905372595	Pocket.depth	Deep	-0.28719778734	0.149203965673	125	0.05453	0.07396
Bacteria	Patescibacteria	Saccharimonadales	Nanosynbacteraceae	Nanosynbacter	Nanosynbacter lyticus	Pocket.depth	Deep	0.29750703127	0.154963646399	168	0.05516	0.07451
Bacteria	Pseudomonadota	Burkholderiales	Neisseriaceae	Eikenella	Eikenella glucosivorans	Pocket.depth	Deep	0.26251398678	0.137770631382	162	0.05701	0.0767
Bacteria	Bacillota A	Lachnospirales	Lachnospiraceae	Johnsonella	Johnsonella ignava	Pocket.depth	Deep	0.44851082024	0.242693903716	413	0.06489	0.08695
Bacteria	Bacillota	Staphylococcales	Gemellaceae	Gemella	Gemella sanguinis	Pocket.depth	Deep	-0.338970314	0.185271125397	238	0.06761	0.09023
Bacteria	Bacteroidota	Flavobacteriales	Flavobacteriaceae	Capnocytophaga	Capnocytophaga granulosa	Pocket.depth	Deep	0.47083618656	0.261757926108	478	0.07236	0.09581
Bacteria	Bacteroidota	Bacteroidales	Bacteroidaceae	Prevotella	Prevotella pallens	Pocket.depth	Deep	0.38397918078	0.216149496699	174	0.07596	0.10019
Bacteria	Pseudomonadota	Burkholderiales	Neisseriaceae	Neisseria	Neisseria bacilliformis	Pocket.depth	Deep	-0.31935891889	0.185744316982	203	0.08586	0.11236
Bacteria	Actinomycetota	Propionibacteriales	Propionibacteriaceae	Arachnia	Arachnia sp013333945	Pocket.depth	Deep	-0.3832274413	0.232156153933	221	0.09911	0.12869
Bacteria	Bacteroidota	Flavobacteriales	Flavobacteriaceae	Capnocytophaga	Capnocytophaga haemolytica	Pocket.depth	Deep	-0.31257774611	0.191185319336	173	0.10238	0.13242
Bacteria	Actinomycetota	Actinomycetales	Actinomycetaceae	Actinomyces	Actinomyces oris C	Pocket.depth	Deep	-0.37903774253	0.232685005052	432	0.10363	0.13353
Bacteria	Bacillota C	Selenomonadales	Selenomonadaceae	Centipeda	Centipeda noxia	Pocket.depth	Deep	0.4332606997	0.269319519813	692	0.10799	0.13861
Bacteria	Pseudomonadota	Burkholderiales	Neisseriaceae	Eikenella	Eikenella corrodens	Pocket.depth	Deep	0.35140970654	0.22333974931	571	0.11594	0.14799
Bacteria	Actinomycetota	Propionibacteriales	Propionibacteriaceae	Arachnia	Arachnia massiliensis	Pocket.depth	Deep	-0.27006622092	0.171756812775	137	0.11618	0.14799
Bacteria	Actinomycetota	Actinomycetales	Actinomycetaceae	Peptidiphaga	Peptidiphaga sp000466165	Pocket.depth	Deep	-0.48193822785	0.313094630838	842	0.12405	0.15682
Bacteria	Actinomycetota	Actinomycetales	Bifidobacteriaceae	Scardovia	Scardovia wiggsiae	Pocket.depth	Deep	0.44483827136	0.291564147079	218	0.1274	0.16045
Bacteria	Bacteroidota	Flavobacteriales	Flavobacteriaceae	Capnocytophaga	Capnocytophaga bilenii	Pocket.depth	Deep	0.21926558035	0.145425046434	121	0.13193	0.16551
Bacteria	Campylobacterota	Campylobacterales	Campylobacteraceae	Campylobacter A	Campylobacter A sp905371695	Pocket.depth	Deep	-0.23556091365	0.156425538434	153	0.13241	0.16551
Bacteria	Actinomycetota	Mycobacteriales	Mycobacteriaceae	Corynebacterium	Corynebacterium sp915275065	Pocket.depth	Deep	-0.21635874061	0.146420782017	101	0.13982	0.17412
Bacteria	Fusobacteriota	Fusobacteriales	Leptotrichiaceae	Leptotrichia A	Leptotrichia A sp000469505	Pocket.depth	Deep	-0.25384750804	0.173369629304	276	0.14345	0.17799
Bacteria	Fusobacteriota	Fusobacteriales	Leptotrichiaceae	Leptotrichia	Leptotrichia shahii	Pocket.depth	Deep	0.2058007056	0.142314357897	144	0.14846	0.18352
Bacteria	Bacillota C	Veillonellales	Veillonellaceae	Veillonella	Veillonella atypica	Pocket.depth	Deep	-0.21628741189	0.150192073352	130	0.15016	0.18444
Bacteria	Actinomycetota	Actinomycetales	Actinomycetaceae	Pauljensenia	Pauljensenia sp000466265	Pocket.depth	Deep	-0.20059479706	0.139343622677	117	0.1503	0.18444
Bacteria	Fusobacteriota	Fusobacteriales	Fusobacteriaceae	Fusobacterium	Fusobacterium hwasookii	Pocket.depth	Deep	0.20289689842	0.141831001282	179	0.15287	0.1869
Bacteria	Patescibacteria	Saccharimonadales	Nanosynbacteraceae	Nanosynbacter	Nanosynbacter featherlites	Pocket.depth	Deep	-0.26913662873	0.189640112253	236	0.15615	0.19022

Bacteria	Bacillota A	Lachnospirales	Lachnospiraceae	Johnsonella	Johnsonella sp900766185	Pocket.depth	Deep	0.33668926306	0.239838668896	408	0.16068	0.19503
Bacteria	Actinomycetota	Actinomycetales	Actinomycetaceae	Peptidiphaga	Peptidiphaga sp905373325	Pocket.depth	Deep	-0.22128285457	0.163058100448	179	0.17506	0.21069
Bacteria	Pseudomonadota	Enterobacterales A	Pasteurellaceae	Aggregatibacter	Aggregatibacter actinomycetemc	Pocket.depth	Deep	0.31576786294	0.232290353	142	0.17434	0.21069
Bacteria	Bacillota A	Tissierellales	Peptoniphilaceae	Parvimonas	Parvimonas sp000214475	Pocket.depth	Deep	-0.23664044908	0.174538881018	221	0.17547	0.21069
Bacteria	Fusobacteriota	Fusobacteriales	Leptotrichiaceae	Leptotrichia A	Leptotrichia A sp001274535	Pocket.depth	Deep	-0.32847794281	0.243672692219	374	0.17795	0.21291
Bacteria	Pseudomonadota	Enterobacterales A	Pasteurellaceae	Aggregatibacter	Aggregatibacter aphrophilus	Pocket.depth	Deep	0.35114986848	0.266898842813	434	0.18859	0.22403
Bacteria	Fusobacteriota	Fusobacteriales	Leptotrichiaceae	Pseudoleptotrichia	Pseudoleptotrichia goodfellowii	Pocket.depth	Deep	-0.24091924878	0.186074571112	208	0.19571	0.23167

Supplementary Table 2: Significant taxa associated with shallow pockets

superkingdom	phylum	order	family	genus	species	metadata	value	coef	stderr	N.not.0	pval	qval
Bacteria	Pseudomonadota	Burkholderiales	Burkholderiaceae A	Lautropia	Lautropia dentalis	Pocket.depth	Shallow_d	-1.347910319254	0.275748000061687	369	1.18921E-06	0.0003531963
Bacteria	Bacillota	RF39	UBA660	CAJPPJ01	CAJPPJ01 sp905372515	Pocket.depth	Shallow_d	-0.590848790295	0.147837280446921	100	6.90926E-05	0.0102602478
Bacteria	Actinomycetota	Actinomycetales	Actinomycetaceae	Actinomyces	Actinomyces israelii	Pocket.depth	Shallow_d	0.9579484773906	0.260881594214347	652	0.00025375	0.0251215196
Bacteria	Bacillota	Staphylococcales	Gemellaceae	Gemella	Gemella sanguinis	Pocket.depth	Shallow_d	-0.65147116849	0.19826183165938	280	0.00105273	0.0390827085
Bacteria	Desulfobacterota	Desulfobulbales	Desulfobulbaceae	Desulfobulbus	Desulfobulbus oralis	Pocket.depth	Shallow_d	0.9146014712285	0.27677662688904	244	0.00098616	0.0390827085
Bacteria	Bacillota A	Peptostreptococcales	Anaerovoracaceae	Hornefia	Hornefia nodata	Pocket.depth	Shallow_d	0.7138821564009	0.208843384002837	181	0.00065626	0.0390827085
Bacteria	Bacteroidota	Bacteroidales	Bacteroidaceae	Prevotella	Prevotella sp003043945	Pocket.depth	Shallow_d	0.5902553293631	0.179092652233625	123	0.00101674	0.0390827085
Bacteria	Bacillota A	Peptostreptococcales	Filifactoraceae	Filifactor	Filifactor alocis	Pocket.depth	Shallow_d	0.8083511951773	0.249813663915075	238	0.00125374	0.0413735838
Bacteria	Pseudomonadota	Burkholderiales	Neisseriaceae	Neisseria	Neisseria sp000090875	Pocket.depth	Shallow_d	-0.939854156367	0.308950852925212	403	0.00241229	0.0651317788
Bacteria	Actinomycetota	Coriobacteriales	Atopobiaceae	Olsenella	Olsenella uli	Pocket.depth	Shallow_d	0.4587838736281	0.152885111476996	160	0.0027608	0.068329903
Bacteria	Actinomycetota	Propionibacteriales	Propionibacteriaceae	Arachnia	Arachnia rubra	Pocket.depth	Shallow_d	-1.024234880948	0.34541307897094	393	0.003098	0.07077731
Bacteria	Bacteroidota	Bacteroidales	Tannerellaceae	Tannerella	Tannerella forsythia	Pocket.depth	Shallow_d	0.9052566530823	0.317634391199323	592	0.00446399	0.0855492366
Bacteria	Pseudomonadota	Xanthomonadales	Xanthomonadaceae	Xanthomonas	Xanthomonas campestris	Pocket.depth	Shallow_d	-0.39470228902	0.137866322391863	138	0.00428718	0.0855492366
Bacteria	Bacillota A	Eubacteriales	Eubacteriaceae	Pseudoramibacter	Pseudoramibacter alactolyticus	Pocket.depth	Shallow_d	0.5585523032454	0.196690863180736	174	0.00460871	0.0855492366
Bacteria	Bacteroidota	Bacteroidales	Bacteroidaceae	Prevotella	Prevotella nanceiensis	Pocket.depth	Shallow_d	-0.304024632964	0.110179625447668	123	0.00589995	0.1030756241
Bacteria	Bacteroidota	Bacteroidales	Porphyromonadaceae	Porphyromonas	Porphyromonas endodontalis	Pocket.depth	Shallow_d	0.8821483580812	0.325738684825355	413	0.00688433	0.1135913746
Bacteria	Bacillota A	Lachnospirales	Vallitaleaceae	W11650	W11650 sp002999035	Pocket.depth	Shallow_d	0.3865063799723	0.146627316767538	115	0.00852265	0.1332225257
Bacteria	Bacillota A	Peptostreptococcales	Anaerovoracaceae	Mogibacterium	Mogibacterium timidum	Pocket.depth	Shallow_d	0.650534715969	0.257534417926827	321	0.01169383	0.1667279428
Bacteria	Fusobacteriota	Fusobacteriales	Fusobacteriaceae	Fusobacterium	Fusobacterium nucleatum J	Pocket.depth	Shallow_d	0.4137398769332	0.163977676902031	176	0.01178884	0.1667279428
Bacteria	Pseudomonadota	Enterobacterales A	Pasteurellaceae	Haemophilus	Haemophilus seminalis	Pocket.depth	Shallow_d	-0.536627095465	0.221616542561544	231	0.01564137	0.1760759266
Bacteria	Actinomycetota	Propionibacteriales	Propionibacteriaceae	Arachnia	Arachnia sp013333945	Pocket.depth	Shallow_d	-0.564607534334	0.229810771196542	248	0.01418978	0.1760759266
Bacteria	Synergistota	Synergistales	Aminobacteriaceae	CAJPSE01	CAJPSE01 sp905373185	Pocket.depth	Shallow_d	0.6705317917698	0.276652927125026	276	0.01554295	0.1760759266
Bacteria	Synergistota	Synergistales	Aminobacteriaceae	Fretibacterium	Fretibacterium fastidiosum	Pocket.depth	Shallow_d	0.6581625359844	0.273572594408664	364	0.01632196	0.1760759266
Bacteria	Pseudomonadota	Burkholderiales	Neisseriaceae	Eikenella	Eikenella exigua	Pocket.depth	Shallow_d	0.5488747495082	0.224881459199287	399	0.01483454	0.1760759266
Bacteria	Actinomycetota	Actinomycetales	Actinomycetaceae	Actinomyces	Actinomyces oris E	Pocket.depth	Shallow_d	-0.716572584682	0.299184588489908	576	0.01680416	0.1760759266
Bacteria	Bacteroidota	Bacteroidales	Porphyromonadaceae	Porphyromonas	Porphyromonas pasteri	Pocket.depth	Shallow_d	-0.715106552077	0.302563948769386	542	0.01829892	0.181159305
Bacteria	Actinomycetota	Actinomycetales	Actinomycetaceae	Actinomyces	Actinomyces oris	Pocket.depth	Shallow_d	0.5794053470405	0.248496876268226	755	0.01992235	0.1849042922
Bacteria	Bacteroidota	Bacteroidales	Bacteroidaceae	Prevotella	Prevotella oralis	Pocket.depth	Shallow_d	0.5243162603054	0.223785562832028	284	0.01933247	0.1849042922
Bacteria	Actinomycetota	Actinomycetales	Actinomycetaceae	Actinomyces	Actinomyces sp000195595	Pocket.depth	Shallow_d	-0.627580717639	0.274080618221401	477	0.02224752	0.2002276535
Bacteria	Fusobacteriota	Fusobacteriales	Leptotrichiaceae	Leptotrichia	Leptotrichia hofstadii	Pocket.depth	Shallow_d	-0.52061277312	0.231728891615082	312	0.02488553	0.2111715208
Bacteria	Actinomycetota	Actinomycetales	Actinomycetaceae	Pauljensenia	Pauljensenia sp902373545	Pocket.depth	Shallow_d	-0.383684027822	0.172766724428596	127	0.02659198	0.2193838264

Bacteria	Actinomycetota	Mycobacteriales	Mycobacteriaceae	Corynebacterium	Corynebacterium matruchotii	Pocket.depth	Shallow_d	-0.546732623038	0.248763591990425	872	0.02819762	0.220386626
Bacteria	Fusobacteriota	Fusobacteriales	Leptotrichiaceae	Leptotrichia	Leptotrichia trevisanii	Pocket.depth	Shallow_d	-0.279171325924	0.126952284486006	126	0.02811007	0.220386626
Bacteria	Actinomycetota	Coriobacteriales	Eggerthellaceae	Slackia	Slackia exigua	Pocket.depth	Shallow_d	0.3802421086932	0.174586610427111	278	0.02964806	0.2257813759
Bacteria	Bacillota A	Tissierellales	Peptoniphilaceae	Parvimonas	Parvimonas micra	Pocket.depth	Shallow_d	0.560381881723	0.258894412443935	563	0.03066641	0.2276980899
Bacteria	Bacteroidota	Bacteroidales	Porphyromonadaceae	Porphyromonas	Porphyromonas gingivalis	Pocket.depth	Shallow_d	0.4854887803979	0.227264232052199	143	0.03290886	0.2327126678
Bacteria	Pseudomonadota	Pseudomonadales	Moraxellaceae	Acinetobacter	Acinetobacter guillouiae	Pocket.depth	Shallow_d	-0.478980387638	0.223665172285098	304	0.03248028	0.2327126678

Supplementary Table 3: Significant microbial pathways associated with periodontal pocket depths

Pathway category	Pathway	metadata	value	coef	stderr	N.not.0	pval	qval
Cysteine and methionine metabolism	cysteine biosynthesis serine cysteine	Pocket.depth	Deep	-0.61081554855449	0.03830419896574	1001	3.53093335419E-51	4.51959469336618E-49
Serine and threonine metabolism	threonine biosynthesis aspartate homoserine threonine	Pocket.depth	Deep	-0.50049687129238	0.03235258455409	1002	1.46167810961E-48	9.35473990150518E-47
Central carbohydrate metabolism	pentose phosphate pathway oxidative phase glucose 6p ribulose 5p	Pocket.depth	Deep	-0.7306123459698	0.0475814002658	1002	6.16390421288E-48	1.94462282799713E-46
Aromatic amino acid metabolism	tryptophan biosynthesis chorismate tryptophan	Pocket.depth	Deep	-0.85038908453854	0.05530327285233	1002	4.69350441334E-48	1.94462282799713E-46
Cofactor and vitamin biosynthesis	heme biosynthesis glutamate protoheme siroheme	Pocket.depth	Deep	-0.71946310181675	0.0469064710222	1001	7.59618292186E-48	1.94462282799713E-46
Branched-chain amino acid metabolism	isoleucine biosynthesis threonine 2 oxobutanoate isoleucine	Pocket.depth	Deep	-0.67002875160564	0.04478538560931	1002	8.10293368399E-46	1.72862585258514E-44
Fatty acid metabolism	fatty acid biosynthesis initiation	Pocket.depth	Deep	-0.51411494319059	0.03466374021065	1002	3.95132340814E-45	7.22527708917475E-44
Central carbohydrate metabolism	gluconeogenesis oxaloacetate fructose 6p	Pocket.depth	Deep	0.288834978965525	0.01958911732963	1002	1.13860881819E-44	1.76558253786779E-43
Nitrogen metabolism	nitrogen fixation nitrogen ammonia	Pocket.depth	Deep	3.22786921751652	0.21902333521956	666	1.24142522194E-44	1.76558253786779E-43
Pyrimidine metabolism	pyrimidine degradation uracil beta alanine thymine 3 aminoisobutanoate	Pocket.depth	Deep	1.77062238764275	0.12177262696678	999	1.35188958701E-43	1.73041867136892E-42
Other carbohydrate metabolism	nucleotide sugar biosynthesis galactose udp galactose	Pocket.depth	Deep	-0.76908844515098	0.05338407196966	1002	6.74592605773E-43	7.84980486717365E-42
Pyrimidine metabolism	pyrimidine deoxyribonuleotide biosynthesis cdp ctp dcdp dctp dtdp dttp	Pocket.depth	Deep	-1.07823330651776	0.07678478955901	1000	5.13535281876E-41	5.4777096733489E-40
Central carbohydrate metabolism	prpp biosynthesis ribose 5p prpp	Pocket.depth	Deep	0.341518440278988	0.02438209277512	1002	7.78655444873E-41	7.66676130336913E-40
Branched-chain amino acid metabolism	valine isoleucine biosynthesis pyruvate valine 2 oxobutanoate isoleucine	Pocket.depth	Deep	-0.62225408318291	0.04492315100674	1002	4.81656695234E-40	4.40371835642633E-39
Carbon fixation	cam crassulacean acid metabolism light	Pocket.depth	Deep	2.269870953289	0.16565525098431	985	2.73309234325E-39	2.33223879957734E-38
Sulfur metabolism	assimilatory sulfate reduction sulfate h2s	Pocket.depth	Deep	-1.77027571785549	0.12977974415538	998	5.58789357274E-39	4.47031485818931E-38
Lipopolysaccharide metabolism	lipopolysaccharide biosynthesis kdo2 lipid a	Pocket.depth	Deep	1.12791866970459	0.08288691994301	1000	8.15239600276E-39	6.13827463737035E-38
Terpenoid backbone biosynthesis	c10 c20 isoprenoid biosynthesis bacteria	Pocket.depth	Deep	-1.00239713122508	0.07380748260505	1001	1.10904960844E-38	7.88657499332873E-38
Cofactor and vitamin biosynthesis	nad biosynthesis aspartate nad	Pocket.depth	Deep	1.6130766778836	0.12004117409113	998	5.76780643007E-38	3.88568012131313E-37
Nitrogen metabolism	assimilatory nitrate reduction nitrate ammonia	Pocket.depth	Deep	3.40588937868766	0.2558167351772	720	2.37099025892E-37	1.51743376571127E-36
Lipid metabolism	phosphatidylethanolamine pe biosynthesis pa ps pe	Pocket.depth	Deep	1.1400148795108	0.08639433416851	1000	9.06624246003E-37	5.52609064230369E-36
Cofactor and vitamin biosynthesis	riboflavin biosynthesis gtp riboflavin fmn fad	Pocket.depth	Deep	0.516017055111978	0.0392378143637	1000	1.49776507382E-36	8.71426952037921E-36
Polyamine biosynthesis	polyamine biosynthesis arginine ornithine putrescine	Pocket.depth	Deep	3.82760595886775	0.29355202456337	700	5.28045951455E-36	2.9386905124463E-35
Terpenoid backbone biosynthesis	c5 isoprenoid biosynthesis non mevalonate pathway	Pocket.depth	Deep	0.251449805100375	0.01933405048492	1002	7.67513402086E-36	4.09340481112644E-35
Cofactor and vitamin biosynthesis	menaquinone biosynthesis chorismate menaquinone	Pocket.depth	Deep	-0.41706839083114	0.03262426908449	1002	9.02006369498E-35	4.61827261183122E-34
Central carbohydrate metabolism	pentose phosphate pathway non oxidative phase fructose 6p ribose 5p	Pocket.depth	Deep	0.190085787292973	0.01496611869071	1002	2.25099047612E-34	1.10817992670447E-33
Lipopolysaccharide metabolism	cmp kdo biosynthesis	Pocket.depth	Deep	1.06437662649265	0.08394802822779	1000	2.86960567603E-34	1.36040565381929E-33
Central carbohydrate metabolism	glycolysis embden meyerhof pathway glucose pyruvate	Pocket.depth	Deep	0.158305933453901	0.01294589596456	1002	3.81593600219E-32	1.68427520096464E-31
Aromatics degradation	catechol ortho cleavage catechol 3 oxoadipate	Pocket.depth	Deep	-2.98439927594436	0.24403143763452	600	3.7632982588E-32	1.68427520096464E-31
Other carbohydrate metabolism	nucleotide sugar biosynthesis glucose udp glucose	Pocket.depth	Deep	-0.65572286848893	0.05416007754698	1002	1.38973362121E-31	5.92953011714254E-31
Lysine metabolism	lysine biosynthesis succinyl dap pathway aspartate lysine	Pocket.depth	Deep	-0.33047980538615	0.02731013333065	1002	1.48329225606E-31	6.12456157340125E-31
Aromatics degradation	benzoate degradation benzoate catechol methylbenzoate methylcatechol	Pocket.depth	Deep	-2.95221501968008	0.24497695349816	594	2.52143489745E-31	1.00857395898026E-30
Cofactor and vitamin biosynthesis	thiamine biosynthesis air thiamine p thiamine 2p	Pocket.depth	Deep	1.35828991471692	0.11337669420332	999	5.32193037307E-31	2.06426390227988E-30
Other carbohydrate metabolism	d galacturonate degradation bacteria d galacturonate pyruvate d glyceraldehyde 3p	Pocket.depth	Deep	2.03246696400987	0.17334493780518	957	7.70204163197E-30	2.89959214379954E-29
Other carbohydrate metabolism	d galactonate degradation de ley doudoroff pathway d galactonate glycerate 3p	Pocket.depth	Deep	3.78020045441159	0.32525786927978	721	2.2319362271E-29	8.16250963055043E-29
Terpenoid backbone biosynthesis	c10 c20 isoprenoid biosynthesis archaea	Pocket.depth	Deep	-1.10022836308096	0.09607848624607	999	1.2869777631E-28	4.5759209354712E-28

Central carbohydrate metabolism	semi phosphorylative entner doudoroff pathway gluconate glycerate 3p	Pocket.depth	Deep	1.09784203195592	0.09664617647018	1000	3.27845664509E-28	1.13416878532712E-27
Cofactor and vitamin biosynthesis	biotin biosynthesis pimeloyl acp coa biotin	Pocket.depth	Deep	1.17656081202257	0.10481289590078	1000	1.26830373162E-27	4.27218099071247E-27
Central carbohydrate metabolism	pyruvate oxidation pyruvate acetyl coa	Pocket.depth	Deep	0.133620681198132	0.01190925273215	1002	1.3395289052E-27	4.39640256065244E-27
Cofactor and vitamin biosynthesis	pimeloyl acp biosynthesis bioc bioh pathway malonyl acp pimeloyl acp	Pocket.depth	Deep	0.870069406518249	0.07787311792257	1000	2.14584554833E-27	6.86670575464756E-27
Nitrogen metabolism	denitrification nitrate nitrogen	Pocket.depth	Deep	-1.16978504790813	0.10491673028352	998	2.7079583437E-27	8.45411385351353E-27
Cofactor and vitamin biosynthesis	biotin biosynthesis biow pathway pimelate pimeloyl coa biotin	Pocket.depth	Deep	1.16900759782973	0.1049505035264	1000	3.02242253958E-27	9.21119250156948E-27
Central carbohydrate metabolism	glycolysis core module involving three carbon compounds	Pocket.depth	Deep	0.171730022221747	0.01543811056838	1002	3.50658852679E-27	1.04382170099798E-26
Cofactor and vitamin biosynthesis	biotin biosynthesis bioi pathway long chain acyl acp pimeloyl acp biotin	Pocket.depth	Deep	1.14787716795048	0.1050511899017	1000	2.46856054357E-26	7.18126703583079E-26
Lysine metabolism	lysine degradation lysine saccharopine acetoacetyl coa	Pocket.depth	Deep	-3.07219166109463	0.28640150229529	559	1.74214466925E-25	4.9554337258734E-25
Histidine metabolism	histidine degradation histidine n formiminoglutamate glutamate	Pocket.depth	Deep	0.858916637794893	0.08043614943084	1000	2.79009861113E-25	7.76375265706356E-25
Serine and threonine metabolism	betaine biosynthesis choline betaine	Pocket.depth	Deep	-1.25317840393227	0.1192930318404	1000	1.47304198446E-24	4.01168880873607E-24
Fatty acid metabolism	beta oxidation	Pocket.depth	Deep	-2.69431479579648	0.25703031474496	684	1.82672321198E-24	4.87126189861744E-24
Fatty acid metabolism	fatty acid biosynthesis elongation	Pocket.depth	Deep	0.123867634878255	0.01194791531096	1002	5.44831419199E-24	1.42323309505157E-23
Branched-chain amino acid metabolism	leucine degradation leucine acetoacetate acetyl coa	Pocket.depth	Deep	-2.3884747053089	0.23078303175739	564	6.44906044876E-24	1.6509594748818E-23
Central carbohydrate metabolism	pentose phosphate pathway pentose phosphate cycle	Pocket.depth	Deep	-0.24452141910911	0.02372384192757	1002	9.61882562544E-24	2.4141366275624E-23
Arginine and proline metabolism	proline biosynthesis glutamate proline	Pocket.depth	Deep	-0.41860810558289	0.04270633105282	1002	1.0116012767E-21	2.49009545034258E-21
Histidine metabolism	histidine biosynthesis prpp histidine	Pocket.depth	Deep	-0.4911248432713	0.05029936381448	1000	1.42488297066E-21	3.4412267970558E-21
Other carbohydrate metabolism	glyoxylate cycle	Pocket.depth	Deep	-2.2280507079147	0.23167439960874	909	5.30545426294E-21	1.25758915862283E-20
Purine metabolism	adenine ribonucleotide biosynthesis imp adp atp	Pocket.depth	Deep	0.144175382748501	0.01515721256388	1002	1.34656062708E-20	3.13381382303298E-20
Nitrogen metabolism	dissimilatory nitrate reduction nitrate ammonia	Pocket.depth	Deep	-0.6946758210479	0.07392488288235	1001	3.69161053495E-20	8.43796693701805E-20
Aromatic amino acid metabolism	tyrosine biosynthesis prephanate pretyrosine tyrosine	Pocket.depth	Deep	-1.96373728770017	0.20921207519314	252	4.05297453888E-20	9.10141650837111E-20
Cofactor and vitamin biosynthesis	pyridoxal biosynthesis erythrose 4p pyridoxal 5p	Pocket.depth	Deep	2.14608286707949	0.22891272560101	948	4.46998277578E-20	9.86478957412641E-20
Central carbohydrate metabolism	citrate cycle tca cycle krebs cycle	Pocket.depth	Deep	-0.60523019834574	0.06583475967691	1001	2.15446056875E-19	4.67408394576781E-19
Carbon fixation	cam crassulacean acid metabolism dark	Pocket.depth	Deep	-1.33965738826036	0.14655634244032	993	3.369168864E-19	7.18756024320099E-19
Aromatic amino acid metabolism	shikimate pathway phosphoenolpyruvate erythrose 4p chorismate	Pocket.depth	Deep	0.105596602816565	0.01155512231168	1002	3.43868808237E-19	7.21560777940305E-19
Lipid metabolism	triacylglycerol biosynthesis	Pocket.depth	Deep	-1.83734465359733	0.20323073895769	229	7.89424910384E-19	1.62978046014726E-18
Branched-chain amino acid metabolism	isoleucine biosynthesis pyruvate 2 oxobutanoate	Pocket.depth	Deep	-0.33753552055531	0.03755247980691	1002	1.22743930303E-18	2.49384493313572E-18
Sulfur metabolism	dissimilatory sulfate reduction sulfate h2s	Pocket.depth	Deep	3.12633216378252	0.3505036077176	409	2.18667029844E-18	4.37334059687166E-18
Purine metabolism	purine degradation xanthine urea	Pocket.depth	Deep	-1.86740105542461	0.20967692102043	203	2.44695753072E-18	4.81862406049097E-18
Methane metabolism	f420 biosynthesis	Pocket.depth	Deep	3.39615354044442	0.38999678012753	411	1.25894421216E-17	2.44158877510651E-17
Aromatics degradation	benzene degradation benzene catechol	Pocket.depth	Deep	-1.7430408137056	0.20109598610735	186	1.75289362547E-17	3.34881170239815E-17
Lipid metabolism	phosphatidylcholine pc biosynthesis pe pc	Pocket.depth	Deep	-2.03513019417313	0.23504979349065	731	1.8928686689E-17	3.56304690616346E-17
Other carbohydrate metabolism	malonate semialdehyde pathway propanoyl coa acetyl coa	Pocket.depth	Deep	-1.71395617318078	0.20035968406488	184	4.39971442686E-17	8.16178908171725E-17
Cofactor and vitamin biosynthesis	cobalamin biosynthesis cobinamide cobalamin	Pocket.depth	Deep	0.568279278688894	0.06655192972653	999	4.98648093469E-17	9.11813656629873E-17
Lysine metabolism	lysine biosynthesis mediated by lysw 2 aminoadipate lysine	Pocket.depth	Deep	2.88035912143424	0.34623366131061	391	2.87862542078E-16	5.11755630360841E-16
Arginine and proline metabolism	ornithine biosynthesis mediated by lysw glutamate ornithine	Pocket.depth	Deep	2.88035912143424	0.34623366131061	391	2.87862542078E-16	5.11755630360841E-16
Central carbohydrate metabolism	pentose phosphate pathway archaea fructose 6p ribose 5p	Pocket.depth	Deep	-1.48304591012901	0.17966151543681	949	4.77870496149E-16	8.37909911055262E-16
Carbon fixation	reductive pentose phosphate cycle glyceraldehyde 3p ribulose 5p	Pocket.depth	Deep	0.129390726713528	0.0161303910356	1002	2.90641350191E-15	5.02730984114329E-15

Other carbohydrate metabolism	trehalose biosynthesis d glucose 1p trehalose	Pocket.depth	Deep	0.307464584716333	0.0389588234944	1002	7.77467701083E-15	1.32687820984834E-14
Central carbohydrate metabolism	entner doudoroff pathway glucose 6p glyceraldehyde 3p pyruvate	Pocket.depth	Deep	0.754647168107642	0.09918988837954	997	6.40778739863E-14	1.07920629871695E-13
Aromatic amino acid metabolism	tyrosine degradation tyrosine homogentisate	Pocket.depth	Deep	-1.78490571717644	0.24679062727772	485	9.42168828828E-13	1.566202728441E-12
Pyrimidine metabolism	uridine monophosphate biosynthesis glutamine prpp ump	Pocket.depth	Deep	0.13158944418556	0.01874572814164	1002	4.09873584029E-12	6.72613060970982E-12
Purine metabolism	guanine ribonucleotide biosynthesis imp gdp gtp	Pocket.depth	Deep	0.068353918355278	0.00992407518982	1002	1.00114844107E-11	1.62211392983466E-11
Cysteine and methionine metabolism	methionine salvage pathway	Pocket.depth	Deep	1.19633811781348	0.17858753575979	925	3.49940255715E-11	5.59904409144408E-11
Central carbohydrate metabolism	citrate cycle first carbon oxidation oxaloacetate 2 oxoglutarate	Pocket.depth	Deep	-0.35399096080723	0.05315707053017	1002	4.53080540055E-11	7.15979125024794E-11
Other carbohydrate metabolism	glucuronate pathway uronate pathway	Pocket.depth	Deep	1.51048910396774	0.22722130581932	860	4.88880271626E-11	7.63130180099185E-11
Other amino acid metabolism	gaba gamma aminobutyrate shunt	Pocket.depth	Deep	-1.89716458402309	0.28594145046091	326	5.3147737673E-11	8.19627761704679E-11
Aromatics degradation	anthranilate degradation anthranilate catechol	Pocket.depth	Deep	-2.05389984836426	0.31732289065039	697	1.50669641227E-10	2.29591834250676E-10
Lysine metabolism	lysine biosynthesis dap aminotransferase pathway aspartate lysine	Pocket.depth	Deep	-0.11444869403554	0.0177140883447	1002	1.62289135985E-10	2.44388345954411E-10
Arginine and proline metabolism	ornithine biosynthesis glutamate ornithine	Pocket.depth	Deep	-0.31585526463068	0.04911967993684	1001	1.96936427175E-10	2.93114682307312E-10
Cofactor and vitamin biosynthesis	ubiquinone biosynthesis prokaryotes chorismate ubiquinone	Pocket.depth	Deep	-1.31024468728111	0.20509903227018	953	2.56534400426E-10	3.77429922465695E-10
Other carbohydrate metabolism	d glucuronate degradation d glucuronate pyruvate d glyceraldehyde 3p	Pocket.depth	Deep	0.662321093080949	0.10407813645786	997	2.99436499589E-10	4.35543999402349E-10
Lysine metabolism	lysine biosynthesis acetyl dap pathway aspartate lysine	Pocket.depth	Deep	-0.32973096677382	0.05198403077139	1002	3.40935728752E-10	4.90334531238272E-10
Fatty acid metabolism	beta oxidation acyl coa synthesis	Pocket.depth	Deep	0.085532257335383	0.01357772582842	1002	4.46862037091E-10	6.35537119418303E-10
Aromatic amino acid metabolism	trans cinnamate degradation trans cinnamate acetyl coa	Pocket.depth	Deep	-1.72670150849848	0.27746600024609	308	7.15802710733E-10	1.00684337333862E-09
Cysteine and methionine metabolism	methionine degradation	Pocket.depth	Deep	1.19175220701355	0.1981313583989	882	2.52067823953E-09	3.50703059412434E-09
Cofactor and vitamin biosynthesis	glutathione biosynthesis glutamate glutathione	Pocket.depth	Deep	-1.00611025731525	0.17065296970843	980	5.09911938325E-09	7.01814280704863E-09
Polyamine biosynthesis	polyamine biosynthesis arginine agmatine putrescine spermidine	Pocket.depth	Deep	0.654107788851703	0.11520408931047	989	1.78715861214E-08	2.43357768461197E-08
Lipopolysaccharide metabolism	adp l glycero d manno heptose biosynthesis	Pocket.depth	Deep	0.496338672679097	0.08870489061901	996	2.84137948311E-08	3.82838498776719E-08
Polyamine biosynthesis	gaba biosynthesis prokaryotes putrescine gaba	Pocket.depth	Deep	-0.86247587702628	0.15691864363221	108	4.9215935463E-08	6.56212472840345E-08
Lysine metabolism	lysine biosynthesis dap dehydrogenase pathway aspartate lysine	Pocket.depth	Deep	-0.06148709948592	0.01155605364826	1002	1.27530181466E-07	1.68287249769875E-07
Branched-chain amino acid metabolism	leucine biosynthesis 2 oxoisovalerate 2 oxoisocaproate	Pocket.depth	Deep	-0.18275772662999	0.03478011580505	1002	1.81236772827E-07	2.36717417569991E-07
Aromatics degradation	catechol meta cleavage catechol acetyl coa 4 methylcatechol propanoyl coa	Pocket.depth	Deep	-0.80878201462972	0.15934126062869	107	4.60010252172E-07	5.94760730081445E-07
Cofactor and vitamin biosynthesis	coenzyme a biosynthesis pantothenate coa	Pocket.depth	Deep	0.057190369212659	0.01253219170278	1002	5.65619898786E-06	7.23993470446163E-06
Carbon fixation	phosphate acetyltransferase acetate kinase pathway acetyl coa acetate	Pocket.depth	Deep	0.054957222848413	0.01306549212637	1002	2.82897967104E-05	3.58524156329263E-05
Aromatic amino acid metabolism	homoprotocatechuate degradation homoprotocatechuate 2 oxohept 3 enedioate	Pocket.depth	Deep	-0.66041961288817	0.15810127564917	108	3.2090047085E-05	4.02698630085757E-05
Other carbohydrate metabolism	dtdp l rhamnose biosynthesis	Pocket.depth	Deep	0.058096864666538	0.0147145905747	1002	8.42390718293E-05	0.000104685448487
Cofactor and vitamin biosynthesis	pantothenate biosynthesis valine l aspartate pantothenate	Pocket.depth	Deep	0.226149024208169	0.06136910240989	1000	0.000240917027832	0.000296513265024
Carbon fixation	reductive citrate cycle arnon buchanan cycle	Pocket.depth	Deep	0.268659568953689	0.08348330921916	1000	0.001331808581771	0.001623538080635
Arginine and proline metabolism	urea cycle	Pocket.depth	Deep	0.29478481174267	0.09497693424985	998	0.001964534369733	0.002372267918169
Aromatic amino acid metabolism	tryptophan metabolism tryptophan kynurenine 2 aminomuconate	Pocket.depth	Deep	-0.61454011715436	0.20369111775136	161	0.002617332716459	0.003131014838381
Cofactor and vitamin biosynthesis	tetrahydrofolate biosynthesis gtp thf	Pocket.depth	Deep	0.108352353068769	0.03663687873786	1001	0.003174725030056	0.003762637072659
Terpenoid backbone biosynthesis	c5 isoprenoid biosynthesis mevalonate pathway	Pocket.depth	Deep	-0.36630552481078	0.12852762310016	978	0.004461520437397	0.005239216660429
Cofactor and vitamin biosynthesis	c1 unit interconversion prokaryotes	Pocket.depth	Deep	0.063838848645943	0.02351201822271	1001	0.006738532914549	0.00784120193693
Cysteine and methionine metabolism	cysteine biosynthesis methionine cysteine	Pocket.depth	Deep	0.495060779388507	0.18829202744177	939	0.008689476066046	0.01002029672481
Central carbohydrate metabolism	citrate cycle second carbon oxidation 2 oxoglutarate oxaloacetate	Pocket.depth	Deep	-0.04092099870062	0.01653897386812	1002	0.013517918167307	0.015449049334066

Other carbohydrate metabolism	galactose degradation leloir pathway galactose alpha d glucose 1p	Pocket.depth	Deep	0.064201629260563	0.02599177084148	1002	0.013674436481156	0.015489627164495
Serine and threonine metabolism	serine biosynthesis glycerate 3p serine	Pocket.depth	Deep	-0.1156978994022	0.05004152757881	1002	0.020978012318001	0.023349439797427
Cysteine and methionine metabolism	cysteine biosynthesis homocysteine serine cysteine	Pocket.depth	Deep	-0.47552865730215	0.20545985697043	940	0.020844338606057	0.023349439797427
Cofactor and vitamin biosynthesis	ascorbate degradation ascorbate d xylulose 5p	Pocket.depth	Deep	-0.25069028138585	0.11231892765377	994	0.02583911519321	0.028512127109749
Methane metabolism	formaldehyde assimilation xylulose monophosphate pathway	Pocket.depth	Deep	-0.35598495040857	0.16451405742285	123	0.030711507557519	0.033598914250961
Cysteine and methionine metabolism	methionine biosynthesis apartate homoserine methionine	Pocket.depth	Deep	-0.08568212760622	0.04127838987867	1002	0.038175524595702	0.04141073854449
Other carbohydrate metabolism	propanoyl coa metabolism propanoyl coa succinyl coa	Pocket.depth	Deep	-0.42929972128147	0.21179803013192	944	0.042934514501804	0.046181662657402
Purine metabolism	inosine monophosphate biosynthesis prpp glutamine imp	Pocket.depth	Deep	-0.026293097383	0.0134413047778	1002	0.050726533917699	0.054108302845546
Carbon fixation	reductive pentose phosphate cycle ribulose 5p glyceraldehyde 3p	Pocket.depth	Deep	0.308968107198122	0.16790757721689	971	0.066048564962754	0.069869556324235
Carbon fixation	reductive pentose phosphate cycle calvin cycle	Pocket.depth	Deep	0.066517586776537	0.03732766819872	1000	0.075053990417789	0.078745170274402
Other carbohydrate metabolism	undecaprenylphosphate alpha l ara4n biosynthesis udp glca undecaprenyl phosphate alp	Pocket.depth	Deep	-0.33429888419724	0.21778120815407	145	0.125094804520181	0.130179959175473
Methane metabolism	formaldehyde assimilation ribulose monophosphate pathway	Pocket.depth	Deep	0.207143151992621	0.13575752774069	987	0.127367957081868	0.131476600858702
Aromatic amino acid metabolism	tyrosine biosynthesis chorismate tyrosine	Pocket.depth	Deep	0.09081453137396	0.07469471735674	999	0.224344510131576	0.229728778374734

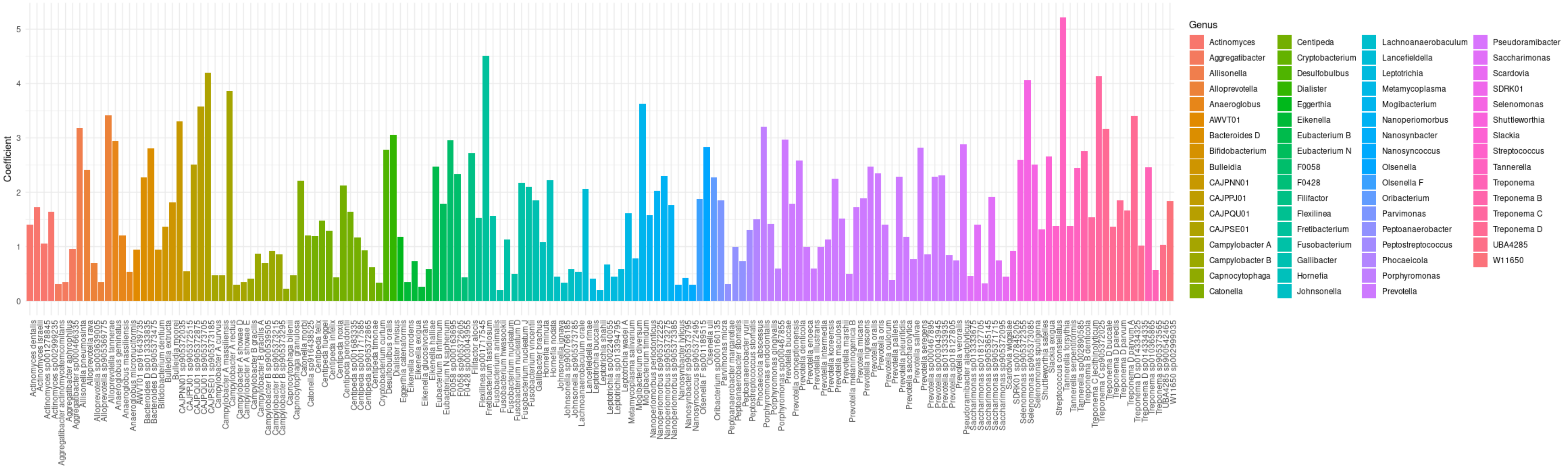

Supplementary Figure 2. Top significant species that positively associated with deep pocket (negatively associated with shallow pocket) in individuals with both shallow and deep pocket in the oral cavity. The analysis was performed using MaAsLin2

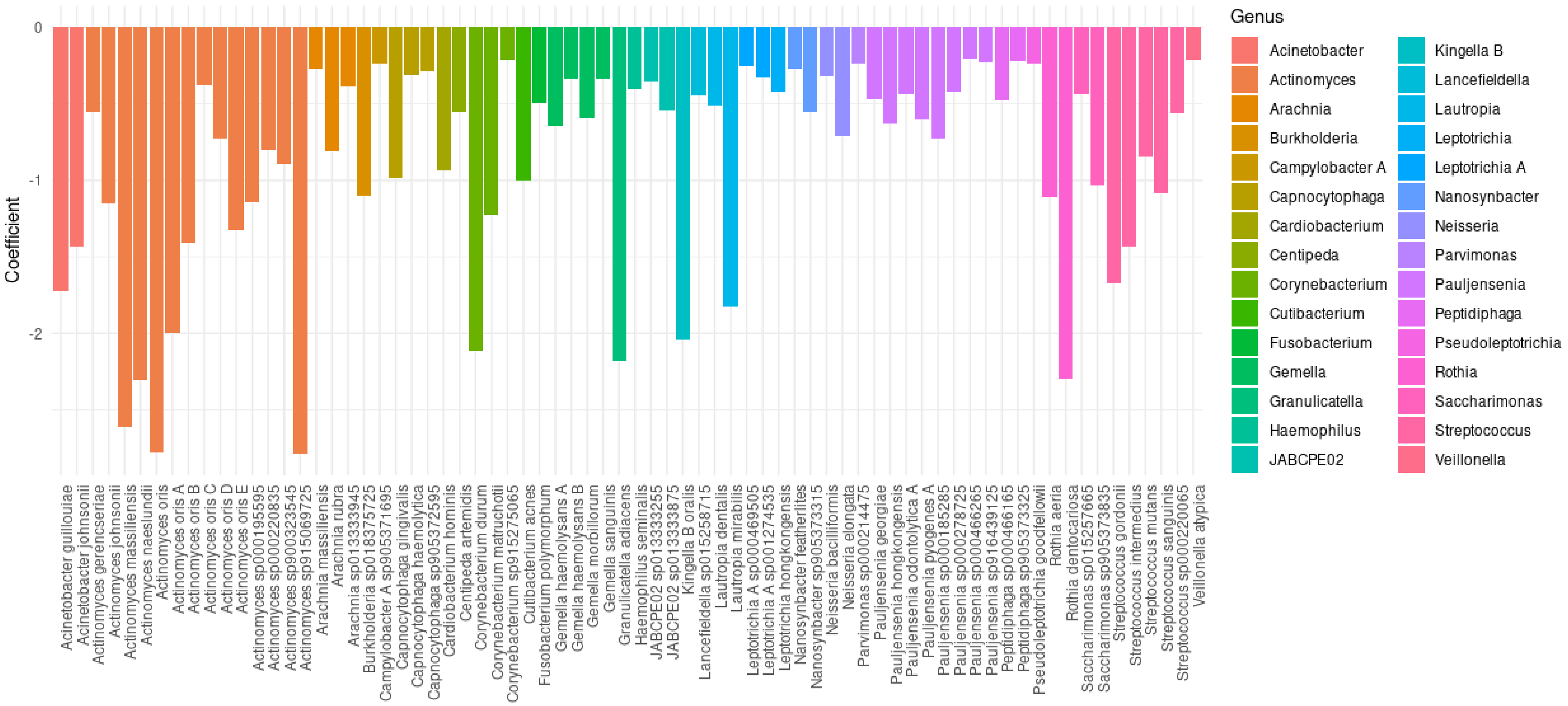

Supplementary Figure 3. Top significant species that negatively associated with deep pocket (positively associated with shallow pocket) in individuals with both shallow and deep pocket in the oral cavity. The analysis was performed using MaAsLin2

Supplementary Table 4. Demographic and clinical metadata of participants, including periodontal parameters and clinical examination findings

SampleID	Sex	Periodental.pocket	Mean.GR	Mean.CAL	Mean.PPD	Proportion.BoP	Periodontitis.stage	Periodontitis.severity
FR22703531	Female	Shallow	0.697986577	2.767741935	2.096774194	0.08974359	II	Mild/Moderate
FR22703532	Male	Shallow	0.554545455	2.765151515	2.303030303	0.401515152	III	Mild/Moderate
FR22703538	Male	Shallow	0.554545455	2.765151515	2.303030303	0.401515152	II	Mild/Moderate
FR22703539	Male	Shallow	0.425742574	1.693333333	1.406666667	0.14	III	Mild/Moderate
FR22703547	Male	Deep	1.105263158	3.346666667	2.366666667	0.34	III	Mild/Moderate
FR22703548	Female	Deep	0.649122807	2.256410256	2.019230769	0.519230769	IV	Severe
FR22703555	Female	Shallow	0.126582278	2.683333333	2.6	0.341666667	III	Mild/Moderate
FR22703563	Female	Shallow	0.06	1.953333333	1.893333333	0.3	II	Mild/Moderate
FR22703573	Male	Deep	0.428571429	2.491017964	2.221556886	0.107142857	III	Mild/Moderate
FR22703581	Male	Shallow	0.117241379	2.6	2.486666667	0.68	III	Mild/Moderate
FR22703584	Male	Shallow	0.100840336	2.777777778	2.703703704	0.265432099	III	Mild/Moderate
FR22703590	Male	Shallow	0.858490566	3.515873016	2.810606061	0.393939394	III	Mild/Moderate
FR22703595	Female	Deep	0.694444444	3.393333333	2.726666667	0.686666667	III	Mild/Moderate
FR22703597	Male	Deep	0.117241379	2.6	2.486666667	0.68	III	Mild/Moderate
FR22703599	Female	Shallow	0.73	2.461538462	2.041958042	0.12	IV	Severe
FR22703608	Female	Shallow	0.185185185	2.127272727	1.981818182	0.196428571	III	Mild/Moderate
FR22703610	Male	Deep	0.611510791	3.342105263	2.782894737	0.461538462	IV	Severe
FR22703623	Male	Shallow	0.713235294	3.454545455	2.710144928	0.68115942	III	Mild/Moderate
FR22703624	Male	Shallow	0.221311475	2.544871795	2.371794872	0.333333333	III	Mild/Moderate
FR22703627	Female	Shallow	0.098765432	2.141975309	2.043209877	0.302469136	III	Mild/Moderate
FR22703629	Male	Deep	0.098765432	2.141975309	2.043209877	0.302469136	IV	Severe
FR22703635	Male	Shallow	1.255813953	3.934782609	3.152173913	0.949275362	III	Mild/Moderate
FR22703636	Male	Shallow	0.094202899	2.833333333	2.75	0.538461538	III	Severe
FR22703638	Male	Shallow	0.639097744	3.038461538	2.493589744	0.493589744	III	Severe
FR22703641	Female	Shallow	0.178082192	2.761904762	2.607142857	0.291666667	III	Mild/Moderate
FR22703647	Female	Shallow	0.470238095	3.31547619	2.845238095	0.666666667	III	Mild/Moderate
FR22703659	Female	Deep	0.465277778	2.679012346	2.265432099	0.660493827	III	Mild/Moderate
FR22703661	Female	Shallow	0.204545455	2.597222222	2.409722222	0.527777778	I	No periodontitis
FR22703662	Female	Shallow	0.762237762	2.881118881	2.118881119	0.6875	III	Severe
FR22703664	Male	Shallow	0.235714286	2.972222222	2.743055556	0.465277778	III	Mild/Moderate
FR22703668	Male	Deep	0.583333333	3.290123457	2.771604938	0.475308642	III	Mild/Moderate
FR22703678	Female	Shallow	1.88372093	4.522727273	2.681818182	0.704545455	IV	Severe
FR22703679	Female	Shallow	0.206451613	3.255952381	3.06547619	0.785714286	III	Mild/Moderate
FR22703684	Male	Shallow	1.433962264	2.953333333	2.446666667	0.553333333	III	Mild/Moderate
FR22703705	Male	Deep	0.406666667	3.285714286	2.922619048	0.636904762	III	Mild/Moderate
FR22703713	Female	Deep	0.776315789	3.130952381	2.428571429	0.779761905	IV	Severe

FR22703719	Female	Deep	0.06	1.953333333	1.893333333	0.3	III	Severe
FR22703723	Female	Deep	1.851851852	3.524193548	3.120967742	0.833333333	III	Mild/Moderate
FR22703724	Male	Deep	0.610526316	2.868055556	2.465277778	0.326388889	IV	Severe
FR22703725	Female	Shallow	0.536585366	2.725663717	2.415929204	0.225	II	Mild/Moderate
FR22703726	Male	Deep	1.151515152	3.227272727	2.651515152	0.239130435	IV	Severe
FR22703727	Male	Shallow	1.151515152	3.227272727	2.651515152	0.239130435	IV	Severe
FR22703728	Male	Deep	0.299270073	2.12345679	1.87037037	0.160493827	III	Mild/Moderate
FR22703729	Male	Shallow	0.610526316	2.868055556	2.465277778	0.326388889	III	Mild/Moderate
FR22703730	Female	Shallow	0.298076923	3.049382716	2.858024691	0.759259259	III	Mild/Moderate
FR22703731	Male	Shallow	0.827160494	3.291666667	2.494047619	0.339285714	IV	Severe
FR22703732	Male	Deep	0.48447205	3.055555556	2.574074074	0.592592593	III	Severe
FR22703733	Female	Deep	0.380952381	2.3	2.086666667	0.26	III	Severe
FR22703735	Female	Shallow	0.852941176	2.66025641	1.916666667	0.179487179	III	Mild/Moderate
FR22703736	Female	Deep	NA	NA	NA	NA	III	Mild/Moderate
FR22703737	Male	Shallow	2.305263158	4.144927536	2.557971014	0.152173913	IV	Severe
FR22703738	Male	Deep	2.305263158	4.144927536	2.557971014	0.152173913	IV	Severe
FR22703739	Male	Shallow	0.865384615	4.138888889	3.722222222	0.574074074	III	Severe
FR22703740	Male	Deep	0.565656566	2.160839161	1.769230769	0.270833333	III	Mild/Moderate
FR22703741	Male	Deep	0.653846154	3.458333333	2.851190476	0.678571429	III	Severe
FR22703743	Female	Shallow	0.328671329	2.743055556	2.416666667	0.277777778	III	Mild/Moderate
FR22703744	Female	Shallow	0.30952381	3.304347826	3.02173913	0.52173913	III	Mild/Moderate
FR22703745	Male	Shallow	0.4875	2.8875	2.425	0.273809524	II	Mild/Moderate
FR22703746	Male	Deep	0.404761905	2.19047619	1.886904762	0.333333333	III	Severe
FR22703747	Male	Shallow	2.307017544	3.782051282	2.096153846	0.076923077	III	Mild/Moderate
FR22703748	Male	Shallow	1.125	3.571428571	2.607142857	0.630952381	IV	Severe
FR22703749	Male	Deep	0.39869281	3.025641026	2.634615385	0.384615385	III	Mild/Moderate
FR22703750	Male	Shallow	0.571428571	4.853333333	4.293333333	0.973333333	III	Severe
FR22703752	Female	Deep	0.43030303	2.074534161	1.670807453	0.089285714	III	Mild/Moderate
FR22703754	Male	Shallow	0.378378378	2.653846154	2.474358974	0.378205128	III	Severe
FR22703755	Male	Deep	2.643939394	4.765151515	2.121212121	0.189393939	III	Severe
FR22703756	Female	Shallow	1.134146341	3.955128205	2.804878049	0.666666667	III	Mild/Moderate
FR22703757	Male	Shallow	0.299319728	3.24691358	2.975308642	0.595238095	III	Mild/Moderate
FR22703760	Female	Deep	0.191489362	1.789808917	1.617834395	0.029761905	III	Mild/Moderate
FR22703761	Female	Shallow	0.147540984	2.569444444	2.506944444	0.847222222	III	Mild/Moderate
FR22703762	Male	Shallow	0.378378378	2.653846154	2.474358974	0.378205128	III	Mild/Moderate
FR22703763	Female	Shallow	0.48447205	3.055555556	2.574074074	0.592592593	II	Mild/Moderate
FR22703764	Female	Shallow	0.329192547	2.851851852	2.524691358	0.518518519	I	No periodontitis
FR22703765	Male	Shallow	0.468253968	3.234848485	2.862318841	0.492753623	III	Mild/Moderate

FR22703766	Male	Deep	0.468253968	3.234848485	2.862318841	0.492753623	III	Mild/Moderate
FR22703767	Female	Shallow	0.761904762	3.3625	2.66875	0.209876543	II	Mild/Moderate
FR22703769	Female	Shallow	1.185185185	3.616766467	2.664670659	0.446428571	III	Severe
FR22703770	Male	Shallow	0.549180328	3.5	3.08125	0.734567901	NA	Mild
FR22703771	Male	Deep	0.879032258	2.971631206	2.255319149	0.375	III	Mild/Moderate
FR22703772	Male	Shallow	0.879032258	2.971631206	2.255319149	0.375	III	Mild/Moderate
FR22703773	Male	Deep	0.865384615	4.138888889	3.722222222	0.574074074	III	Mild/Moderate
FR22703774	Male	Shallow	0.761904762	3.3625	2.66875	0.209876543	III	Mild/Moderate
FR22703775	Male	Deep	0.469879518	3.035714286	2.571428571	0.303571429	III	Severe
FR22703776	Male	Shallow	0.329192547	2.851851852	2.524691358	0.518518519	III	Severe
FR22703777	Male	Deep	0.536423841	2.767741935	2.24516129	0.493589744	III	Mild/Moderate
FR22703778	Female	Deep	1.468085106	3.269230769	1.942307692	0.147435897	III	Mild/Moderate
FR22703779	NA	Shallow	0.630952381	3.303571429	2.672619048	0.660714286	NA	NA
FR22703780	Female	Shallow	0.975	3.808333333	2.833333333	0.283333333	III	Severe
FR22703781	Female	Shallow	1.051948052	3.288461538	2.25	0.423076923	II	Mild/Moderate
FR22703782	Female	Shallow	1.051948052	3.288461538	2.25	0.423076923	III	Mild/Moderate
FR22703783	Female	Shallow	1.166666667	4.266666667	3.1	0.633333333	III	Mild/Moderate
FR22703784	Male	Deep	1.351851852	3.962962963	2.611111111	0.697530864	IV	Severe
FR22703786	Female	Shallow	0.829787234	2.722222222	2.240740741	0.697530864	III	Mild/Moderate
FR22703787	Male	Deep	1.166666667	4.266666667	3.1	0.633333333	IV	Severe
FR22703788	Male	Shallow	0.630952381	3.303571429	2.672619048	0.660714286	IV	Severe
FR22703789	NA	Deep	0.928571429	3.421052632	2.50877193	0.657894737	NA	NA
FR22703790	Female	Deep	0.564102564	3.788461538	3.224358974	0.487179487	IV	Severe
FR22703791	Female	Deep	0.975	3.808333333	2.833333333	0.283333333	III	Severe
FR22703792	Female	Shallow	0.564102564	3.788461538	3.224358974	0.487179487	IV	Severe
FR22703793	Male	Shallow	0.549180328	3.5	3.08125	0.734567901	III	Severe
FR22703794	Female	Shallow	0.333333333	2.416666667	2.314102564	0.666666667	III	Mild/Moderate
FR22703795	Female	Shallow	0.391304348	3.512820513	3.166666667	0.419753086	III	Mild/Moderate
FR22703796	Female	Deep	1.510791367	3.261904762	2.011904762	0.160714286	III	Mild/Moderate
FR22703797	Male	Shallow	0.481481481	3.117283951	2.635802469	0.5	III	Severe
FR22703798	Male	Shallow	1.510791367	3.261904762	2.011904762	0.160714286	III	Mild/Moderate
FR22703799	Male	Deep	0.28440367	2.333333333	2.126666667	0.466666667	III	Mild/Moderate
FR22703800	Male	Deep	0.845238095	2.525641026	2.070512821	0.275641026	III	Severe
FR22703801	Male	Deep	0.777777778	2.537037037	1.975308642	0.117283951	NA	Mild
FR22703802	Female	Deep	0.333333333	2.416666667	2.314102564	0.666666667	III	Mild/Moderate
FR22703804	Male	Shallow	2.307017544	3.782051282	2.096153846	0.076923077	III	Mild/Moderate
FR22703805	Female	Deep	0.535087719	2.310606061	1.848484848	0.045454545	III	Mild/Moderate
FR22703806	Female	Shallow	0.698113208	2.553571429	2.113095238	0.386904762	III	Mild/Moderate

FR22703808	Male	Shallow	1.628318584	3.066666667	1.84	0.053333333	III	Mild/Moderate
FR22703809	Male	Deep	0.160714286	2.452380952	2.345238095	0.31547619	IV	Severe
FR22703811	Male	Deep	1.168067227	2.861111111	1.895833333	0.069444444	IV	Mild/Moderate
FR22703812	Male	Shallow	1.468085106	3.269230769	1.942307692	0.147435897	IV	Mild/Moderate
FR22703813	Female	Deep	0.829787234	2.722222222	2.240740741	0.697530864	III	Mild/Moderate
FR22703814	Female	Shallow	0.160714286	2.452380952	2.345238095	0.31547619	III	Mild/Moderate
FR22703815	Male	Shallow	0.616666667	2.49122807	2.166666667	0.51754386	IV	Severe
FR22703816	Male	Shallow	1.430107527	3.068181818	2.060606061	0.159090909	III	Mild/Moderate
FR22703817	Male	Deep	0.120567376	1.786666667	1.673333333	0.406666667	III	Severe
FR22703818	Female	Shallow	1.469135802	3.808641975	2.339506173	0.172839506	III	Mild/Moderate
FR22703819	Female	Shallow	1.083333333	2.7	2.158333333	0.25	III	Mild/Moderate
FR22703820	Female	Deep	0.251612903	2.845238095	2.613095238	0.44047619	III	Mild/Moderate
FR22703821	Female	Shallow	0.6015625	2.869565217	2.311594203	0.420289855	III	Mild/Moderate
FR22703822	Female	Shallow	0.829268293	2.475	2.166666667	0.166666667	III	Mild/Moderate
FR22703823	Male	Shallow	0.266666667	2.072072072	1.853658537	0.23015873	III	Mild/Moderate
FR22703824	Male	Deep	0.338582677	3.270833333	2.973333333	0.553333333	III	Mild/Moderate
FR22703825	Male	Shallow	0.462962963	2.242038217	1.78343949	0.055555556	III	Mild/Moderate
FR22703826	Male	Shallow	0.4	3	2.666666667	0.558333333	IV	Severe
FR22703827	Female	Deep	0.207142857	3.023952096	2.850299401	0.702380952	III	Mild/Moderate
FR22703828	Female	Shallow	NA	NA	NA	NA	III	Mild/Moderate
FR22703834	Male	Deep	0.987421384	3.395061728	2.425925926	0.407407407	III	Mild/Moderate
FR22703835	Female	Shallow	2.535714286	4.833333333	2.579365079	0.30952381	I	No periodontitis
FR22703850	Male	Shallow	0.375886525	2.888888889	2.520833333	0.631944444	II	Mild/Moderate
FR22703851	Male	Shallow	0.41322314	2.811594203	2.54	0.373333333	III	Mild/Moderate
FR22703855	Female	Deep	0.146153846	2.564102564	2.442307692	0.685897436	III	Mild/Moderate
FR22703856	Female	Shallow	0.438356164	3.153333333	2.726666667	0.36	III	Mild/Moderate
FR22703857	Female	Shallow	0.216666667	2.679012346	2.518518519	0.796296296	III	Mild/Moderate
FR22703858	Female	Shallow	0.20952381	3.347222222	3.194444444	0.756944444	II	Mild/Moderate
FR22703859	Female	Deep	0.571428571	3.576388889	3.076388889	0.576388889	III	Mild/Moderate
FR22703860	Female	Shallow	0.381355932	2.971014493	2.644927536	0.513888889	III	Mild/Moderate
FR22703861	Male	Deep	1.954887218	3.147435897	1.480769231	0.108974359	III	Mild/Moderate
FR22703862	Male	Shallow	0.560747664	2.592592593	2.222222222	0.24691358	III	Mild/Moderate
FR22703863	Male	Shallow	0.173228346	2.854166667	2.701388889	0.458333333	IV	Severe
FR22703864	Male	Deep	0.173228346	2.854166667	2.701388889	0.458333333	IV	Severe
FR22703866	Female	Shallow	0.094202899	2.833333333	2.75	0.538461538	IV	Severe
FR22703867	Female	Deep	0.291044776	3.286666667	3.025641026	0.826923077	IV	Severe
FR22703869	Male	Shallow	0.242105263	3.121212121	2.946969697	0.727272727	III	Mild/Moderate
FR22703870	Male	Shallow	0.349056604	3.16	2.913333333	0.686666667	III	Mild/Moderate

FR22703873	Female	Deep	0.510869565	3.431818182	3.075757576	0.537878788	IV	Severe
FR22703874	Female	Shallow	0.510869565	3.431818182	3.075757576	0.537878788	IV	Severe
FR22703876	Male	Deep	0.221518987	2.577380952	2.369047619	0.470238095	III	Mild/Moderate
FR22703877	Female	Shallow	1.101010101	3.083333333	2.261904762	0.261904762	III	Mild/Moderate
FR22703884	Female	Deep	0.496503497	2.979166667	2.486111111	0.326388889	III	Mild/Moderate
FR22703886	Male	Shallow	0.865771812	2.839285714	2.071428571	0.154761905	III	Severe
FR22703888	Male	Deep	0.292682927	2.648809524	2.363095238	0.535714286	III	Mild/Moderate
FR22703889	Male	Shallow	0.386904762	3.172619048	2.785714286	0.535714286	III	Mild/Moderate
FR22703890	Male	Deep	0.074829932	2.81547619	2.75	0.380952381	III	Mild/Moderate
FR22703891	Female	Shallow	0.744	3.047619048	2.494047619	0.505952381	III	Mild/Moderate
FR22703892	Male	Deep	1.824	3.75	2.288461538	0.320512821	III	Mild/Moderate
FR22703894	Female	Shallow	0.660377358	3.251497006	2.622754491	0.404761905	III	Mild/Moderate
FR22703895	Male	Shallow	2.2	3.385416667	2.239583333	0.3125	III	Mild/Moderate
FR22703896	Female	Deep	0.987654321	3.06547619	2.113095238	0.327380952	III	Mild/Moderate
FR22703897	Male	Shallow	0.857142857	2.348484848	1.97826087	0.038461538	II	Mild/Moderate
FR22703899	Male	Deep	0.69	3.144927536	2.644927536	0.739130435	IV	Mild/Moderate
FR22703900	Male	Shallow	0.616	2.592592593	2.117283951	0.172839506	II	Mild/Moderate
FR22703901	Male	Shallow	0.642857143	2.375	2.053571429	0.31547619	II	Mild/Moderate
FR22703902	Male	Shallow	0.72519084	2.589285714	2.023809524	0.119047619	III	Mild/Moderate
FR22703903	Male	Shallow	0.126582278	2.683333333	2.6	0.341666667	III	Mild/Moderate
FR22703905	Male	Deep	0.104477612	2.913580247	2.827160494	0.814814815	III	Mild/Moderate
FR22703907	Male	Shallow	1.208333333	3.910714286	2.702380952	0.339285714	IV	Mild/Moderate
FR22703908	Male	Shallow	0.635658915	3.527777778	2.958333333	0.409722222	II	Mild/Moderate
FR22703909	Female	Shallow	1.105263158	3.346666667	2.366666667	0.34	III	Mild/Moderate
FR22703910	Male	Deep	1.538461538	3.308641975	2.320987654	0.296296296	III	Mild/Moderate
FR22703911	Female	Shallow	0.364285714	2.551282051	2.224358974	0.653846154	III	Mild/Moderate
FR22703912	Male	Shallow	0.74789916	3.121794872	2.551282051	0.602564103	III	Mild/Moderate
FR22703913	Female	Deep	0.086666667	1.94	1.853333333	0.253333333	III	Mild/Moderate
FR22703914	Male	Shallow	0.298076923	3.049382716	2.858024691	0.759259259	II	No periodontitis
FR22704107	Male	Deep	0.185185185	2.012345679	1.827160494	0.12962963	III	Severe
FR22704108	Male	Shallow	0.669230769	3.096153846	2.538461538	0.326923077	III	Mild/Moderate
FR22704109	Male	Shallow	0.527272727	2.869047619	2.351190476	0.166666667	III	Mild/Moderate
FR22704110	Male	Deep	0.462962963	2.242038217	1.78343949	0.055555556	III	Severe
FR22704111	Male	Shallow	0.190140845	2.488095238	2.327380952	0.523809524	III	Severe
FR22704112	Female	Shallow	0.58974359	2.123376623	1.538961039	0.198717949	III	Mild/Moderate
FR22704113	Male	Shallow	0.307692308	2.767857143	2.625	0.386904762	IV	Severe
FR22704114	Female	Deep	0.077380952	1.608695652	1.571428571	0.107142857	III	Mild/Moderate
FR22704115	Female	Deep	0.765060241	3.56547619	2.80952381	0.410714286	III	Severe

FR22704116	Male	Deep	0.469798658	3.051282051	2.602564103	0.275641026	IV	Severe
FR22704117	Male	Shallow	0.817460317	3.14	2.506410256	0.532051282	IV	Severe
FR22704118	Male	Shallow	0.1	1.272058824	1.161764706	0.026666667	IV	Severe
FR22704119	Male	Deep	0.817460317	3.14	2.506410256	0.532051282	IV	Severe
FR22704120	Female	Deep	0.22556391	3.006410256	2.814102564	0.448717949	III	Severe
FR22704121	Female	Shallow	0.623376623	2.821428571	2.25	0.386904762	III	Severe
FR22704122	Male	Deep	0.307692308	2.767857143	2.625	0.386904762	IV	Severe
FR22704123	Female	Shallow	0.469798658	3.051282051	2.602564103	0.275641026	III	Severe
FR22704124	Female	Shallow	0.351190476	2.94047619	2.589285714	0.571428571	IV	Severe
FR22704125	Female	Deep	0.2	3.011904762	2.880952381	0.654761905	IV	Severe
FR22704126	Male	Deep	0.3	2.541666667	2.255952381	0.380952381	IV	Severe
FR22704127	Male	Shallow	0.528735632	2.603174603	2.238095238	0.277777778	IV	Severe
FR22704128	Female	Shallow	0.795081967	3.493589744	2.871794872	0.525641026	III	Mild/Moderate
FR22704129	Female	Deep	0.544642857	3.121794872	2.730769231	0.66025641	III	Mild/Moderate
FR22704130	Female	Shallow	0.795081967	3.493589744	2.871794872	0.525641026	III	Mild/Moderate
FR22704131	Female	Deep	0.1	1.272058824	1.161764706	0.026666667	III	Severe
FR22704132	Female	Shallow	0.871212121	3.608695652	2.775362319	0.492753623	III	Severe
FR22704133	Male	Shallow	0.427419355	3.240740741	2.913580247	0.672839506	III	Severe
FR22704134	Male	Deep	0.427419355	3.240740741	2.913580247	0.672839506	IV	Severe
FR22704135	Male	Shallow	0.871212121	3.608695652	2.775362319	0.492753623	IV	Severe
FR22704136	Female	Shallow	0.544642857	3.121794872	2.730769231	0.66025641	III	Mild/Moderate
FR22704137	Female	Deep	NA	NA	NA	NA	III	Mild/Moderate
FR22704138	Female	Shallow	0.2	3.011904762	2.880952381	0.654761905	III	Mild/Moderate
FR22704139	Female	Deep	0.225352113	2.892857143	2.702380952	0.541666667	IV	Severe
FR22704141	Female	Shallow	0.117283951	2.888888889	2.771604938	0.456790123	IV	Severe
FR22704144	Male	Deep	0.148809524	2.21686747	2.06626506	0.160714286	III	Severe
FR22704146	Male	Deep	0.207142857	3.023952096	2.850299401	0.702380952	IV	Severe
FR22704147	Male	Deep	0.542056075	3.042735043	2.788617886	0.341269841	III	Severe
FR22704148	Female	Shallow	0.225352113	2.892857143	2.702380952	0.541666667	III	Mild/Moderate
FR22704149	Female	Deep	1.851851852	3.524193548	3.120967742	0.833333333	III	Mild/Moderate
FR22704150	Female	Deep	0.676190476	2.666666667	2.140350877	0.116666667	III	Mild/Moderate
FR22704151	Female	Shallow	0.475728155	2.33974359	2.025641026	0.205128205	III	Mild/Moderate
FR22704152	Male	Shallow	1.072164948	3.359649123	2.447368421	0.526315789	IV	Severe
FR22704153	Male	Deep	1.14516129	2.611111111	2.172839506	0.395061728	III	Mild/Moderate
FR22704154	Female	Shallow	0.610619469	3.122302158	2.625899281	0.708333333	III	Mild/Moderate
FR22704155	Male	Shallow	0.349206349	2.196428571	1.87804878	0.113636364	II	Mild/Moderate
FR22704156	Male	Shallow	0.74789916	3.121794872	2.551282051	0.602564103	III	Severe
FR22704157	Female	Shallow	0.496296296	2.8125	2.347222222	0.284722222	III	Mild/Moderate

FR22704159	Female	Shallow	0.428571429	2.491017964	2.221556886	0.107142857	III	Mild/Moderate
FR22704160	Female	Shallow	1.458015267	3.530120482	2.379518072	0.202380952	III	Mild/Moderate
FR22704161	Female	Shallow	1.255813953	3.934782609	3.152173913	0.949275362	II	Mild/Moderate
FR22704163	Female	Shallow	0.470238095	2.144578313	1.698795181	0.101190476	II	No periodontitis
FR22704164	Female	Deep	0.610619469	3.122302158	2.625899281	0.708333333	III	Severe
FR22704165	Female	Shallow	1.583333333	4.224637681	2.985507246	0.528985507	III	Mild/Moderate
FR22704166	Female	Deep	0.190140845	2.488095238	2.327380952	0.523809524	III	Mild/Moderate
FR22704167	Female	Shallow	0.251612903	2.845238095	2.613095238	0.44047619	III	Mild/Moderate
FR22704168	Female	Deep	0.307692308	2.207792208	1.902597403	0.262820513	IV	Severe
FR22704169	Male	Shallow	0.4	3	2.666666667	0.558333333	III	Mild/Moderate
FR22704170	Female	Shallow	0.858156028	3.44	2.633333333	0.42	III	Mild/Moderate
FR22704171	Female	Deep	0.635658915	3.527777778	2.958333333	0.409722222	III	Mild/Moderate
FR22704172	Male	Deep	0.270676692	3.106666667	2.866666667	0.626666667	IV	Severe
FR22704173	Male	Deep	0.270676692	3.106666667	2.866666667	0.626666667	III	Severe
FR22704174	Male	Deep	0.104477612	2.913580247	2.827160494	0.814814815	III	Mild/Moderate
FR22704175	Female	Deep	0.153846154	3.261904762	3.142857143	0.726190476	III	Severe
FR22704176	Male	Deep	0.858156028	3.44	2.633333333	0.42	IV	Severe
FR22704178	Female	Deep	0.69	3.144927536	2.644927536	0.739130435	III	Mild/Moderate
FR22704179	Female	Deep	0.256198347	2.36	2.153333333	0.286666667	III	Mild/Moderate
FR22704181	Female	Deep	0.086666667	1.94	1.853333333	0.253333333	III	Severe
FR22704182	Female	Deep	1.603174603	3.738095238	2.134920635	0.090909091	III	Mild/Moderate
FR22704183	Female	Deep	0.185185185	2.012345679	1.827160494	0.12962963	III	Severe
FR22704184	Female	Deep	1.208333333	3.910714286	2.702380952	0.339285714	III	Severe
FR22704185	Male	Deep	1	3.166666667	2.442307692	0.647435897	III	Severe
FR22704186	Female	Shallow	1.257668712	3.125	1.904761905	0.119047619	III	Mild/Moderate
FR22704188	Female	Shallow	1.603174603	3.738095238	2.134920635	0.090909091	III	Mild/Moderate
FR22704189	Male	Deep	0.472222222	2.839506173	2.419753086	0.43452381	III	Severe
FR22704190	Male	Deep	0.127659574	1.775	1.7	0.333333333	IV	Severe
FR22704191	Male	Deep	0.915254237	2.884057971	2.101449275	0.311594203	III	Mild/Moderate
FR22704193	Male	Deep	0.676190476	2.666666667	2.140350877	0.116666667	III	Mild/Moderate
FR22704194	Male	Deep	0.831683168	2.565217391	1.956521739	0.289855072	III	Severe
FR22704195	Male	Shallow	NA	NA	NA	NA	III	Mild/Moderate
FR22704196	Male	Shallow	1.258064516	2.99382716	2.030864198	0.179012346	IV	Severe
FR22704197	Male	Shallow	0.3125	2.839285714	2.541666667	0.43452381	III	Mild/Moderate
FR22704198	Male	Deep	0.850393701	3.347222222	2.597222222	0.569444444	III	Mild/Moderate
FR22704199	Male	Shallow	0.060810811	2.803571429	2.75	0.541666667	III	Mild/Moderate
FR22704200	Male	Deep	0.071895425	2.346153846	2.275641026	0.121794872	IV	Severe
FR22704201	Male	Deep	0.19047619	3.125	2.958333333	0.283333333	IV	Severe

FR22704202	Female	Deep	0.6015625	2.869565217	2.311594203	0.420289855	IV	Mild/Moderate
FR22704203	Male	Deep	0.470238095	3.55952381	3.089285714	0.482142857	III	Mild/Moderate
FR22704207	Male	Deep	0.338582677	3.270833333	2.973333333	0.553333333	III	Mild/Moderate
FR22704208	Female	Deep	0.765060241	3.56547619	2.80952381	0.410714286	III	Severe
FR22704210	Male	Deep	0.119047619	2.05952381	1.94047619	0.523809524	IV	Severe
FR22704215	Female	Deep	0.117283951	2.038709677	1.95483871	0.12962963	III	Mild/Moderate
FR22704216	Male	Shallow	0.119047619	2.05952381	1.94047619	0.523809524	IV	Severe
FR22704218	Female	Deep	0.313559322	2.788461538	2.551282051	0.820512821	III	Mild/Moderate
FR22704220	Female	Shallow	1.344262295	2.373333333	1.891025641	0.108974359	III	Severe
FR22704222	Female	Shallow	0.261904762	2.163265306	1.945578231	0.2	III	Severe
FR22704224	Female	Deep	0.266666667	3.347222222	3.097222222	0.736111111	III	Mild/Moderate
FR22704225	Female	Shallow	0.583333333	3.290123457	2.771604938	0.475308642	III	Mild/Moderate
FR22704228	Female	Shallow	0.472440945	2.346153846	1.961538462	0.435897436	III	Mild/Moderate
FR22704244	Male	Deep	0.263636364	2.891025641	2.705128205	0.570512821	III	Mild/Moderate
FR22704245	Female	Shallow	0.265432099	2.901234568	2.635802469	0.679012346	III	Mild/Moderate
FR22704247	Female	Shallow	0.185840708	3.397163121	3.24822695	0.701388889	III	Severe
FR22704248	Female	Shallow	0.386792453	3.113475177	2.823129252	0.7	III	Mild/Moderate
FR22704250	Female	Shallow	0.34	2.493589744	2.166666667	0.256410256	III	Mild/Moderate
FR22704251	Male	Shallow	0.117283951	2.038709677	1.95483871	0.12962963	III	Mild/Moderate
FR22704252	Female	Shallow	0.442176871	3.395061728	2.99382716	0.438271605	III	Mild/Moderate
FR22704253	Male	Shallow	0.148809524	2.21686747	2.06626506	0.160714286	IV	Severe
FR22704256	Male	Shallow	0.230769231	2.976190476	2.761904762	0.416666667	III	Mild/Moderate
FR22704257	Male	Deep	1.125	3.571428571	2.607142857	0.630952381	IV	Severe
FR22704259	Female	Shallow	0.360824742	2.492753623	2.239130435	0.108695652	III	Mild/Moderate
FR22704260	Male	Deep	0.639097744	3.038461538	2.493589744	0.493589744	III	Severe
FR22704263	Male	Deep	0.312101911	3.098765432	2.796296296	0.537037037	IV	Severe
FR22704264	Female	Shallow	0.384615385	2.673333333	2.44	0.6	III	Severe
FR22704267	Male	Deep	0.307692308	2.207792208	1.902597403	0.262820513	IV	Severe
FR22704273	Female	Deep	2.194805195	3.916666667	2.351851852	0.407407407	III	Severe
FR22704274	Male	Shallow	0.694444444	2.783950617	2.320987654	0.617283951	III	Mild/Moderate
FR22704275	Male	Shallow	0.263636364	2.891025641	2.705128205	0.570512821	III	Mild/Moderate
FR22704276	Female	Deep	0.265432099	2.901234568	2.635802469	0.679012346	III	Mild/Moderate
FR22704282	Female	Deep	0.320895522	2.974358974	2.698717949	0.724358974	III	Mild/Moderate
FR22704284	Male	Deep	0.694444444	3.393333333	2.726666667	0.686666667	IV	Severe
FR22704285	Female	Shallow	0.439189189	3.660493827	3.259259259	0.938271605	II	Mild/Moderate
FR22704287	Male	Shallow	0.26446281	3.083333333	2.861111111	0.6875	III	Mild/Moderate
FR22704291	Male	Shallow	0.713333333	2.826666667	2.113333333	0.37037037	IV	Severe
FR22704292	Female	Deep	0.032258065	2.817460317	2.801587302	0.401515152	III	Mild/Moderate

FR22704293	Male	Shallow	0.355828221	3.017857143	2.672619048	0.416666667	IV	Severe
FR22704294	Female	Shallow	0.222222222	2	1.780141844	0.305555556	III	Severe
FR22704295	Male	Shallow	1.051282051	3.365384615	2.314102564	0.230769231	III	Mild/Moderate
FR22704298	Male	Shallow	0.181818182	2.928143713	2.748502994	0.678571429	II	Mild/Moderate
FR22704299	Female	Shallow	0.472	2.633333333	2.24	0.333333333	II	Mild/Moderate
FR22704300	Female	Shallow	0.531531532	2.533333333	2.14	0.133333333	III	Mild/Moderate
FR22704301	Female	Deep	0.230769231	2.976190476	2.761904762	0.416666667	III	Mild/Moderate
FR22704302	Male	Shallow	0.282051282	2.190789474	1.921052632	0.288461538	IV	Mild/Moderate
FR22704322	Female	Shallow	0.328671329	2.743055556	2.416666667	0.277777778	III	Mild/Moderate
FR22704323	Female	Shallow	1.356164384	3.536912752	2.208053691	0.6	III	Mild/Moderate
FR22704325	Female	Shallow	0.163793103	2.666666667	2.522727273	0.424242424	III	Mild/Moderate
FR22704329	Male	Deep	0.224806202	2.153284672	1.948905109	0.347826087	IV	Mild/Moderate
FR22704332	Female	Deep	0.254658385	3.095238095	2.851190476	0.922619048	III	Mild/Moderate
FR22704337	Female	Deep	0.713235294	3.454545455	2.710144928	0.68115942	III	Mild/Moderate
FR22704346	Female	Shallow	0.313559322	2.788461538	2.551282051	0.820512821	IV	Severe
FR22704354	Female	Deep	0.328767123	3.080246914	2.783950617	0.654320988	III	Severe
FR22704356	Female	Shallow	1.32885906	3.733333333	2.413333333	0.06	III	Mild/Moderate
FR22704360	Female	Deep	0.261904762	2.163265306	1.945578231	0.2	III	Mild/Moderate
FR22704363	Male	Deep	0.422222222	2.166666667	1.913333333	0.266666667	III	Mild/Moderate
FR22704368	Female	Shallow	0.374045802	2.494047619	2.202380952	0.43452381	III	Severe
FR22704369	Female	Deep	0.762237762	2.881118881	2.118881119	0.6875	III	Severe
FR22704370	Male	Deep	0.481481481	3.117283951	2.635802469	0.5	III	Severe
FR22704374	Female	Deep	1.185185185	3.616766467	2.664670659	0.446428571	III	Severe
FR22704375	Female	Deep	1.375	3.130952381	1.755952381	0.023809524	III	Severe
FR22704377	Female	Shallow	1.351851852	3.962962963	2.611111111	0.697530864	III	Mild/Moderate
FR22704378	Male	Shallow	0.047619048	1.5	1.4625	0.05952381	III	Mild/Moderate
FR22704380	Male	Deep	0.234848485	2.01910828	1.840764331	0.12345679	III	Severe
FR22704381	Female	Shallow	0.328767123	3.080246914	2.783950617	0.654320988	III	Mild/Moderate
FR22704385	Female	Deep	1.15037594	3.166666667	2.104166667	0.180555556	III	Mild/Moderate
FR22704392	Female	Deep	0.224806202	2.153284672	1.948905109	0.347826087	IV	Severe
FR22704393	Female	Shallow	1.344262295	2.373333333	1.891025641	0.108974359	III	Mild/Moderate
FR22704394	Female	Shallow	1.545454545	2.583333333	1.470238095	0.071428571	III	Mild/Moderate
FR22704491	Female	Shallow	0.291338583	2.855072464	2.586956522	0.430555556	III	Mild/Moderate
FR22704492	Female	Deep	0.542056075	3.042735043	2.788617886	0.341269841	III	Mild/Moderate
FR22704495	Male	Shallow	1.065217391	2.208333333	1.868055556	0.263888889	IV	Severe
FR22704496	Male	Deep	0.552631579	2.693333333	2.273333333	0.2	IV	Severe
FR22704498	Male	Deep	0.276315789	1.646258503	1.510204082	0.226666667	IV	Severe
FR22704500	Male	Shallow	0.575757576	3.282051282	2.794871795	0.352564103	II	Mild/Moderate

FR22704501	Male	Shallow	1.469135802	3.808641975	2.339506173	0.172839506	III	Mild/Moderate
FR22704502	Male	Shallow	0.060810811	2.803571429	2.75	0.541666667	III	Severe
FR22704503	Male	Deep	1.583333333	4.224637681	2.985507246	0.528985507	III	Severe
FR22704504	Female	Deep	0.496296296	2.8125	2.347222222	0.284722222	III	Severe
FR22704505	Female	Shallow	0.40625	3.00617284	2.604938272	0.450617284	III	Severe
FR22704506	Male	Shallow	0.276315789	1.646258503	1.510204082	0.226666667	IV	Severe
FR22704507	Male	Shallow	0.201492537	2.779761905	2.619047619	0.345238095	III	Mild/Moderate
FR22704508	Male	Shallow	0.197530864	2.166666667	1.969135802	0.12345679	III	Mild/Moderate
FR22704509	Male	Deep	0.117283951	2.888888889	2.771604938	0.456790123	IV	Severe
FR22704510	Male	Shallow	0.442857143	2.74	2.326666667	0.273333333	IV	Severe
FR22704511	Female	Shallow	0.3125	2.839285714	2.541666667	0.43452381	III	Mild/Moderate
FR22704512	Female	Shallow	0.195945946	2.62962963	2.450617284	0.308641975	III	Severe
FR22704513	Female	Deep	0.095238095	2.299401198	2.203592814	0.410714286	III	Severe
FR22704514	Male	Shallow	0.575757576	3.282051282	2.794871795	0.352564103	II	No periodontitis
FR22704515	Female	Shallow	0.095238095	2.299401198	2.203592814	0.410714286	III	Mild/Moderate
FR22704571	Female	Shallow	0.185840708	3.397163121	3.24822695	0.701388889	III	Mild/Moderate
FR22704575	Male	Shallow	0.850393701	3.347222222	2.597222222	0.569444444	II	Mild/Moderate
FR22704576	Male	Shallow	0.197183099	2.576923077	2.397435897	0.41025641	III	Mild/Moderate
FR22704577	Male	Deep	0.26446281	3.083333333	2.861111111	0.6875	III	Mild/Moderate
FR22704578	Female	Shallow	0.064102564	2.551282051	2.487654321	0.185185185	III	Mild/Moderate
FR22704580	Male	Deep	0.40625	3.00617284	2.604938272	0.450617284	II	Mild/Moderate
FR22704581	Male	Shallow	0.064102564	2.019230769	1.955128205	0.429487179	II	Mild/Moderate
FR22704582	Male	Deep	0.197183099	2.576923077	2.397435897	0.41025641	III	Mild/Moderate
FR22704583	Male	Shallow	0.064102564	2.019230769	1.955128205	0.429487179	III	Mild/Moderate
FR22704584	Female	Deep	0.320512821	2.892857143	2.595238095	0.69047619	III	Mild/Moderate
FR22704585	Female	Shallow	0.201492537	2.779761905	2.619047619	0.345238095	III	Mild/Moderate
FR22704586	Male	Shallow	0.629213483	3.198019802	2.643564356	0.352941176	III	Mild/Moderate
FR22704587	Female	Deep	0.144827586	3.05952381	2.93452381	0.416666667	IV	Severe
FR22704588	Female	Deep	1.138888889	3.234567901	2.475308642	0.432098765	III	Severe
FR22704589	Female	Shallow	2.194690265	4.298611111	2.576388889	0.513888889	III	Severe
FR22704591	Male	Shallow	0.176	2.134615385	1.993589744	0.096153846	IV	Severe
FR22704592	Male	Deep	0.93129771	2.953333333	2.14	0.466666667	IV	Severe
FR22704593	Male	Deep	0.842767296	3.37037037	2.543209877	0.524691358	III	Severe
FR22704594	Male	Shallow	0.410852713	2.601190476	2.285714286	0.55952381	III	Severe
FR22704595	Male	Deep	0.410852713	2.601190476	2.285714286	0.55952381	III	Mild/Moderate
FR22704596	Male	Shallow	0.868686869	3.142857143	2.46031746	0.363636364	III	Mild/Moderate
FR22704597	Female	Deep	0.293103448	3.212121212	3.083333333	0.553030303	III	Severe
FR22704598	Female	Shallow	0.230769231	1.814102564	1.641025641	0.083333333	III	Severe

FR22704599	Female	Shallow	0.071942446	2.057692308	1.993589744	0.147435897	II	Mild/Moderate
FR22704600	Female	Shallow	0.494047619	2.827380952	2.333333333	0.386904762	III	Mild/Moderate
FR22704601	Female	Shallow	0.361111111	2.465277778	2.104166667	0.118055556	III	Mild/Moderate
FR22704602	Female	Shallow	0.174242424	2.791666667	2.654761905	0.416666667	III	Mild/Moderate
FR22704603	Female	Shallow	1.102189781	3.33974359	2.371794872	0.711538462	II	No periodontitis
FR22704604	Male	Shallow	0.345864662	3.196428571	2.922619048	0.589285714	III	Mild/Moderate
FR22704605	Male	Shallow	0.413793103	3.077380952	2.720238095	0.5	III	Mild/Moderate
FR22704606	Female	Deep	0.299270073	3.180555556	2.895833333	0.416666667	IV	Severe
FR22704607	Female	Shallow	0.230088496	3.24	3.066666667	0.62	IV	Severe
FR22704608	Female	Deep	0.364197531	3.358024691	3.011904762	0.279761905	III	Mild/Moderate
FR22704609	Female	Shallow	0.185185185	2.857142857	2.678571429	0.285714286	III	Mild/Moderate
FR22704611	Female	Deep	0.21641791	2.814814815	2.635802469	0.333333333	III	Mild/Moderate
FR22704613	Female	Shallow	0.185185185	3.30952381	3.130952381	0.55952381	III	Severe
FR22704614	Female	Deep	0.864864865	2.77852349	2.134228188	0.307692308	III	Severe
FR22704616	Female	Deep	NA	NA	NA	NA	III	Severe
FR22704617	Female	Shallow	0.666666667	2.802469136	2.160493827	0.055555556	III	No periodontitis
FR22704618	Female	Shallow	0.976744186	2.74	1.9	0.16	III	Mild/Moderate
FR22704619	Male	Deep	0.253424658	2.647435897	2.41025641	0.596153846	III	Severe
FR22704620	Male	Shallow	0.25	2.422619048	2.214285714	0.291666667	III	Severe
FR22704621	Male	Shallow	0.326241135	2.703703704	2.464285714	0.55952381	III	Severe
FR22704622	Male	Deep	0.25	2.422619048	2.214285714	0.291666667	III	Severe
FR22704623	Male	Shallow	1.225225225	3.922580645	3.04516129	0.506410256	III	Mild/Moderate
FR22704624	Male	Shallow	0.246666667	1.953333333	1.706666667	0.133333333	IV	Severe
FR22704625	Female	Deep	0.35483871	2.516339869	2.379084967	0.173076923	IV	Severe
FR22704626	Female	Shallow	0.65034965	3.179012346	2.604938272	0.62345679	NA	NA
FR22704628	Female	Deep	0.244094488	2.972222222	2.756944444	0.590277778	IV	Severe
FR22704630	Male	Shallow	0.648648649	3.193333333	2.553333333	0.570512821	III	Severe
FR22704631	Male	Deep	0.701030928	4.166666667	3.570175439	0.49122807	III	Severe
FR22704632	Male	Deep	1.458333333	3.902777778	2.444444444	0.604166667	III	Severe
FR22704633	Male	Shallow	0.648648649	3.193333333	2.553333333	0.570512821	III	Severe
FR22704634	Female	Shallow	0.35483871	2.516339869	2.379084967	0.173076923	IV	Severe
FR22704635	Male	Deep	0.387387387	2.25	1.891666667	0.175	III	Mild/Moderate
FR22704636	Male	Shallow	0.493150685	3.216049383	2.771604938	0.37654321	III	Mild/Moderate
FR22704637	Female	Shallow	0.496855346	3.505952381	3.035714286	0.392857143	IV	Severe
FR22704638	Female	Deep	0.496855346	3.505952381	3.035714286	0.392857143	III	Mild/Moderate
FR22704639	Male	Deep	0.081300813	3.583333333	3.519230769	0.769230769	III	Mild/Moderate
FR22704640	Male	Shallow	0.685483871	3.243055556	2.652777778	0.541666667	III	Mild/Moderate
FR22704641	Male	Shallow	0.731343284	3.196078431	2.715686275	0.666666667	IV	Severe

FR22704642	Male	Deep	0.383458647	2.538461538	2.211538462	0.615384615	IV	Severe
FR22704644	Female	Shallow	0.493243243	3.166666667	2.716049383	0.481481481	III	Mild/Moderate
FR22704645	Male	Shallow	0.796875	2.826666667	2.146666667	0.166666667	II	Mild/Moderate
FR22704646	Male	Deep	0.796875	2.826666667	2.146666667	0.166666667	II	Mild/Moderate
FR22704647	Female	Deep	1.52293578	3.303030303	2.045454545	0.265151515	III	Mild/Moderate
FR22704648	Female	Shallow	0.596899225	2.506666667	1.993333333	0.293333333	III	Mild/Moderate
FR22704650	Male	Deep	0.511450382	2.333333333	1.93452381	0.214285714	III	Severe
FR22704652	Male	Deep	0.731343284	3.196078431	2.715686275	0.666666667	IV	Severe
FR22704653	Male	Shallow	0.59375	2.6	2.093333333	0.366666667	III	Severe
FR22704654	Male	Deep	0.59375	2.6	2.093333333	0.366666667	III	Severe
FR22704655	Male	Shallow	0.239520958	2.005952381	1.767857143	0.25	II	Mild/Moderate
FR22704656	Female	Shallow	0.339285714	1.863095238	1.523809524	0.44047619	III	Mild/Moderate
FR22704657	Male	Shallow	1.52293578	3.303030303	2.045454545	0.265151515	III	Severe
FR22704658	Male	Shallow	1.252631579	3.208333333	2.285714286	0.206349206	III	Mild/Moderate
FR22704659	Male	Shallow	0.487654321	2.481481481	1.99382716	0.382716049	III	Mild/Moderate
FR22704660	Male	Shallow	0.077380952	2.166666667	2.089285714	0.267857143	III	Mild/Moderate
FR22704661	Male	Deep	0.325581395	2.963768116	2.65942029	0.536231884	IV	Severe
FR22704662	Male	Shallow	0.623076923	2.700680272	2.149659864	0.28	IV	Severe
FR22704663	Female	Deep	0.268518519	2.294871795	2.108974359	0.525641026	IV	Mild/Moderate
FR22704665	Male	Shallow	0.23364486	2.952380952	2.753968254	0.555555556	IV	Mild/Moderate
FR22704667	Male	Deep	0.625	3.31547619	2.75	0.678571429	III	Mild/Moderate
FR22704668	Male	Shallow	0.47	2.862318841	2.52173913	0.536231884	III	Mild/Moderate
FR22704669	Female	Deep	0.144736842	2.911392405	2.772151899	0.388888889	IV	Severe
FR22704670	Female	Shallow	0.484848485	3.059259259	2.585185185	0.297101449	IV	Severe
FR22704672	Male	Deep	0.276119403	2.555555556	2.364197531	0.265432099	III	Mild/Moderate
FR22704673	Female	Shallow	0.756756757	3.202380952	2.535714286	0.404761905	I	No periodontitis
FR22704674	Male	Shallow	0.346666667	2.14	1.793333333	0.173333333	IV	Severe
FR22704676	Female	Shallow	0.662576687	2.732142857	2.089285714	0.136904762	IV	Severe
FR22704677	Female	Deep	0.268907563	2.730769231	2.525641026	0.525641026	IV	Severe
FR22704678	Male	Shallow	0.442307692	2.958333333	2.575	0.258333333	III	Mild/Moderate
FR22704680	Male	Shallow	0.625	3.31547619	2.75	0.678571429	III	Mild/Moderate
FR22704681	Male	Deep	0.149350649	2.740740741	2.598765432	0.549382716	III	Mild/Moderate
FR22704875	Male	Deep	3.223684211	4.480392157	2.175925926	0.481481481	III	Mild/Moderate
FR22704878	Female	Shallow	0.351190476	2.94047619	2.589285714	0.571428571	III	Mild/Moderate
FR22704879	Female	Shallow	0.713414634	2.517857143	1.821428571	0.196428571	III	Mild/Moderate
FR22704880	Female	Shallow	1.052631579	2.346153846	1.833333333	0.083333333	III	Mild/Moderate
FR22704881	Male	Shallow	0.658119658	3.076388889	2.56	0.126666667	III	Mild/Moderate
FR22704882	Male	Deep	0.658119658	3.076388889	2.56	0.126666667	III	Mild/Moderate

FR22704883	Male	Shallow	1.535947712	3.55952381	2.160714286	0.047619048	III	Mild/Moderate
FR22704884	Male	Deep	0.660377358	3.251497006	2.622754491	0.404761905	III	Mild/Moderate
FR22704885	Male	Deep	0.555555556	3.180555556	2.9375	0.347222222	IV	Severe
FR22704886	Male	Shallow	0.987654321	3.06547619	2.113095238	0.327380952	IV	Severe
FR22704889	Female	Shallow	1.048387097	3.254385965	2.684210526	0.307017544	III	Mild/Moderate
FR22704891	Female	Shallow	0.391891892	2.697530864	2.518518519	0.481481481	III	Mild/Moderate
FR22704892	Female	Deep	0.391891892	2.697530864	2.518518519	0.481481481	III	Mild/Moderate
FR22704893	Male	Deep	0.414814815	3.133333333	2.76	0.633333333	III	Mild/Moderate
FR22704894	Male	Shallow	0.414814815	3.133333333	2.76	0.633333333	III	Mild/Moderate
FR22704895	Female	Shallow	0.320754717	2.705128205	2.4	0.604938272	III	Mild/Moderate
FR22704896	Female	Shallow	0.386904762	3.172619048	2.785714286	0.535714286	III	Mild/Moderate
FR22704897	Male	Shallow	1.11971831	4.256944444	3.152777778	0.618055556	III	Severe
FR22704898	Male	Deep	1.11971831	4.256944444	3.152777778	0.618055556	III	Severe
FR22704899	Female	Shallow	0.946107784	3.648809524	2.708333333	0.523809524	III	Severe
FR22704900	Male	Shallow	0.046875	3.197530864	3.160493827	0.814814815	III	Mild/Moderate
FR22704901	Female	Deep	0.291044776	3.286666667	3.025641026	0.826923077	III	Severe
FR22704902	Male	Shallow	0.703225806	3.012345679	2.339506173	0.413580247	IV	Mild/Moderate
FR22704904	Male	Deep	0.661764706	3.012820513	2.435897436	0.512820513	III	Mild/Moderate
FR22704905	Female	Deep	0.488888889	3.083333333	2.821428571	0.726190476	III	Mild/Moderate
FR22704906	Male	Shallow	0.025157233	2.720238095	2.696428571	0.851190476	II	Mild/Moderate
FR22704907	Male	Deep	0.268292683	2.74691358	2.543209877	0.481481481	IV	Severe
FR22704908	Male	Shallow	0.983606557	3.473282443	2.557251908	0.265151515	IV	Severe
FR22704909	Female	Deep	0.628571429	3.986111111	3.375	0.701388889	IV	Severe
FR22704910	Female	Shallow	0.628571429	3.986111111	3.375	0.701388889	IV	Severe
FR22704911	Female	Deep	0.349056604	3.16	2.913333333	0.686666667	IV	Severe
FR22704912	Female	Shallow	0.01875	2.827380952	2.80952381	0.642857143	IV	Severe
FR22704913	Male	Deep	0.046875	3.197530864	3.160493827	0.814814815	III	Mild/Moderate
FR22704915	Female	Shallow	0.875912409	3.033333333	2.233333333	0.126666667	II	Mild/Moderate
FR22704916	Female	Shallow	0.626582278	3.09375	2.475	0.259259259	III	Severe
FR22704917	Female	Deep	0.626582278	3.09375	2.475	0.259259259	III	Severe
FR22704918	Female	Shallow	0.571428571	3.576388889	3.076388889	0.576388889	IV	Severe
FR22704919	Female	Deep	0.37012987	2.80952381	2.470238095	0.601190476	IV	Severe
FR22704920	Female	Shallow	0.381355932	3.12	2.82	0.533333333	III	Mild/Moderate
FR22704921	Female	Shallow	0.208333333	2.692307692	2.564102564	0.378205128	IV	Severe
FR22704922	Female	Deep	0.381355932	3.12	2.82	0.533333333	IV	Severe
FR22704924	Male	Shallow	0.826446281	3.472222222	2.777777778	0.652777778	III	Severe
FR22704925	Male	Deep	0.826446281	3.472222222	2.777777778	0.652777778	III	Severe
FR22704926	Female	Shallow	0.20952381	3.347222222	3.194444444	0.756944444	III	Mild/Moderate

FR22704927	Female	Shallow	0.216666667	2.679012346	2.518518519	0.796296296	IV	Severe
FR22704928	Female	Deep	0.365517241	3.057692308	2.797619048	0.511904762	IV	Severe
FR22704929	Male	Deep	0.684210526	3.543209877	2.981481481	0.586419753	III	Severe
FR22704930	Male	Shallow	0.365517241	3.057692308	2.797619048	0.511904762	III	Severe
FR22704931	Male	Deep	0.438356164	3.153333333	2.726666667	0.36	III	Severe
FR22704932	Male	Shallow	0.684210526	3.543209877	2.981481481	0.586419753	III	Severe
FR22704933	Female	Shallow	0.41322314	2.811594203	2.54	0.373333333	III	Mild/Moderate
FR22704934	Male	Shallow	0.146153846	2.564102564	2.442307692	0.685897436	III	Mild/Moderate
FR22704935	Female	Deep	0.538461538	3.101190476	2.601190476	0.31547619	III	Mild/Moderate
FR22704936	Female	Shallow	0.813253012	3.071428571	2.267857143	0.166666667	III	Severe
FR22704937	Female	Deep	0.324561404	2.708333333	2.451388889	0.694444444	III	Severe
FR22704938	Male	Shallow	0.144736842	2.911392405	2.772151899	0.388888889	III	Severe
FR22704939	Male	Deep	0.379084967	2.932098765	2.574074074	0.635802469	III	Severe
FR22704940	Female	Deep	0.268907563	2.730769231	2.525641026	0.525641026	III	Mild/Moderate
FR22704942	Male	Deep	0.381578947	2.452380952	2.107142857	0.238095238	III	Mild/Moderate
FR22704945	Male	Deep	0.276119403	2.555555556	2.364197531	0.265432099	III	Mild/Moderate
FR22704946	Male	Shallow	0.484848485	3.059259259	2.585185185	0.297101449	III	Mild/Moderate
FR22704947	Female	Shallow	0.604651163	2.4375	1.895833333	0.277777778	III	Mild/Moderate
FR22704948	Male	Shallow	1.353535354	3.297101449	2.326086957	0.231884058	III	Mild/Moderate
FR22704950	Female	Shallow	0.623076923	2.700680272	2.149659864	0.28	III	Mild/Moderate
FR22704951	Male	Shallow	0.333333333	2.708333333	2.416666667	0.279761905	III	Mild/Moderate
FR22704952	Male	Deep	0.365384615	3	2.660714286	0.375	III	Mild/Moderate
FR22704954	Male	Shallow	0.381578947	2.452380952	2.107142857	0.238095238	III	Mild/Moderate
FR22704955	Female	Deep	0.745283019	2.686666667	2.16	0.186666667	III	Severe
FR22704956	Female	Deep	0.493150685	2.648148148	2.203703704	0.462962963	IV	Severe
FR22704957	Male	Deep	1.353535354	3.297101449	2.326086957	0.231884058	III	Mild/Moderate
FR22704958	Female	Shallow	0.077380952	2.166666667	2.089285714	0.267857143	II	Mild/Moderate
FR22704959	Male	Shallow	0.325581395	2.963768116	2.65942029	0.536231884	IV	Severe
FR22704960	Female	Deep	0.415492958	2.7	2.306666667	0.56	III	Mild/Moderate
FR22704961	Female	Shallow	0.756756757	3.202380952	2.535714286	0.404761905	III	Mild/Moderate
FR22704963	Female	Shallow	0.442176871	2.5	2.083333333	0.275641026	III	Severe
FR22704964	Female	Shallow	0.230769231	1.923076923	1.692307692	0.102564103	IV	Severe
FR22704965	Male	Shallow	0.195652174	3.185185185	3.018518519	0.543209877	III	Mild/Moderate
FR22704966	Male	Shallow	0.487654321	2.481481481	1.99382716	0.382716049	III	Mild/Moderate
FR22704967	Male	Deep	0.246666667	1.92	1.673333333	0.226666667	IV	Severe
FR22704968	Male	Deep	0.415492958	2.7	2.306666667	0.56	III	Mild/Moderate
FR22704969	Female	Shallow	0.381944444	2.766666667	2.4	0.493333333	IV	Mild/Moderate
FR22704970	Female	Deep	1.178571429	3.986577181	3.322147651	0.793333333	III	Mild/Moderate

FR22707852	Female	Shallow	0.754098361	3.731884058	3.065217391	0.485507246	IV	Severe
FR22707853	Female	Deep	0.754098361	3.731884058	3.065217391	0.485507246	IV	Severe
FR22707855	Female	Shallow	0.609756098	2.339285714	1.892857143	0.196428571	III	Mild/Moderate
FR22707856	Female	Deep	0.870503597	3.173333333	2.366666667	0.226666667	III	Severe
FR22707857	Female	Deep	1.262295082	2.944444444	1.875	0.145833333	III	Mild/Moderate
FR22707859	Female	Shallow	0.967105263	3.041666667	2.166666667	0.273809524	III	Mild/Moderate
FR22707860	Male	Shallow	0.122641509	1.833333333	1.75	0.25	IV	Mild/Moderate
FR22707861	Male	Shallow	0.195652174	3.185185185	3.018518519	0.543209877	II	Mild/Moderate
FR22707863	Female	Shallow	0.081300813	3.583333333	3.519230769	0.769230769	III	Mild/Moderate
FR22707864	Female	Deep	0.493150685	3.216049383	2.771604938	0.37654321	III	Mild/Moderate
FR22707865	Female	Shallow	0.493243243	3.166666667	2.716049383	0.481481481	II	No periodontitis
FR22707868	Male	Shallow	1.042735043	2.702380952	1.976190476	0.19047619	III	Mild/Moderate
FR22707869	Male	Deep	0.295454545	1.783950617	1.543209877	0.018518519	III	Mild/Moderate
FR22707870	Female	Shallow	0.21641791	2.814814815	2.635802469	0.333333333	III	Mild/Moderate
FR22707871	Male	Shallow	1.155405405	3.660493827	2.604938272	0.49382716	IV	Severe
FR22707872	Male	Deep	0.745901639	3.576923077	2.993589744	0.967948718	IV	Severe
FR22707873	Female	Shallow	0.253424658	2.647435897	2.41025641	0.596153846	III	Mild/Moderate
FR22707874	Female	Deep	0.266233766	3.051282051	2.788461538	0.455128205	III	Mild/Moderate
FR22707875	Male	Deep	1.225225225	3.922580645	3.04516129	0.506410256	IV	Mild/Moderate
FR22707876	Male	Deep	0.323353293	2.660714286	2.339285714	0.31547619	III	Severe
FR22707877	Female	Shallow	0.25	2.875	2.625	0.494047619	IV	Severe
FR22707878	Female	Deep	0.720588235	1.920634921	1.531746032	0.031746032	IV	Severe
FR22707879	Female	Shallow	0.244897959	2.08974359	1.935897436	0.134615385	IV	Severe
FR22707880	Male	Deep	0.055944056	1.880952381	1.833333333	0.095238095	III	Mild/Moderate
FR22707883	Male	Shallow	0.744444444	3.285714286	2.753968254	0.682539683	III	Mild/Moderate
FR22707884	Female	Shallow	1.392405063	2.513888889	1.75	0.055555556	III	Mild/Moderate
FR22707887	Male	Deep	0.083333333	1.738095238	1.660714286	0.053571429	IV	Severe
FR22707889	Female	Shallow	0.432	3.131944444	2.756944444	0.590277778	II	Mild/Moderate
FR22707890	Female	Shallow	0	2.267857143	2.267857143	0.178571429	III	Mild/Moderate
FR22707891	Male	Deep	0.113924051	2.886904762	2.779761905	0.386904762	III	Mild/Moderate
FR22707895	Female	Shallow	1.904109589	3.637681159	2.630434783	0.543478261	III	Mild/Moderate
FR22707898	Female	Shallow	1.904109589	3.637681159	2.630434783	0.543478261	III	Mild/Moderate
FR22707899	Female	Shallow	0.625	3.755952381	3.19047619	0.458333333	III	Mild/Moderate
FR22707900	Female	Deep	0.982758621	3.106666667	2.346666667	0.5	IV	Mild/Moderate
FR22707901	Male	Shallow	0.711538462	2.594405594	2.083916084	0.333333333	III	Mild/Moderate
FR22707902	Male	Deep	0.430107527	2.47826087	2.188405797	0.376811594	III	Mild/Moderate
FR22707904	Male	Deep	0.1	1.808641975	1.716049383	0.086419753	IV	Severe
FR22707906	Male	Deep	0.245508982	3.041666667	2.797619048	0.541666667	IV	Severe

FR22707909	Male	Shallow	0.226666667	1.970238095	1.767857143	0.035714286	III	Mild/Moderate
FR22707910	Male	Deep	1.319587629	3.055555556	2.166666667	0.236111111	IV	Severe
FR22707911	Male	Shallow	0.089285714	2.311377246	2.221556886	0.095238095	IV	Severe
FR22707913	Female	Shallow	0.244604317	2.160493827	1.950617284	0.191358025	III	Mild/Moderate
FR22707914	Female	Deep	1.181818182	3.26984127	2.238095238	0.087301587	IV	Severe
FR22707915	Female	Shallow	0.208333333	1.882716049	1.697530864	0.104938272	III	Mild/Moderate
FR22707916	Male	Deep	0.209150327	3.124183007	2.91503268	0.442307692	III	Severe
FR22707918	Female	Deep	0.227272727	2.230769231	2.038461538	0.121794872	III	Mild/Moderate
FR22707919	Female	Shallow	0.398550725	3.172619048	2.845238095	0.767857143	III	Mild/Moderate
FR22707920	Male	Shallow	0.532258065	3.210144928	2.731884058	0.637681159	IV	Severe
FR22707921	Male	Deep	0.532258065	3.210144928	2.731884058	0.637681159	IV	Severe
FR22707922	Female	Deep	0.047244094	1.992857143	1.957142857	0.111111111	III	Mild/Moderate
FR22707925	Female	Shallow	0.12962963	2.586666667	2.493333333	0.7	III	Mild/Moderate
FR22707926	Female	Shallow	0.810526316	3.513333333	3	0.446666667	III	Severe
FR22707928	Male	Deep	0.120689655	2.564102564	2.474358974	0.474358974	IV	Severe
FR22707929	Female	Deep	0.396825397	2.119047619	1.821428571	0.077380952	III	Severe
FR22707930	Female	Shallow	0.172661871	2.979166667	2.8125	0.722222222	III	Severe
FR22707931	Female	Deep	0.725925926	3.604938272	3	0.388888889	IV	Severe
FR22707932	Male	Shallow	0.172661871	2.979166667	2.8125	0.722222222	III	Severe
FR22707933	Male	Shallow	0.372413793	2.96	2.6	0.593333333	II	No periodontitis
FR22707934	Male	Deep	0.14516129	2.55	2.475	0.525	IV	Severe
FR22707935	Male	Shallow	0.941666667	3.24	2.486666667	0.433333333	III	Mild/Moderate
FR22707938	Female	Shallow	0.047244094	1.992857143	1.957142857	0.111111111	III	Mild/Moderate
FR22707939	Female	Shallow	0.725925926	3.604938272	3	0.388888889	II	Mild/Moderate
FR22707940	Female	Deep	0.398550725	3.172619048	2.845238095	0.767857143	III	Severe
FR22707942	Male	Shallow	0.132231405	2.636904762	2.541666667	0.613095238	IV	Severe
FR22707943	Female	Shallow	0.227642276	3.172619048	3.005952381	0.428571429	II	Mild/Moderate
FR22707944	Male	Deep	1.458333333	3.902777778	2.444444444	0.604166667	III	Mild/Moderate
FR22707945	Male	Shallow	0.232258065	2.685897436	2.455128205	0.438271605	III	Mild/Moderate
FR22707946	Female	Deep	0.244094488	2.972222222	2.756944444	0.590277778	III	Mild/Moderate
FR22707957	Female	Deep	0.571428571	2.523809524	2.071428571	0.261904762	III	Mild/Moderate
FR22707958	Female	Shallow	0.931818182	2.6875	2.402777778	0.583333333	III	Mild/Moderate
FR22707967	Male	Shallow	0.412587413	3.090277778	2.680555556	0.673611111	IV	Severe
FR22707972	Female	Deep	0.3	2.140350877	2.035087719	0.131578947	III	Mild/Moderate
FR22707975	Male	Shallow	0.174242424	2.791666667	2.654761905	0.416666667	IV	Severe
FR22707978	Male	Shallow	0.116666667	2.458333333	2.375	0.202380952	IV	Severe
FR22707982	Male	Shallow	2.492063492	3.51754386	2.125	0.212121212	III	Severe
FR22707985	Male	Shallow	1.070422535	3.692307692	2.717948718	0.596153846	III	Mild/Moderate

FR22707987	Female	Shallow	0.395833333	2.266666667	1.886666667	0.038461538	III	Severe
FR22707992	Male	Deep	1.169811321	3.224637681	2.775362319	0.456521739	IV	Severe
FR22707993	Male	Shallow	0.916666667	3.878787879	3.128787879	0.613636364	III	Mild/Moderate
FR22707997	Female	Deep	0.036697248	2.25	2.222222222	0.090277778	III	Severe
FR22707998	Female	Shallow	1.180645161	3.345238095	2.255952381	0.172619048	III	Mild/Moderate
FR22707999	Male	Shallow	0.326666667	2.404761905	2.113095238	0.095238095	II	No periodontitis
FR22708000	Female	Shallow	0.306666667	2.967948718	2.673076923	0.512820513	III	Mild/Moderate
FR22708002	Male	Shallow	0.386904762	2.425149701	2.035928144	0.113095238	III	Mild/Moderate
FR22708003	Male	Shallow	0.166666667	2.224358974	2.083333333	0.115384615	III	Mild/Moderate
FR22708004	Male	Shallow	0.23015873	2.961538462	2.775641026	0.608974359	III	Severe
FR22708005	Female	Shallow	0.765625	2.82	2.166666667	0.206666667	III	Mild/Moderate
FR22708007	Male	Deep	0.757575758	2.416666667	2	0.083333333	IV	Severe
FR22708008	Female	Shallow	3.75	6.722222222	2.972222222	0.444444444	III	Severe
FR22708014	Female	Shallow	2.394957983	4.14	2.24	0.2	IV	Severe
FR22708015	Male	Deep	0.806451613	3.089285714	2.494047619	0.339285714	III	Mild/Moderate
FR22708016	Female	Shallow	0.530612245	3.490909091	3.018181818	0.69047619	III	Severe
FR22708018	Female	Shallow	0.095238095	2.512820513	2.435897436	0.128205128	III	Severe
FR22708019	Female	Shallow	0.675213675	2.814102564	2.307692308	0.474358974	I	No periodontitis
FR22708020	Female	Deep	0.172839506	2.31547619	2.148809524	0.166666667	III	Mild/Moderate
FR22708023	Male	Shallow	1.180645161	3.214285714	2.125	0.095238095	II	Mild/Moderate
FR22708025	Female	Shallow	0.926229508	2.91025641	2.185897436	0.474358974	III	Mild/Moderate
FR22708026	Female	Shallow	0.052631579	2.297619048	2.261904762	0.083333333	III	Severe
FR22708027	Female	Shallow	1.210144928	2.875	1.880952381	0.029761905	II	Mild/Moderate
FR22708030	Male	Deep	1.444444444	3.654761905	3.071428571	0.753968254	III	Mild/Moderate
FR22708032	Male	Shallow	0.413043478	2.224637681	1.811594203	0.101449275	II	Mild/Moderate
FR22708033	Female	Shallow	0.274193548	2.213333333	1.986666667	0.153333333	I	No periodontitis
FR22708037	Female	Deep	0.380952381	2.375	2.041666667	0.357142857	III	Mild/Moderate
FR22708040	Female	Shallow	0.141176471	3.041666667	2.928571429	0.444444444	III	Mild/Moderate
FR22708141	Female	Deep	0.729166667	3.543209877	3.327160494	0.537037037	III	Mild/Moderate
FR22708153	Male	Shallow	0.348314607	2.166666667	1.982142857	0.148809524	II	No periodontitis
FR22708162	Female	Shallow	1.333333333	3.583333333	2.761904762	0.547619048	III	Mild/Moderate
FR22708170	Male	Shallow	0.43902439	3.086666667	2.846666667	0.533333333	IV	Severe
FR22708172	Female	Shallow	0.680555556	3.544871795	2.916666667	0.5	II	Mild/Moderate
FR22708175	Female	Shallow	0.494736842	2.597222222	2.270833333	0.354166667	III	Mild/Moderate
FR22708176	Female	Shallow	0.801526718	3.395833333	2.666666667	0.451388889	II	Mild/Moderate
FR22708177	Female	Deep	0.203883495	2.442176871	2.299319728	0.446666667	III	Severe
FR22708179	Male	Shallow	0.243589744	2.446969697	2.303030303	0.083333333	III	Mild/Moderate
FR22708180	Female	Deep	1.231884058	3.306666667	2.74	0.633333333	IV	Severe

FR22708183	Female	Deep	0.180451128	3.098765432	2.950617284	0.611111111	III	Mild/Moderate
FR22708185	Female	Shallow	0.24742268	2.416666667	2.262820513	0.384615385	III	Mild/Moderate
FR22708187	Female	Shallow	0.818181818	2.753333333	2.153333333	0.48	III	Mild/Moderate
FR22708188	Female	Deep	0.24742268	2.416666667	2.262820513	0.384615385	III	Mild/Moderate
FR22708190	Female	Deep	0.166666667	2.173611111	2.076388889	0.145833333	III	Severe
FR22708192	Female	Shallow	0.591836735	2.619047619	2.273809524	0.18452381	III	Mild/Moderate
FR22708194	Female	Shallow	0.071428571	2.423076923	2.365384615	0.269230769	III	Mild/Moderate
FR22708197	Female	Shallow	1.098901099	2.759259259	2.141975309	0.25308642	III	No periodontitis
FR22708198	Female	Shallow	NA	NA	NA	NA	III	Mild/Moderate
FR22708199	Female	Shallow	0.114035088	2	1.913333333	0.08	III	Severe
FR22708202	Female	Shallow	1.651685393	3.28030303	2.166666667	0.18115942	II	Mild/Moderate
FR22708205	Female	Shallow	0.731481481	2.453333333	1.926666667	0.026666667	III	Severe
FR22708206	Female	Deep	0.443298969	2.493333333	2.224358974	0.467948718	IV	Severe
FR22708208	Male	Shallow	0.621621622	2.401515152	2.227272727	0.356060606	III	Severe
FR22708214	Female	Shallow	0.898876404	3.625	3.069444444	0.472222222	III	Mild/Moderate
FR22708215	Male	Deep	0.43902439	3.086666667	2.846666667	0.533333333	IV	Severe
FR22708216	Female	Shallow	0.765625	2.82	2.166666667	0.206666667	II	Mild/Moderate
FR22708217	Male	Shallow	1.113636364	3.070512821	2.442307692	0.365384615	III	Mild/Moderate
FR22708218	Female	Shallow	0.742138365	2.987654321	2.259259259	0.265432099	III	Mild/Moderate
FR22708219	Female	Shallow	0.890909091	2.779761905	2.196428571	0.369047619	IV	Severe
FR22708220	Female	Shallow	0.547945205	2.833333333	2.320512821	0.5	II	Mild/Moderate
FR22708222	Female	Shallow	0.310810811	2.819875776	2.534161491	0.567901235	II	Mild/Moderate
FR22708223	Female	Shallow	1.870967742	4.447368421	3.446969697	0.515151515	III	Mild/Moderate
FR22708224	Female	Deep	0.801526718	3.395833333	2.666666667	0.451388889	II	Mild/Moderate
FR22708225	Female	Shallow	0.901234568	3.071428571	2.202380952	0.160714286	I	No periodontitis
FR22708226	Female	Deep	0.796052632	3.244047619	2.523809524	0.428571429	III	Mild/Moderate
FR22708228	Female	Shallow	0.896907216	3.333333333	2.570175439	0.49122807	II	Mild/Moderate
FR22708229	Male	Shallow	0.293103448	3.212121212	3.083333333	0.553030303	I	No periodontitis
FR22708230	Male	Shallow	NA	NA	NA	NA	II	Mild/Moderate
FR22708232	Male	Shallow	0.680555556	3.544871795	2.916666667	0.5	III	Mild/Moderate
FR22708234	Female	Shallow	0.206666667	2.549382716	2.358024691	0.083333333	III	Mild/Moderate
FR22708236	Female	Shallow	1.180645161	3.214285714	2.125	0.095238095	II	No periodontitis
FR22708256	Male	Shallow	0.629310345	2.326923077	1.858974359	0.467948718	III	Mild
FR22708261	Female	Shallow	0.154362416	2.740740741	2.598765432	0.481481481	II	Mild/Moderate
FR22708262	Female	Deep	1.169811321	3.224637681	2.775362319	0.456521739	III	Mild/Moderate
FR22708263	Female	Shallow	0.18	2.19379845	2.093023256	0.159090909	III	Mild/Moderate
FR22708264	Male	Shallow	0.227272727	2.173611111	2.069444444	0.166666667	III	Mild/Moderate
FR22708266	Male	Deep	0.15	1.685185185	1.574074074	0.030864198	III	Mild

FR22708271	Male	Deep	1.7	3.777777778	2.203703704	0.172839506	IV	Mild/Moderate
FR22708279	Female	Deep	0.9	2.814814815	1.981481481	0.265432099	IV	Severe
FR22708283	Female	Shallow	0.824	3.005952381	2.392857143	0.494047619	II	Mild/Moderate
FR22708287	Male	Shallow	0.881481481	2.75	2.041666667	0.5	III	Mild/Moderate
FR22708291	Female	Shallow	0.530612245	3.490909091	3.018181818	0.69047619	II	Mild/Moderate
FR22708295	Female	Shallow	0.887096774	3.26	2.526666667	0.046666667	II	Mild/Moderate
FR22708298	Male	Shallow	0.196969697	2.895833333	2.715277778	0.472222222	II	Mild/Moderate
FR22708306	Female	Shallow	1.241071429	3.320987654	2.462962963	0.271604938	II	Mild/Moderate
FR22708310	Male	Shallow	0.222222222	2.506944444	2.395833333	0.180555556	II	No periodontitis
FR22708313	Female	Deep	0.984496124	2.571428571	1.81547619	0.464285714	III	Mild/Moderate
FR22708320	Male	Shallow	1.416666667	2.6875	1.802083333	0	IV	Mild/Moderate
FR22708322	Female	Shallow	1.076271186	3.704697987	2.872483221	0.533333333	III	Mild/Moderate
FR23638168	Male	Shallow	1.00877193	3.33974359	2.602564103	0.519230769	III	Mild/Moderate
FR23638169	Female	Shallow	0.864864865	2.77852349	2.134228188	0.307692308	III	Mild/Moderate
FR23638170	Female	Shallow	0.296875	2.397435897	2.153846154	0.455128205	II	Mild/Moderate
FR23638171	Female	Deep	0.413793103	3.077380952	2.720238095	0.5	II	Mild/Moderate
FR23638172	Female	Shallow	0.214876033	2.625	2.470238095	0.327380952	III	Severe
FR23638173	Female	Shallow	0.601851852	2.788461538	2.371794872	0.403846154	III	Mild/Moderate
FR23638175	Male	Deep	1.067567568	4.032467532	3.006493506	0.666666667	III	Mild/Moderate
FR23638176	Female	Shallow	1.158730159	4.920289855	3.862318841	0.731884058	III	Mild/Moderate
FR23638178	Female	Shallow	0.5	3.16025641	2.756410256	0.621794872	II	No periodontitis
FR23638179	Female	Shallow	0.868686869	3.142857143	2.46031746	0.363636364	II	Mild/Moderate
FR23638180	Female	Shallow	NA	NA	NA	NA	IV	Mild/Moderate
FR23638181	Female	Deep	1.070422535	3.692307692	2.717948718	0.596153846	III	Severe
FR23638182	Male	Deep	0.281553398	2.807692308	2.621794872	0.192307692	IV	Severe
FR23638183	Male	Shallow	0.503937008	2.220238095	1.839285714	0.255952381	III	Mild/Moderate
FR23638184	Female	Shallow	2.488372093	3.806666667	2.38	0.14	II	Mild/Moderate
FR23638185	Male	Deep	0.285714286	1.951388889	1.701388889	0.083333333	III	Mild/Moderate
FR23638186	Male	Deep	0.987341772	3.37654321	2.413580247	0.327160494	IV	Severe
FR23638187	Male	Shallow	0.2	2.897435897	2.698717949	0.455128205	IV	Severe
FR23638188	Female	Shallow	0.378787879	2.104166667	1.756944444	0.041666667	III	Mild/Moderate
FR23638189	Female	Shallow	0.185185185	3.30952381	3.130952381	0.55952381	II	Mild/Moderate
FR23638190	Female	Deep	1.095238095	3.391025641	2.506410256	0.391025641	III	Severe
FR23638191	Female	Deep	0.459016393	2.591240876	2.197080292	0.594202899	III	Mild/Moderate
FR23638192	Female	Shallow	0.173913043	2.265151515	2.393333333	0.353333333	II	Mild/Moderate
FR23638193	Male	Shallow	0.446043165	2.592592593	2.267857143	0.31547619	III	Mild/Moderate
FR23638194	Male	Deep	0.909090909	2.8	2	0.213333333	III	Severe
FR23638195	Male	Shallow	1.967741935	2.507692308	2.038461538	0.022727273	III	Severe

FR23638196	Male	Shallow	0.930693069	2.773809524	2.214285714	0.291666667	III	Mild/Moderate
FR23638197	Female	Shallow	1.118181818	3.035714286	2.303571429	0.494047619	II	No periodontitis
FR23638198	Female	Shallow	0.451388889	2.907407407	2.50617284	0.37037037	III	Severe
FR23638199	Female	Shallow	0.417177914	2.511904762	2.107142857	0.267857143	III	Mild/Moderate
FR23638200	Male	Deep	0.930693069	2.773809524	2.214285714	0.291666667	III	Mild/Moderate
FR23638201	Female	Shallow	1.330708661	3.679012346	2.635802469	0.092592593	III	Mild/Moderate
FR23638202	Female	Deep	1.330708661	3.679012346	2.635802469	0.092592593	III	Mild/Moderate
FR23638203	Male	Deep	1.967741935	2.507692308	2.038461538	0.022727273	I	No periodontitis
FR23638204	Male	Shallow	0.224489796	2.479166667	2.326388889	0.416666667	I	No periodontitis
FR23638206	Female	Shallow	0.100840336	2.777777778	2.703703704	0.265432099	II	Mild/Moderate
FR23638207	Female	Shallow	0.657142857	2.691358025	2.12345679	0.160493827	III	Mild/Moderate
FR23638208	Female	Shallow	1.454545455	2.916666667	2.138888889	0.416666667	III	Mild/Moderate
FR23638209	Male	Shallow	0.365591398	2.342105263	2.043859649	0.12962963	IV	Severe
FR23638210	Female	Shallow	0.885057471	2.717948718	2.224358974	0.58974359	III	Mild/Moderate
FR23638211	Female	Deep	0.885057471	2.717948718	2.224358974	0.58974359	III	Mild/Moderate
FR23638212	Female	Deep	0.6	2.977272727	2.454545455	0.362318841	III	Mild/Moderate
FR23638213	Female	Shallow	0.141666667	2.62962963	2.524691358	0.432098765	III	Mild/Moderate
FR23638214	Female	Deep	1.454545455	2.916666667	2.138888889	0.416666667	III	Mild/Moderate
FR23638215	Female	Deep	1.118181818	3.035714286	2.303571429	0.494047619	III	Mild/Moderate
FR23638216	Female	Shallow	0.62962963	2.666666667	2.05952381	0.44047619	III	Mild/Moderate
FR23638217	Female	Deep	0.368421053	2.333333333	2.083333333	0.56547619	III	Mild/Moderate
FR23638218	Female	Shallow	0.62962963	2.666666667	2.05952381	0.44047619	III	Mild/Moderate
FR23638219	Female	Shallow	1.510204082	4.456790123	3.086419753	0.444444444	II	No periodontitis
FR23638220	Female	Shallow	0.78	2.611111111	2.301587302	0.452380952	III	Mild/Moderate
FR23638221	Female	Deep	1.510204082	4.456790123	3.086419753	0.444444444	III	Mild/Moderate
FR23638222	Male	Deep	0.643678161	2.333333333	1.953333333	0.113333333	IV	Severe
FR23638226	Female	Deep	0.351351351	2.557971014	2.275362319	0.18115942	IV	Severe
FR23638228	Female	Shallow	1.666666667	5.153333333	3.886666667	0.72	II	Mild/Moderate
FR23638229	Male	Shallow	0.852941176	2.725490196	1.859649123	0.035087719	II	Mild/Moderate
FR23638230	Female	Shallow	0.294117647	2.733333333	2.466666667	0.573333333	II	Mild/Moderate
FR23638231	Female	Shallow	0.25	2.622377622	2.566433566	0.506944444	III	Mild/Moderate
FR23638232	Male	Deep	0.748148148	3.18452381	2.583333333	0.279761905	III	Mild/Moderate
FR23638233	Female	Shallow	0.100840336	2.773809524	2.702380952	0.452380952	II	Mild/Moderate
FR23638234	Male	Shallow	1.067567568	4.032467532	3.006493506	0.666666667	II	Mild/Moderate
FR23638235	Female	Shallow	0.91954023	3.274509804	2.490196078	0.075757576	III	Mild/Moderate
FR23638237	Male	Deep	1.173913043	2.256944444	1.881944444	0.291666667	III	Mild/Moderate
FR23638238	Female	Deep	0.91954023	3.274509804	2.490196078	0.075757576	III	Mild/Moderate
FR23638239	Female	Deep	0.197674419	3.006666667	2.893333333	0.766666667	III	Severe

FR23638240	Male	Deep	1.383333333	3.307017544	2.566666667	0.558333333	III	Severe
FR23638241	Female	Shallow	0.193333333	2.533783784	2.318181818	0.628205128	III	Severe
FR23638243	Male	Shallow	0.230088496	3.24	3.066666667	0.62	III	Mild/Moderate
FR23638244	Female	Shallow	0.158730159	2.648148148	2.524691358	0.604938272	III	Severe
FR23638245	Male	Shallow	0.261538462	2.75	2.513888889	0.347222222	III	Mild/Moderate
FR23638246	Male	Shallow	0.188811189	2.571428571	2.410714286	0.255952381	IV	Mild/Moderate
FR23638247	Female	Shallow	0.9125	3.80952381	2.94047619	0.476190476	IV	Severe
FR23638248	Female	Deep	0.140350877	1.790123457	1.691358025	0.043209877	IV	Severe
FR23638249	Male	Shallow	0.66	3.906666667	3.246666667	0.586666667	IV	Severe
FR23638250	Female	Shallow	0.744444444	3.285714286	2.753968254	0.682539683	II	No periodontitis
FR23638251	Female	Shallow	0.348623853	3.233333333	2.98	0.533333333	III	Mild/Moderate
FR23638252	Male	Shallow	0.798387097	3.262820513	2.628205128	0.794871795	II	Mild/Moderate
FR23638256	Male	Shallow	0.691358025	3.369047619	2.702380952	0.31547619	III	Severe
FR23638257	Female	Shallow	1.158730159	4.920289855	3.862318841	0.731884058	III	Mild/Moderate
FR23638258	Female	Shallow	0.5	3.16025641	2.756410256	0.621794872	I	No periodontitis
FR23638259	Male	Shallow	0.194444444	2.213333333	2.073333333	0.14	III	Mild/Moderate
FR23638260	Male	Deep	0.364197531	3.358024691	3.011904762	0.279761905	III	Mild/Moderate
FR23638261	Male	Shallow	0.261538462	2.75	2.513888889	0.347222222	II	Mild/Moderate
FR23638262	Female	Deep	2.346153846	4.5	2.563492063	0.357142857	III	Mild/Moderate
FR23638263	Male	Shallow	0.813253012	3.244047619	2.44047619	0.613095238	III	Mild/Moderate
FR23638264	Male	Deep	0.173913043	3.029761905	2.863095238	0.845238095	III	Mild/Moderate
FR23638265	Male	Shallow	1.885714286	3.029761905	1.851190476	0.095238095	III	Mild/Moderate
FR23638266	Female	Shallow	0.937007874	3.404761905	2.696428571	0.380952381	III	Mild/Moderate
FR23638267	Female	Shallow	0.362204724	2.685185185	2.401234568	0.172839506	II	No periodontitis
FR23638268	Female	Shallow	0.370860927	3.275641026	2.916666667	0.564102564	II	No periodontitis
FR23638269	Male	Shallow	0.153846154	2.875	2.732142857	0.68452381	II	Mild/Moderate
FR23638270	Female	Deep	0.755102041	3.240740741	2.783950617	0.401234568	III	Severe
FR23638271	Male	Shallow	0.718309859	3.06	2.72	0.826666667	III	Mild/Moderate
FR23638272	Male	Shallow	0.261261261	3.011904762	2.839285714	0.791666667	III	Mild/Moderate
FR23638273	Male	Shallow	0.770992366	2.767857143	2.166666667	0.363095238	IV	Severe
FR23638274	Male	Deep	0.937007874	3.404761905	2.696428571	0.380952381	IV	Severe
FR23638275	Female	Shallow	0.9	2.756410256	2.006410256	0.41025641	III	Mild/Moderate
FR23638276	Female	Shallow	0.315789474	2.078431373	1.901960784	0.294117647	III	Mild/Moderate
FR23638278	Female	Shallow	0.064516129	2.785714286	2.738095238	0.369047619	II	Mild/Moderate
FR23638279	Male	Shallow	0.390728477	2.641975309	2.277777778	0.283950617	III	Mild/Moderate
FR23638280	Female	Shallow	0.171641791	2.94047619	2.803571429	0.630952381	IV	Mild/Moderate
FR23638281	Male	Deep	2.584745763	4.302816901	2.154929577	0.527777778	III	Severe
FR23638282	Male	Shallow	0.586956522	2.193333333	1.833333333	0.106666667	III	Severe

FR23638284	Female	Deep	0.733333333	4.073333333	3.34	0.593333333	III	Severe
FR23638285	Female	Shallow	0.179487179	1.987654321	1.814814815	0.086419753	III	Severe
FR23638286	Male	Deep	0.3	3.019230769	2.769230769	0.391025641	III	Severe
FR23638287	Female	Shallow	0.210144928	2.55952381	2.386904762	0.726190476	II	Mild/Moderate
FR23638289	Female	Deep	0.676056338	2.923611111	2.256944444	0.173611111	II	Mild/Moderate
FR23638290	Female	Deep	0.154362416	3.032051282	2.884615385	0.641025641	III	Severe
FR23638291	Female	Shallow	0.361344538	2.853333333	2.566666667	0.36	II	Mild/Moderate
FR23638292	Male	Deep	0.294117647	2.733333333	2.466666667	0.573333333	III	Severe
FR23638293	Male	Shallow	0.816666667	3.354166667	3.013888889	0.673611111	III	Severe
FR23638294	Male	Deep	0.813253012	3.244047619	2.44047619	0.613095238	III	Mild/Moderate
FR23638295	Male	Deep	1.583333333	3.1875	1.604166667	0.020833333	III	Mild/Moderate
FR23638298	Female	Shallow	0.284848485	2.80952381	2.529761905	0.535714286	III	Mild/Moderate
FR23638300	Male	Deep	0.6	3.046296296	2.518518519	0.648148148	IV	Severe
FR23638301	Male	Shallow	0.284848485	2.80952381	2.529761905	0.535714286	IV	Severe
FR23638303	Male	Shallow	0.272727273	2.285714286	2.071428571	0.06547619	III	Mild/Moderate
FR23638304	Male	Shallow	0.982758621	3.106666667	2.346666667	0.5	III	Mild/Moderate
FR23638305	Female	Shallow	0.218181818	3.153846154	3	0.570512821	II	Mild/Moderate
FR23638306	Male	Deep	0.149606299	2.032051282	1.91025641	0.051282051	IV	Severe
FR23638307	Male	Shallow	0.223214286	1.851190476	1.702380952	0.011904762	IV	Severe
FR23638308	Female	Shallow	0.9125	3.80952381	2.94047619	0.476190476	I	No periodontitis
FR23638309	Female	Shallow	0.208333333	2.077380952	1.928571429	0.077380952	II	Mild/Moderate
FR23638310	Male	Deep	0.083333333	1.974358974	1.891025641	0.08974359	IV	Severe
FR23638312	Male	Shallow	0.981651376	3.613636364	2.803030303	0.098484848	III	Mild/Moderate
FR23638313	Male	Deep	0	2.962962963	2.962962963	0.583333333	III	Mild/Moderate
FR23638314	Male	Shallow	0.108333333	2.726190476	2.648809524	0.363095238	III	Mild/Moderate
FR23638315	Female	Shallow	0.385714286	2.703703704	2.537037037	0.561728395	II	No periodontitis
FR23638317	Female	Shallow	0.434782609	2.924242424	2.772727273	0.545454545	II	Mild/Moderate
FR23638318	Male	Shallow	1.678899083	4.014492754	2.688405797	0.18115942	III	Mild/Moderate
FR23638319	Female	Shallow	0.67961165	2.575757576	2.048611111	0.104166667	III	Severe
FR23638320	Female	Deep	1.108108108	3.339285714	2.363095238	0.279761905	III	Severe
FR23638321	Male	Deep	0.742574257	3.230769231	2.75	0.467948718	IV	Severe
FR23638322	Male	Shallow	0.536912752	2.678571429	2.202380952	0.160714286	II	Mild/Moderate
FR23638323	Female	Shallow	0.25	2.622377622	2.566433566	0.506944444	III	Mild/Moderate
FR23638324	Female	Shallow	1.666666667	5.153333333	3.886666667	0.72	III	Mild/Moderate
FR23638328	Male	Shallow	0.6	2.977272727	2.454545455	0.362318841	III	Severe
FR23638329	Male	Deep	0.141666667	2.62962963	2.524691358	0.432098765	III	Severe
FR23638332	Female	Deep	0.62195122	2.326666667	1.986666667	0.34	III	Mild/Moderate
FR23638333	Female	Shallow	0.820689655	3.246666667	2.453333333	0.266666667	III	Mild/Moderate

FR23638334	Male	Shallow	0.6	2.830065359	2.653594771	0.653846154	III	Mild/Moderate
FR23638335	Male	Shallow	0.100840336	2.773809524	2.702380952	0.452380952	II	Mild/Moderate
FR23638338	Male	Deep	1.208333333	3.1875	1.979166667	0.305555556	III	Severe
FR23638339	Female	Deep	0.527777778	3.047619048	2.595238095	0.577380952	III	Severe
FR23638341	Female	Shallow	0.745283019	2.686666667	2.16	0.186666667	II	Mild/Moderate
FR23638342	Male	Deep	0.6	2.830065359	2.653594771	0.653846154	III	Mild/Moderate
FR23638343	Male	Shallow	1.076271186	3.704697987	2.872483221	0.533333333	III	Mild/Moderate
FR23638344	Female	Deep	0.509803922	2.211538462	1.711538462	0.076923077	III	Mild/Moderate
FR23638346	Female	Shallow	0.465277778	2.94047619	2.541666667	0.43452381	IV	Severe
FR23638347	Female	Deep	0.348623853	3.233333333	2.98	0.533333333	IV	Severe
FR23638348	Female	Deep	0.281553398	2.807692308	2.621794872	0.192307692	III	Mild/Moderate
FR23638349	Female	Shallow	1.322222222	3.897435897	3.134615385	0.378205128	IV	Severe
FR23638350	Female	Shallow	0.345864662	3.196428571	2.922619048	0.589285714	III	Mild/Moderate
FR23638352	Female	Shallow	1.102189781	3.33974359	2.371794872	0.711538462	III	Mild/Moderate
FR23638353	Male	Shallow	0.220472441	1.942307692	1.762820513	0.051282051	III	Mild/Moderate
FR23638354	Male	Deep	0.858490566	3.515873016	2.810606061	0.393939394	III	Mild/Moderate
FR23638355	Female	Shallow	0.585714286	2.910714286	2.422619048	0.547619048	III	Severe
FR23638356	Female	Deep	0.432	3.131944444	2.756944444	0.590277778	III	Severe
FR23638357	Female	Shallow	0.71875	2.033333333	1.573333333	0.02	IV	Severe
FR23638358	Female	Deep	0.032608696	1.807692308	1.788461538	0.064102564	IV	Severe
FR23638361	Male	Shallow	1.905660377	2.966666667	2.125	0.216666667	III	Mild/Moderate
FR23638364	Female	Shallow	0.193333333	2.533783784	2.318181818	0.628205128	III	Mild/Moderate
FR23638374	Female	Shallow	0.48	2.087301587	1.896825397	0.031746032	III	Mild/Moderate
FR23638376	Female	Shallow	0.333333333	2.296296296	2.185185185	0.277777778	II	Mild/Moderate
FR23638381	Male	Shallow	0.949152542	3.785714286	2.896825397	0.642857143	IV	Severe
FR23638388	Female	Shallow	0.415730337	2.714285714	2.494047619	0.267857143	III	Severe
FR23638390	Female	Shallow	0.138461538	2.99382716	2.882716049	0.765432099	III	Mild/Moderate
FR23638391	Male	Shallow	0.370860927	3.275641026	2.916666667	0.564102564	III	Mild/Moderate
FR23638392	Female	Shallow	0.78313253	2.759259259	2.358024691	0.382716049	III	Mild/Moderate
FR23638405	Male	Shallow	0.430379747	3.095238095	2.69047619	0.505952381	IV	Severe
FR23638415	Male	Deep	0.492063492	2.666666667	2.283950617	0.407407407	III	Mild/Moderate
FR23638416	Male	Shallow	0.138461538	2.99382716	2.882716049	0.765432099	III	Mild/Moderate
FR23638436	Female	Shallow	0.594594595	2.376811594	2.217391304	0.246376812	III	Mild/Moderate
FR23638439	Female	Deep	0.164285714	2.101190476	1.964285714	0.053571429	III	Severe
FR23638446	Female	Deep	0.368421053	3.557971014	3.253623188	0.855072464	III	Mild/Moderate
FR23638447	Male	Shallow	2.195121951	3.477272727	2.795454545	0.628787879	II	Mild/Moderate
FR23638448	Male	Deep	0.175438596	2.820987654	2.697530864	0.567901235	III	Mild/Moderate
FR23638840	Male	Deep	0.361344538	2.853333333	2.566666667	0.36	IV	Severe

FR23638856	Male	Shallow	0.336363636	3.104166667	2.847222222	0.513888889	III	Severe
FR23638858	Male	Deep	0.283950617	2.271604938	1.987654321	0.111111111	IV	Severe
FR23638864	Female	Deep	0.158273381	1.922619048	1.791666667	0.047619048	III	Severe
FR23638865	Female	Shallow	0.251851852	2.270833333	2.034722222	0.1875	III	Mild/Moderate
FR23638871	Female	Shallow	0.47008547	2.480769231	2.128205128	0.33974359	III	Severe
FR23638872	Male	Shallow	0.590551181	3.154761905	2.708333333	0.80952381	III	Mild/Moderate
FR23638881	Female	Shallow	0.116129032	2.5	2.392857143	0.607142857	II	Mild/Moderate
FR23638882	Male	Deep	0.843971631	2.878205128	2.115384615	0.66025641	II	Mild/Moderate
FR23638886	Female	Shallow	0.035211268	2.833333333	2.802469136	0.5	III	Mild/Moderate
FR23638887	Female	Deep	0.625899281	4.133333333	3.553333333	0.426666667	III	Mild/Moderate
FR23638889	Female	Shallow	0.188811189	2.571428571	2.410714286	0.255952381	I	No periodontitis
FR23638891	Male	Deep	0.426666667	3.032051282	2.621794872	0.288461538	III	Severe
FR23638897	Female	Shallow	2.377777778	3.602564103	2.366666667	0.408333333	III	Mild/Moderate
FR23638898	Male	Shallow	0.434782609	2.924242424	2.772727273	0.545454545	IV	Severe
FR23638902	Female	Shallow	0.653225806	3.277777778	2.715277778	0.555555556	III	Mild/Moderate
FR23638904	Female	Shallow	0.116129032	2.5	2.392857143	0.607142857	III	No periodontitis
FR23638906	Male	Deep	1.058139535	3.493055556	2.861111111	0.590277778	IV	Severe
FR23638907	Male	Shallow	0.426666667	3.032051282	2.621794872	0.288461538	III	Severe
FR23638910	Female	Deep	0.317460317	2.055555556	1.808641975	0.024691358	III	Mild/Moderate
FR23638915	Female	Deep	0.108333333	2.726190476	2.648809524	0.363095238	III	Mild/Moderate
FR23638916	Female	Deep	0.47008547	2.480769231	2.128205128	0.33974359	III	Severe
FR23638918	Male	Deep	0.134751773	2.470238095	2.357142857	0.535714286	IV	Severe
FR23638921	Female	Deep	2.377777778	3.602564103	2.366666667	0.408333333	IV	Severe
FR23638923	Female	Shallow	0.087719298	2.409722222	2.340277778	0.236111111	III	Mild/Moderate
FR23638924	Male	Shallow	0.095238095	2.31547619	2.232142857	0.291666667	III	Mild/Moderate
FR23638932	Male	Shallow	1.027777778	2.333333333	1.716666667	0.066666667	I	No periodontitis
FR23638996	Female	Shallow	0.013986014	2.988095238	2.976190476	0.613095238	III	Mild/Moderate
FR23639006	Female	Shallow	0.267241379	2.9375	2.722222222	0.506944444	III	Mild/Moderate
FR23639014	Male	Deep	0.603053435	3.375	2.904761905	0.613095238	IV	Severe
FR23639036	Female	Shallow	0.444444444	2.601190476	2.220238095	0.136904762	III	Mild/Moderate
FR23639038	Male	Shallow	0.451388889	2.907407407	2.50617284	0.37037037	II	Mild/Moderate
FR23639043	Male	Deep	0.739726027	2.289855072	1.898550725	0.384057971	III	Mild/Moderate
FR23639045	Female	Shallow	0.373626374	3	2.762237762	0.784722222	III	Mild/Moderate
FR23639056	Male	Shallow	0.337209302	1.974842767	1.79245283	0.098765432	III	Mild/Moderate
FR23639069	Female	Shallow	0.983606557	2.438271605	2.067901235	0.327160494	II	No periodontitis
FR23639116	Male	Shallow	0	2.962962963	2.962962963	0.583333333	III	Mild/Moderate
FR23639121	Female	Shallow	0.022727273	2.930555556	2.916666667	0.777777778	III	Mild/Moderate
FR23639124	Female	Shallow	0.412587413	3.090277778	2.680555556	0.673611111	II	Mild/Moderate

FR23639125	Female	Deep	0.683870968	2.714285714	2.083333333	0.511904762	III	Mild/Moderate
FR23667932	Male	Shallow	0.237179487	2.192307692	1.955128205	0.230769231	III	Mild/Moderate
FR23668007	Male	Shallow	0.357142857	2.208333333	1.851190476	0.089285714	II	Mild/Moderate
FR23668013	Female	Shallow	0.615384615	3.101190476	2.672619048	0.363095238	III	Mild/Moderate
FR23668127	Male	Shallow	0.815789474	3.833333333	3.067901235	0.364197531	IV	Severe
FR23668169	Male	Deep	0.873134328	3.555555556	2.833333333	0.636904762	IV	Severe
FR23668183	Female	Shallow	0.314285714	2.475308642	2.271604938	0.407407407	III	Mild/Moderate
FR23668184	Female	Deep	0.5625	2.631944444	2.444444444	0.5	II	Mild/Moderate
FR23668185	Male	Deep	0.815789474	3.833333333	3.067901235	0.364197531	IV	Severe
FR23668186	Female	Deep	0.88	2.404761905	1.880952381	0.063492063	II	Mild/Moderate
FR23668187	Female	Shallow	0.682926829	2.55952381	2.05952381	0.19047619	II	Mild/Moderate
FR23668188	Male	Shallow	0.453333333	2	1.811594203	0.130434783	III	Severe
FR23668189	Male	Deep	0.494117647	2.37037037	2.111111111	0.438271605	III	Severe
FR23668190	Female	Deep	1.088888889	2.698717949	2.070512821	0.147435897	III	Mild/Moderate
FR23668192	Female	Shallow	1.642335766	3.626666667	2.126666667	0.233333333	II	Mild/Moderate
FR23668193	Female	Shallow	0.816666667	2.808383234	2.22754491	0.267857143	III	Mild/Moderate
FR23668194	Female	Shallow	0.28030303	2.422619048	2.202380952	0.636904762	IV	Severe
FR23668195	Female	Deep	0.28030303	2.422619048	2.202380952	0.636904762	IV	Severe
FR23668196	Female	Shallow	0.905660377	3.158730159	2.396825397	0.341269841	II	Mild/Moderate
FR23668197	Female	Shallow	0.605633803	2.720238095	2.208333333	0.273809524	III	Mild/Moderate
FR23668198	Male	Shallow	0.363636364	2.469135802	2.222222222	0.222222222	IV	Severe
FR23668199	Male	Deep	1.744444444	2.922619048	1.988095238	0.130952381	IV	Severe
FR23668200	Male	Deep	0.363636364	2.469135802	2.222222222	0.222222222	III	Mild/Moderate
FR23668201	Male	Shallow	2.206896552	2.891666667	2.348484848	0.181818182	III	Mild/Moderate
FR23668203	Female	Deep	0.198717949	2.519230769	2.320512821	0.474358974	IV	Severe
FR23668204	Male	Shallow	0.411764706	2.327380952	1.994047619	0.125	II	Mild/Moderate
FR23668207	Male	Deep	0.254385965	2.407407407	2.220238095	0.101190476	IV	Severe
FR23668208	Male	Shallow	0.360294118	2.646666667	2.32	0.72	III	Mild/Moderate
FR23668211	Male	Deep	0.444444444	2.432098765	2.086419753	0.024691358	II	Mild/Moderate
FR23668212	Female	Shallow	1.356164384	3.536912752	2.208053691	0.6	IV	Severe
FR23668213	Male	Shallow	1.514285714	2.430555556	2.0625	0.097222222	III	Mild/Moderate
FR23668214	Female	Shallow	2.265306122	3.552083333	2.452380952	0.103174603	II	Mild/Moderate
FR23668215	Female	Shallow	0.571428571	2.425925926	2.12962963	0.12962963	III	Mild/Moderate
FR23668304	Male	Shallow	0.472727273	2.573333333	2.226666667	0.206666667	II	No periodontitis
FR23668307	Male	Shallow	0.774774775	2.557692308	2.006410256	0.391025641	II	No periodontitis
FR23668308	Male	Shallow	0.742574257	3.230769231	2.75	0.467948718	IV	Severe
FR23668309	Female	Deep	0.133333333	2.419753086	2.320987654	0.703703704	IV	Severe
FR23668310	Female	Shallow	0.354037267	3.857142857	3.517857143	0.43452381	III	Severe

FR23668311	Female	Shallow	0.211538462	2.703703704	2.5	0.098765432	I	No periodontitis
FR23668890	Male	Deep	0.648148148	2.99382716	2.345679012	0.37654321	IV	Mild/Moderate
FR23668891	Male	Shallow	0.616352201	3.36746988	2.777108434	0.321428571	IV	Mild/Moderate
FR23668892	Female	Shallow	0.875	3.904761905	3.515873016	0.825396825	III	Mild/Moderate
FR23668893	Female	Deep	0.492063492	3.452380952	3.083333333	0.571428571	III	Mild/Moderate
FR23668894	Male	Shallow	0.875	3.904761905	3.515873016	0.825396825	III	Mild/Moderate
FR23668896	Female	Deep	0.397163121	2.590062112	2.242236025	0.314814815	IV	Mild/Moderate
FR23668897	Female	Shallow	0.057692308	1.81547619	1.761904762	0.017857143	IV	Mild/Moderate
FR23668899	Female	Shallow	0.516949153	2.630952381	2.267857143	0.595238095	III	Mild/Moderate
FR23668900	Female	Shallow	3.137931034	4.063492063	1.896825397	0.182539683	III	Mild/Moderate
FR23668901	Female	Deep	0.980392157	3.517857143	2.625	0.386904762	III	Mild/Moderate
FR23668902	Male	Deep	0.070422535	3.038461538	3.055555556	0.425925926	III	Mild/Moderate
FR23668903	Male	Shallow	0.075471698	2.654320988	2.580246914	0.598765432	III	Mild/Moderate
FR23668904	Male	Deep	0.397163121	2.590062112	2.242236025	0.314814815	III	Mild/Moderate
FR23668907	Female	Shallow	0.345864662	2.452380952	2.178571429	0.345238095	IV	Severe
FR23668908	Female	Deep	1.147540984	4.455128205	3.557692308	0.897435897	IV	Severe
FR23668909	Female	Shallow	0.511627907	2.925925926	2.518518519	0.469135802	III	Severe
FR23668911	Female	Deep	0.54375	3.041666667	2.523809524	0.708333333	III	Mild/Moderate
FR23668913	Male	Shallow	1.147540984	4.455128205	3.557692308	0.897435897	II	Mild/Moderate
FR23668916	Female	Shallow	0.192307692	2.154320988	2.030864198	0.481481481	III	Mild/Moderate
FR23668917	Female	Shallow	0.862318841	2.845238095	2.136904762	0.363095238	II	Mild/Moderate
FR23668918	Female	Deep	0.635658915	3.045751634	2.509803922	0.256410256	III	Severe
FR23668919	Female	Shallow	0.54375	3.041666667	2.523809524	0.708333333	III	Mild/Moderate
FR23668920	Male	Deep	0.273381295	2.00617284	1.771604938	0.074074074	III	Mild/Moderate
FR23668922	Male	Shallow	0.440366972	2.523809524	2.238095238	0.232142857	III	Mild/Moderate
FR23668924	Male	Shallow	0.718309859	2.608333333	2.183333333	0.275	IV	Severe
FR23668925	Male	Deep	0.718309859	2.608333333	2.183333333	0.275	IV	Severe
FR23668926	Female	Shallow	0.29787234	2.026666667	1.746666667	0.013333333	III	Severe
FR23668927	Female	Deep	0.725925926	2.62345679	2.018518519	0.12962963	III	Mild/Moderate
FR23668929	Male	Deep	0.208333333	2.049382716	1.864197531	0.092592593	III	Severe
FR23668930	Male	Shallow	0.975	3.049382716	2.327160494	0.808641975	III	Mild/Moderate
FR23668931	Male	Shallow	0.291666667	2.788461538	2.564102564	0.480769231	III	Mild/Moderate
FR23668932	Female	Shallow	0.291666667	2.788461538	2.564102564	0.480769231	II	Mild/Moderate
FR23668934	Male	Deep	0.571428571	2.715277778	2.409722222	0.527777778	IV	Severe
FR23668935	Female	Shallow	0.2	1.916666667	1.779761905	0.011904762	III	Mild/Moderate
FR23668936	Male	Deep	0.180451128	1.863354037	1.720496894	0.074074074	III	Severe
FR23668937	Male	Shallow	0.725490196	2.368055556	2.111111111	0.4375	III	Severe
FR23668939	Female	Deep	0.638888889	3.291666667	2.908333333	0.508333333	III	Mild/Moderate

FR23668940	Male	Deep	0.513333333	2.806666667	2.293333333	0.673333333	III	Mild/Moderate
FR23668942	Male	Shallow	0.571428571	2.715277778	2.409722222	0.527777778	IV	Severe
FR23668944	Male	Shallow	0.716666667	2.597222222	2	0.243055556	II	Mild/Moderate
FR23668946	Male	Shallow	1.560747664	3.137681159	1.951388889	0.263888889	III	Mild/Moderate
FR23668947	Female	Deep	0	2.458333333	2.633333333	0.95	IV	Severe
FR23668948	Male	Deep	0.192771084	3.213333333	3.106666667	0.66	IV	Severe
FR23668949	Female	Shallow	0.980582524	3.279761905	2.678571429	0.648809524	III	Mild/Moderate
FR23668952	Male	Shallow	0.413533835	2.217948718	1.865384615	0.237179487	III	Mild/Moderate
FR23668953	Female	Deep	0.618055556	3.067901235	2.518518519	0.481481481	III	Severe
FR23668955	Male	Shallow	1.291666667	4.5	3.588235294	0.68627451	IV	Severe
FR23668956	Female	Shallow	0.561983471	3.185185185	2.765432099	0.858024691	IV	Severe
FR23668957	Male	Shallow	0.571428571	2.43452381	2.029761905	0.214285714	IV	Severe
FR23668958	Male	Shallow	0.354166667	2.425925926	2.111111111	0.148148148	III	Mild/Moderate
FR23668959	Male	Shallow	1.397163121	3.487179487	2.224358974	0.294871795	III	Mild/Moderate
FR23668961	Female	Deep	0.721153846	3.833333333	3.238095238	0.507936508	III	Mild/Moderate
FR23668962	Female	Deep	0.181034483	1.738095238	1.613095238	0.113095238	III	Mild/Moderate
FR23668963	Male	Shallow	0.188976378	3.229166667	3.0625	0.479166667	IV	Severe
FR23668965	Female	Shallow	1.731034483	4.583333333	3.089285714	0.720238095	II	Mild/Moderate
FR23668967	Male	Deep	1.203389831	3.007936508	2.444444444	0.698412698	III	Mild/Moderate
FR23668968	Male	Shallow	0.198412698	2.926174497	2.758389262	0.446666667	IV	Severe
FR23668969	Male	Deep	0.120689655	2.285714286	2.202380952	0.297619048	IV	Severe
FR23668970	Female	Deep	1.240740741	3.111111111	2.579365079	0.126984127	IV	Severe
FR23668971	Male	Deep	0.708333333	2.425925926	1.796296296	0.067901235	IV	Severe
FR23668972	Male	Deep	0.675213675	2.814102564	2.307692308	0.474358974	III	Mild/Moderate
FR23668973	Male	Shallow	0.031847134	2.56547619	2.535714286	0.488095238	III	Mild/Moderate
FR23668974	Female	Deep	2.06779661	3.868055556	2.173611111	0.416666667	III	Severe
FR23668975	Male	Shallow	0.697674419	2.209876543	1.654320988	0.061728395	III	Mild/Moderate
FR23668976	Male	Deep	0.794117647	2.423076923	2.076923077	0.794871795	III	Severe
FR23668977	Male	Shallow	0.561983471	3.185185185	2.765432099	0.858024691	III	Severe
FR23668978	Female	Shallow	0	2.6	2.865248227	0.333333333	IV	Severe
FR23668979	Female	Shallow	0.721153846	3.833333333	3.238095238	0.507936508	III	Severe
FR23668980	Female	Shallow	0.651162791	2.833333333	2.333333333	0.148809524	II	Mild/Moderate
FR23668981	Female	Shallow	1.06779661	3.305555556	2.430555556	0.756944444	II	Mild/Moderate
FR23668982	Female	Shallow	0.762589928	3.230769231	2.551282051	0.288461538	III	Mild/Moderate
FR23668985	Male	Shallow	0.428571429	2.820512821	2.493589744	0.423076923	III	Mild/Moderate
FR23668986	Female	Shallow	0.141304348	2.895833333	2.805555556	0.534722222	III	Mild/Moderate
FR23668987	Female	Shallow	0.294642857	2.416666667	2.1875	0.138888889	III	Mild/Moderate
FR23668988	Female	Shallow	0.941558442	3.279761905	2.416666667	0.482142857	IV	Severe

FR23668990	Female	Shallow	0.319444444	2.851190476	2.577380952	0.547619048	III	Severe
FR23668991	Male	Shallow	0.087719298	2.409722222	2.340277778	0.236111111	IV	Severe
FR23668992	Female	Shallow	0.796296296	2.962962963	2.166666667	0.154320988	III	Mild/Moderate
FR23668995	Female	Deep	0.141304348	2.895833333	2.805555556	0.534722222	III	Mild/Moderate
FR23668996	Female	Deep	0.592	3.38	2.886666667	0.566666667	IV	Severe
FR23668997	Female	Deep	0.425373134	3.717948718	3.352564103	0.512820513	III	Severe
FR23669000	Female	Deep	2.492063492	3.51754386	2.125	0.212121212	III	Mild/Moderate
FR23669001	Female	Shallow	0.166666667	1.740740741	1.635802469	0.043209877	II	Mild/Moderate
FR23669002	Female	Shallow	0.088235294	2.061728395	2.00617284	0.092592593	III	Mild/Moderate
FR23669003	Female	Shallow	0.395683453	2.19047619	1.863095238	0.101190476	III	Mild/Moderate
FR23669004	Female	Shallow	1.054545455	3.670731707	2.963414634	0.613095238	I	No periodontitis
FR23669005	Male	Shallow	0.268518519	2.034722222	1.833333333	0.0625	I	No periodontitis
FR23669006	Male	Shallow	0.428571429	2.820512821	2.493589744	0.423076923	II	No periodontitis
FR23669007	Female	Shallow	0.986577181	3.346666667	2.366666667	0.54	I	No periodontitis
FR23669009	Female	Shallow	0.542056075	2.345679012	1.987654321	0.259259259	II	Mild/Moderate
FR23669010	Male	Shallow	1.260869565	2.333333333	1.988095238	0.074074074	II	No periodontitis
FR23669011	Female	Deep	0.734693878	3.35	2.75	0.641666667	III	Severe
FR23669012	Female	Shallow	0.6484375	2.478527607	2	0.035714286	III	Severe
FR23669014	Male	Deep	0.6484375	2.478527607	2	0.035714286	IV	Mild/Moderate
FR23669015	Male	Deep	2.655172414	4.072916667	2.518518519	0.259259259	IV	Severe
FR23669017	Female	Deep	0.218181818	3.153846154	3	0.570512821	III	Severe
FR23669018	Female	Shallow	1.416107383	3.524691358	2.222222222	0.425925926	III	Mild/Moderate
FR23669019	Female	Deep	1.874172185	3.351190476	1.666666667	0.011904762	III	Mild/Moderate
FR23669020	Female	Shallow	0.305555556	3.115384615	2.903846154	0.756410256	III	Mild/Moderate
FR23669021	Female	Shallow	1.5	2.601449275	2.471014493	0.615942029	II	Mild/Moderate
FR23669022	Female	Shallow	0.227642276	3.172619048	3.005952381	0.428571429	III	Severe
FR23669023	Female	Deep	0.192982456	1.910714286	1.779761905	0.023809524	III	Severe
FR23669024	Female	Deep	1.248120301	3.868055556	2.715277778	0.777777778	IV	Severe
FR23669026	Female	Deep	1.248120301	3.868055556	2.715277778	0.777777778	III	Mild/Moderate
FR23669027	Female	Shallow	0.465116279	2.191358025	1.944444444	0.240740741	III	Mild/Moderate
FR23669028	Female	Shallow	0.571428571	2.43452381	2.029761905	0.214285714	III	Mild/Moderate
FR23669030	Male	Shallow	0.303571429	1.910714286	1.607142857	0.029761905	III	Severe
FR23669031	Female	Deep	0.198412698	2.926174497	2.758389262	0.446666667	III	Mild/Moderate
FR23669032	Male	Deep	0.031847134	2.56547619	2.535714286	0.488095238	IV	Severe
FR23669033	Female	Shallow	0.168918919	2.288461538	2.128205128	0.070512821	II	No periodontitis
FR23669034	Female	Shallow	0.383838384	3.230769231	2.987179487	0.58974359	III	Mild/Moderate
FR23669035	Male	Shallow	0.492063492	3.452380952	3.083333333	0.571428571	I	No periodontitis
FR23669036	Male	Shallow	1.459677419	3.847826087	2.536231884	0.398550725	III	Mild/Moderate

FR23669037	Male	Shallow	0.652173913	3.254385965	2.859649123	0.728070175	III	Mild/Moderate
FR23669038	Female	Shallow	1.240740741	3.111111111	2.579365079	0.126984127	III	Mild/Moderate
FR23669039	Female	Shallow	0.256944444	2.530864198	2.302469136	0.691358025	III	Mild/Moderate
FR23669040	Male	Deep	0.619469027	3.229166667	2.743055556	0.472222222	IV	Severe
FR23669042	Male	Shallow	0.266055046	2	1.814102564	0.076923077	II	Mild/Moderate
FR23669043	Female	Shallow	0.351351351	2.708333333	2.549019608	0.746031746	II	Mild/Moderate
FR23669044	Female	Shallow	0.58041958	3.607142857	3.113095238	0.928571429	II	Mild/Moderate
FR23669046	Male	Shallow	0.619469027	3.229166667	2.743055556	0.472222222	II	Mild/Moderate
FR23669047	Male	Deep	0.527777778	3.147435897	2.66025641	0.628205128	II	Mild/Moderate
FR23669048	Male	Shallow	0.980582524	3.279761905	2.678571429	0.648809524	III	Mild/Moderate
FR23669049	Male	Shallow	0.471910112	3.524691358	3.265432099	0.511904762	II	Mild/Moderate
FR23669050	Female	Shallow	0.471910112	3.524691358	3.265432099	0.511904762	II	Mild/Moderate
FR23669051	Female	Shallow	0.798387097	2.732142857	2.142857143	0.244047619	IV	Severe
FR23669052	Female	Deep	1.299145299	2.801282051	1.826923077	0.08974359	IV	Severe
FR23669055	Female	Shallow	0.552380952	2.452380952	2.107142857	0.464285714	II	Mild/Moderate
FR23669056	Female	Shallow	0.626086957	2.642857143	2.214285714	0.535714286	III	Mild/Moderate
FR23669057	Female	Deep	0.626086957	2.642857143	2.214285714	0.535714286	III	Mild/Moderate
FR23669058	Female	Shallow	0.571428571	2.188118812	2.108910891	0.303921569	II	No periodontitis
FR23669059	Male	Deep	2.06779661	3.868055556	2.173611111	0.416666667	III	Mild/Moderate
FR23669061	Male	Shallow	0.604938272	2.462962963	1.858024691	0.043209877	III	Mild/Moderate
FR23669062	Female	Shallow	0.407894737	2.672619048	2.303571429	0.68452381	III	Mild/Moderate
FR23669063	Female	Deep	1	2.702898551	2.68115942	1	III	Mild/Moderate
FR23669064	Female	Deep	0.355555556	2.441666667	2.175	0.033333333	III	Mild/Moderate
FR23669065	Female	Shallow	1.054545455	3.670731707	2.963414634	0.613095238	II	No periodontitis
FR23669066	Female	Shallow	0.191666667	2.285714286	2.142857143	0.043209877	II	Mild/Moderate
FR23669067	Female	Shallow	0.266055046	2.02173913	1.811594203	0.050724638	III	Mild/Moderate
FR23669069	Male	Shallow	0.971698113	3.06547619	2.452380952	0.392857143	II	Mild/Moderate
FR23669070	Female	Shallow	0.9375	2.962962963	2.222222222	0.314814815	II	Mild/Moderate
FR23669071	Female	Shallow	0.235955056	2.25	2.104166667	0.659722222	III	Mild/Moderate
FR23669075	Male	Shallow	1.111111111	3.22	2.353333333	0.386666667	III	Mild/Moderate
FR23669076	Female	Shallow	0.488372093	2.487654321	2.358024691	0.765432099	III	Mild/Moderate
FR23669078	Male	Shallow	0.260869565	2.419753086	2.197530864	0.413580247	III	Mild/Moderate
FR23669087	Female	Shallow	0.828358209	2.907407407	2.222222222	0.148148148	II	Mild/Moderate
FR23669089	Female	Shallow	0.796052632	3.244047619	2.523809524	0.428571429	I	No periodontitis
FR23669094	Male	Shallow	0.621621622	2.401515152	2.227272727	0.356060606	III	Severe
FR23669095	Female	Deep	0.043478261	2.006666667	1.973333333	0.06	III	Mild/Moderate
FR23669103	Male	Shallow	1.11971831	3.564102564	2.544871795	0.871794872	III	Mild/Moderate
FR23669111	Female	Shallow	0.425373134	3.717948718	3.352564103	0.512820513	I	No periodontitis

FR23669113	Male	Shallow	1.651685393	3.28030303	2.166666667	0.18115942	III	Mild/Moderate
FR23669120	Male	Shallow	0.294871795	3.216049383	2.932098765	0.450617284	I	No periodontitis
FR23669121	Female	Shallow	0.196969697	2.895833333	2.715277778	0.472222222	III	Mild/Moderate
FR23669129	Female	Shallow	0.196428571	2.041666667	1.845238095	0.071428571	III	Mild/Moderate
FR23669130	Female	Deep	NA	NA	NA	NA	III	Severe
FR23669136	Female	Shallow	0.824	3.005952381	2.392857143	0.494047619	III	Mild/Moderate
FR23669137	Female	Deep	0.333333333	2.547445255	2.211678832	0.152173913	III	Mild/Moderate
FR23669138	Male	Deep	0.686046512	2.804347826	2.376811594	0.514492754	III	Severe
FR23669139	Female	Shallow	1.586666667	3.296296296	1.827160494	0.086419753	III	Severe
FR23669142	Female	Shallow	0.25	2.48	2.28	0.106666667	III	Severe
FR23669144	Male	Deep	0.536231884	2.93452381	2.494047619	0.208333333	III	Severe
FR23669145	Female	Shallow	1.542857143	3.645833333	2.520833333	0.090277778	III	Severe
FR23669146	Male	Shallow	0.380952381	2.571428571	2.19047619	0.05952381	III	Severe
FR23669147	Male	Deep	0	2.193333333	2.193333333	0.066666667	III	Mild/Moderate
FR23669153	Female	Deep	1.542857143	3.645833333	2.520833333	0.090277778	III	Severe
FR23669154	Female	Shallow	0.729166667	3.543209877	3.327160494	0.537037037	III	Mild/Moderate
FR23669156	Female	Shallow	0.214285714	3.595238095	3.380952381	0.541666667	III	Mild/Moderate
FR23669157	Female	Deep	3.75	6.722222222	2.972222222	0.444444444	III	Severe
FR23669163	Female	Shallow	0.744444444	2.987654321	2.574074074	0.351851852	III	Mild/Moderate
FR23669164	Female	Deep	0.105263158	2.090277778	2.006944444	0.090277778	III	Severe
FR23669166	Female	Shallow	0.041666667	1.779761905	1.738095238	0.029761905	IV	Severe
FR23669176	Male	Shallow	0.824675325	3.297619048	2.541666667	0.458333333	III	Mild/Moderate
FR23669177	Male	Deep	0.734513274	2.762820513	2.230769231	0.044871795	III	Severe
FR23669181	Female	Deep	0.090277778	2.011904762	1.93452381	0.119047619	III	Severe
FR23669190	Female	Deep	0.666666667	2.544444444	2.5	0.077777778	III	Severe
FR23669199	Male	Shallow	0.774774775	2.557692308	2.006410256	0.391025641	NA	NA
FR23669252	Male	Deep	0.051851852	1.75	1.708333333	0.041666667	III	Mild/Moderate
FR23669261	Female	Shallow	0.436090226	2.282051282	1.91025641	0.179487179	II	No periodontitis
FR23669262	Female	Deep	0.305555556	3.115384615	2.903846154	0.756410256	III	Severe
FR23669270	Male	Deep	0.591836735	2.619047619	2.273809524	0.18452381	III	Severe
FR23669368	Female	Deep	0.366197183	2.75	2.553030303	0.340909091	IV	Severe
FR23669372	Male	Shallow	0.203883495	2.442176871	2.299319728	0.446666667	III	Mild/Moderate
FR23669373	Male	Shallow	1.444444444	3.654761905	3.071428571	0.753968254	III	Mild/Moderate
FR23669375	Male	Shallow	0.614906832	3.089285714	2.5	0.529761905	I	No periodontitis
FR23669379	Male	Shallow	0.034482759	2.944444444	2.923611111	0.541666667	III	Mild/Moderate
FR23669420	Male	Shallow	1.241071429	3.320987654	2.462962963	0.271604938	III	Mild/Moderate
FR23669421	Male	Shallow	0.592	3.38	2.886666667	0.566666667	IV	Severe
FR23669424	Female	Deep	0.614906832	3.089285714	2.5	0.529761905	III	Mild/Moderate

FR23669427	Male	Deep	1.640350877	3.327160494	2.172839506	0.5	III	Severe
FR23669428	Male	Shallow	2.655172414	4.072916667	2.518518519	0.259259259	IV	Severe
FR23669430	Male	Shallow	0.2578125	3.102564103	2.891025641	0.724358974	III	Mild/Moderate
FR23669452	Female	Deep	1.214814815	3.154761905	2.178571429	0.416666667	III	Severe
FR23669466	Male	Shallow	0.6953125	2.452380952	1.922619048	0.101190476	III	Mild/Moderate
FR23669479	Male	Shallow	0.180451128	3.098765432	2.950617284	0.611111111	III	Severe
FR23669494	Male	Deep	0.104651163	2.123188406	2.057971014	0.079710145	IV	Severe
FR23669497	Male	Deep	0.890909091	2.779761905	2.196428571	0.369047619	IV	Severe
FR23669509	Female	Deep	0.898876404	3.625	3.069444444	0.472222222	III	Mild/Moderate
FR23669527	Female	Shallow	0.267241379	2.9375	2.722222222	0.506944444	II	Mild/Moderate
FR23669533	Male	Shallow	0.285714286	3.080246914	2.956790123	0.432098765	II	No periodontitis
FR23669538	Female	Deep	0.113924051	2.886904762	2.779761905	0.386904762	III	Mild/Moderate
FR23669551	Male	Shallow	2.394957983	4.14	2.24	0.2	IV	Mild/Moderate
FR23669553	Female	Shallow	0.061728395	1.660714286	1.601190476	0.101190476	II	Mild/Moderate
FR23669556	Female	Shallow	0.214285714	3.595238095	3.380952381	0.541666667	IV	Severe
FR23669558	Male	Shallow	0.230769231	2.56547619	2.458333333	0.505952381	IV	Mild/Moderate
FR23669560	Female	Shallow	0.382608696	2.197530864	1.925925926	0.12345679	III	Mild/Moderate
FR23669561	Female	Deep	1.00877193	3.33974359	2.602564103	0.519230769	III	Severe
FR23669562	Male	Shallow	1.031914894	2.626984127	1.857142857	0.420289855	II	Mild/Moderate
FR23669563	Male	Shallow	3.555555556	2.871212121	2.143939394	0.568181818	II	No periodontitis
FR23669565	Female	Deep	0.909722222	2.666666667	1.756944444	0.111111111	IV	Severe
FR23669566	Female	Shallow	3.555555556	2.871212121	2.143939394	0.568181818	II	Mild/Moderate
FR23669568	Female	Deep	0.355932203	2.185897436	2.051282051	0.025641026	III	Mild/Moderate
FR23669569	Female	Shallow	0.81884058	2.653846154	1.929487179	0.108974359	III	Mild/Moderate
FR23669571	Male	Deep	0.14084507	2.314814815	2.191358025	0.080246914	III	Mild/Moderate
FR23669572	Male	Shallow	0.842767296	3.37037037	2.543209877	0.524691358	III	Mild/Moderate
FR23669573	Female	Shallow	0.518518519	2.456790123	2.111111111	0.308641975	IV	Severe
FR23669574	Female	Deep	0.506493506	2.685897436	2.185897436	0.538461538	IV	Severe
FR23669575	Female	Shallow	0.843137255	3.075	2.358333333	0.325	III	Severe
FR23669576	Male	Deep	0.509259259	1.866666667	1.5	0.033333333	III	Mild/Moderate
FR23669577	Male	Shallow	1.214814815	3.154761905	2.178571429	0.416666667	III	Mild/Moderate
FR23669578	Male	Shallow	0.843137255	3.075	2.358333333	0.325	III	Severe
FR23669579	Male	Shallow	0.018181818	1.38961039	1.376623377	0.089285714	IV	Severe
FR23669580	Male	Deep	0.095890411	2.108974359	2.064102564	0.41025641	IV	Severe
FR23669582	Female	Shallow	0.484848485	2.160493827	1.864197531	0.086419753	III	Mild/Moderate
FR23669583	Female	Shallow	1.342342342	3.246376812	2.166666667	0.347826087	III	Mild/Moderate
FR23669584	Male	Shallow	1.342342342	3.246376812	2.166666667	0.347826087	IV	Severe
FR23669585	Male	Deep	1.042735043	2.702380952	1.976190476	0.19047619	IV	Severe

FR23669587	Female	Deep	0.52173913	2.297101449	1.775362319	0.057971014	III	Mild/Moderate
FR23669588	Female	Shallow	0.333333333	2.108695652	1.833333333	0.079710145	III	Mild/Moderate
FR23669589	Female	Deep	0.239130435	2.708333333	2.511904762	0.333333333	III	Mild/Moderate
FR23669590	Female	Shallow	1.375	3.256944444	2.263888889	0.368055556	III	Mild/Moderate
FR23669591	Female	Shallow	NA	NA	NA	NA	II	Mild/Moderate
FR23669592	Male	Deep	0.47826087	1.833333333	1.355072464	0.028985507	IV	Severe
FR23669593	Female	Deep	0.506329114	2.064102564	1.583333333	0.160493827	III	Mild/Moderate
FR23669594	Female	Shallow	1.048611111	2.794871795	1.826923077	0.018518519	III	Mild/Moderate
FR23669595	Female	Deep	0.188888889	2.135802469	2.030864198	0.092592593	IV	Severe
FR23669596	Female	Shallow	0.689393939	2.589285714	2.047619048	0.05952381	IV	Severe
FR23669597	Female	Deep	1.024193548	2.851851852	2.067901235	0.561728395	III	Severe
FR23669598	Male	Shallow	0.431818182	2.209876543	1.858024691	0.055555556	III	Mild/Moderate
FR23669601	Male	Shallow	0.78	1.755952381	1.523809524	0	III	Mild/Moderate
FR23669602	Male	Deep	0.384615385	1.888888889	1.518518519	0.080246914	IV	Severe
FR23669603	Male	Shallow	0.290780142	2.851851852	2.598765432	0.574074074	IV	Severe
FR23669604	Male	Shallow	0.387755102	2.234567901	1.882716049	0.049382716	III	Mild/Moderate
FR23669605	Male	Deep	0.245508982	3.041666667	2.797619048	0.541666667	III	Mild/Moderate
FR23669606	Male	Shallow	0.239130435	2.708333333	2.511904762	0.333333333	II	Mild/Moderate
FR23669607	Female	Shallow	NA	NA	NA	NA	III	Severe
FR23669609	Female	Shallow	0.25	2.875	2.625	0.494047619	III	Mild/Moderate
FR23669610	Female	Shallow	0.513333333	3.153333333	2.64	0.673333333	III	Mild/Moderate
FR23669611	Female	Deep	0.46969697	3.325301205	2.951807229	0.726190476	III	Mild/Moderate
FR23669612	Male	Deep	0.788461538	2.697530864	1.938271605	0.098765432	IV	Severe
FR23669613	Male	Shallow	0.62295082	2.773333333	2.266666667	0.3	IV	Severe
FR23669614	Male	Deep	0.457364341	3.28	2.886666667	0.553333333	IV	Severe
FR23669615	Male	Shallow	0.513333333	3.153333333	2.64	0.673333333	IV	Severe
FR23669619	Male	Deep	1.134615385	3.703703704	2.611111111	0.469135802	IV	Severe
FR23669620	Female	Deep	0.089285714	2.311377246	2.221556886	0.095238095	III	Mild/Moderate
FR23669621	Female	Shallow	1.134615385	3.703703704	2.611111111	0.469135802	III	Mild/Moderate
FR23669623	Male	Shallow	0.381818182	1.973154362	1.704697987	0.273333333	III	Mild/Moderate
FR23669625	Female	Shallow	0.120689655	2.564102564	2.474358974	0.474358974	IV	Severe
FR23669626	Female	Deep	0.810526316	3.513333333	3	0.446666667	IV	Severe
FR23669629	Female	Deep	0.308333333	2.725	2.416666667	0.458333333	III	Mild/Moderate
FR23669630	Female	Shallow	0.357142857	2.319444444	2.145833333	0.375	IV	Severe
FR23669631	Female	Deep	0.275	2.883333333	2.7	0.45	IV	Severe
FR23669632	Female	Deep	0.346534653	3.077380952	2.869047619	0.583333333	IV	Severe
FR23669633	Female	Shallow	0.638888889	2.019230769	1.724358974	0.057692308	IV	Severe
FR23669634	Male	Deep	1.291666667	4.5	3.588235294	0.68627451	III	Mild/Moderate

FR23669635	Male	Shallow	0	2.6	2.865248227	0.333333333	III	Mild/Moderate
FR23669636	Female	Shallow	0.120689655	2.285714286	2.202380952	0.297619048	III	Mild/Moderate
FR23669639	Male	Shallow	0.481012658	3.191358025	2.722222222	0.450617284	IV	Severe
FR23669640	Female	Shallow	1.203389831	3.007936508	2.444444444	0.698412698	II	Mild/Moderate
FR23669641	Male	Shallow	0.25	1.979166667	1.819444444	0.159722222	III	Mild/Moderate
FR23669643	Female	Deep	0.526315789	2.798611111	2.381944444	0.541666667	III	Mild/Moderate
FR23669644	Male	Shallow	0.213333333	2.821428571	2.630952381	0.279761905	IV	Severe
FR23669645	Male	Deep	0.941666667	3.24	2.486666667	0.433333333	IV	Severe
FR23669647	Male	Deep	0.34057971	2.178571429	1.898809524	0.071428571	IV	Severe
FR23669648	Male	Deep	0.721311475	3.546296296	3.138888889	0.861111111	III	Mild/Moderate
FR23669651	Female	Shallow	0.336	2.583333333	2.333333333	0.327380952	III	Mild/Moderate
FR23669652	Female	Deep	0.635658915	3.045751634	2.509803922	0.256410256	III	Mild/Moderate
FR23669654	Female	Shallow	0.134020619	2.625	2.534722222	0.638888889	III	Mild/Moderate
FR23669655	Female	Shallow	0.195652174	2.08	1.9	0.06	IV	Severe
FR23669657	Male	Shallow	0.303030303	2.535714286	2.238095238	0.142857143	IV	Severe
FR23669661	Male	Deep	0.820689655	3.089285714	2.380952381	0.375	III	Mild/Moderate
FR23669673	Female	Shallow	0.141176471	3.041666667	2.928571429	0.444444444	II	No periodontitis
FR23669677	Male	Deep	0.385542169	2.2875	2.0875	0.074074074	III	Severe
FR23669678	Male	Deep	0.916666667	3.878787879	3.128787879	0.613636364	III	Mild/Moderate
FR23669682	Female	Shallow	0.094117647	1.793333333	1.74	0.04	III	Mild/Moderate
FR23669694	Female	Shallow	0.542056075	2.345679012	1.987654321	0.259259259	I	No periodontitis
FR23669697	Male	Shallow	0.201388889	2.462962963	2.283950617	0.061728395	III	Mild/Moderate
FR23669707	Female	Shallow	0.178082192	2.243589744	2.16025641	0.108974359	I	No periodontitis
FR23669710	Male	Deep	0.286666667	3.125	2.869047619	0.458333333	III	Mild/Moderate
FR23669711	Male	Shallow	0.230769231	2.56547619	2.458333333	0.505952381	III	Mild/Moderate
FR23669717	Male	Shallow	0.068627451	1.987179487	1.942307692	0.070512821	IV	Severe
FR23669719	Female	Shallow	0.896907216	3.333333333	2.570175439	0.49122807	IV	Severe
FR23669720	Male	Deep	0.278911565	3.017857143	2.773809524	0.630952381	III	Mild/Moderate
FR23669721	Female	Shallow	0.306666667	2.967948718	2.673076923	0.512820513	III	Mild/Moderate
FR23669744	Male	Deep	0.231578947	2.641025641	2.5	0.602564103	III	Severe
FR23669747	Female	Deep	1.210144928	2.875	1.880952381	0.029761905	IV	Severe
FR23669750	Male	Shallow	1.640350877	3.327160494	2.172839506	0.5	III	Severe
FR23669752	Male	Shallow	0.59	2.9375	2.527777778	0.888888889	III	Mild/Moderate
FR23669781	Male	Shallow	0.984496124	2.571428571	1.81547619	0.464285714	II	Mild/Moderate
FR23669792	Female	Shallow	0.902255639	2.755952381	2.041666667	0.458333333	II	No periodontitis
FR23669794	Female	Shallow	0.173913043	2.178571429	2.05952381	0.196428571	III	Mild/Moderate
FR23669799	Female	Shallow	0.442477876	2.713333333	2.38	0.5	III	Mild/Moderate
FR23669800	Female	Deep	0.993464052	3.547619048	2.642857143	0.279761905	IV	Severe

FR23669803	Male	Deep	0.155737705	2.547619048	2.43452381	0.476190476	IV	Severe
FR23669805	Female	Shallow	0.873134328	3.555555556	2.833333333	0.636904762	I	No periodontitis
FR23669811	Male	Shallow	0.278911565	3.017857143	2.773809524	0.630952381	IV	Severe
FR23669814	Female	Deep	0.723684211	2.16	1.793333333	0.02	III	Mild/Moderate
FR23669829	Female	Shallow	0.806451613	3.089285714	2.494047619	0.339285714	IV	Severe
FR23669840	Male	Deep	0.285714286	3.080246914	2.956790123	0.432098765	IV	Severe
FR23669842	Female	Shallow	0.465408805	2.530864198	2.074074074	0.179012346	II	Mild/Moderate
FR23670375	Male	Shallow	0.59	2.9375	2.527777778	0.888888889	III	Mild/Moderate
FR23670424	Male	Deep	1.072164948	3.359649123	2.447368421	0.526315789	III	Severe
FR23670426	Female	Deep	0.472222222	2.839506173	2.419753086	0.43452381	III	Mild/Moderate
FR23670427	Female	Deep	0.119047619	1.832335329	1.71257485	0.148809524	III	Mild/Moderate
FR23670428	Female	Shallow	0.240740741	3.017857143	2.785714286	0.398809524	III	Mild/Moderate
FR23670429	Male	Deep	0.669230769	3.096153846	2.538461538	0.326923077	III	Mild/Moderate
FR23670430	Female	Shallow	0.161764706	2.586666667	2.44	0.38	IV	Severe
FR23670431	Female	Deep	1.458015267	3.530120482	2.379518072	0.202380952	IV	Severe
FR23670432	Male	Shallow	0.340136054	2.83974359	2.519230769	0.320512821	III	Severe
FR23670433	Female	Shallow	0.585106383	3.115384615	2.762820513	0.448717949	III	Mild/Moderate
FR23670434	Male	Shallow	0.119047619	1.832335329	1.71257485	0.148809524	III	Mild/Moderate
FR23670435	Female	Deep	0.611510791	3.342105263	2.782894737	0.461538462	II	Mild/Moderate
FR23670436	Female	Shallow	1.143835616	3.709677419	2.632258065	0.352564103	II	Mild/Moderate
FR23670437	Male	Shallow	2.265306122	3.552083333	2.452380952	0.103174603	IV	Severe
FR23670438	Male	Deep	0.769736842	3.890322581	3.135483871	0.83974359	IV	Severe
FR23670440	Male	Shallow	0.22556391	3.006410256	2.814102564	0.448717949	III	Mild/Moderate
FR23670441	Female	Shallow	0.403508772	2.553333333	2.246666667	0.48	III	Mild/Moderate
FR23670442	Female	Deep	0.291338583	2.855072464	2.586956522	0.430555556	III	Mild/Moderate
FR23670443	Female	Shallow	0.198717949	2.519230769	2.320512821	0.474358974	IV	Severe
FR23670444	Female	Deep	0.482758621	2.646153846	2.223076923	0.462121212	IV	Severe
FR23670448	Male	Deep	0.240740741	3.017857143	2.785714286	0.398809524	III	Mild/Moderate
FR23670449	Female	Deep	0.423076923	2.320987654	2.049382716	0.061728395	II	Mild/Moderate
FR23670450	Female	Shallow	0.226415094	1.836477987	1.622641509	0.067901235	II	Mild/Moderate
FR23670453	Male	Shallow	0.641025641	2.621794872	2.141025641	0.057692308	III	Mild/Moderate
FR23670454	Female	Deep	0.9	2.476190476	1.833333333	0.05952381	III	Severe
FR23670456	Male	Shallow	0.19047619	3.125	2.958333333	0.283333333	III	Mild/Moderate
FR23670457	Female	Shallow	2.265306122	3.552083333	2.452380952	0.103174603	III	Severe
FR23670458	Male	Shallow	0.235294118	2.567901235	2.345679012	0.191358025	III	Mild/Moderate
FR23670459	Female	Deep	0.378378378	2.952380952	2.619047619	0.523809524	III	Severe
FR23670460	Female	Shallow	0.428571429	2.333333333	2.193333333	0.226666667	III	Severe
FR23670462	Male	Deep	1.018867925	2.833333333	2.383333333	0.258333333	III	Mild/Moderate

FR23670463	Male	Shallow	1.52173913	2.439393939	2.174242424	0.196969697	III	Mild/Moderate
FR23670464	Female	Shallow	1.338235294	3.25	2.708333333	0.160714286	III	Mild/Moderate
FR23670465	Female	Deep	0.482758621	2.646153846	2.223076923	0.462121212	III	Severe
FR23670466	Male	Deep	2.206896552	2.891666667	2.348484848	0.181818182	III	Mild/Moderate
FR23670467	Female	Deep	0.905660377	3.158730159	2.396825397	0.341269841	III	Mild/Moderate
FR23670468	Female	Shallow	0.777777778	3.023809524	2.56547619	0.630952381	II	Mild/Moderate
FR23670469	Female	Shallow	0.636363636	3.067114094	2.620481928	0.505952381	IV	Severe
FR23670470	Female	Deep	0.636363636	3.067114094	2.620481928	0.505952381	IV	Severe
FR23670472	Female	Deep	1.338235294	3.25	2.708333333	0.160714286	III	Mild/Moderate
FR23670474	Male	Deep	0.686956522	2.802469136	2.314814815	0.345679012	IV	Severe
FR23670475	Male	Shallow	0.686956522	2.802469136	2.314814815	0.345679012	IV	Severe
FR23670476	Female	Shallow	0.304964539	2.237179487	1.961538462	0.352564103	III	Severe
FR23670477	Female	Deep	1.43902439	3.08	1.9	0.146666667	III	Severe
FR23670478	Male	Deep	0.777777778	3.038461538	2.275641026	0.532051282	IV	Mild/Moderate
FR23670479	Male	Shallow	0.248120301	2.283950617	2.080246914	0.228395062	IV	Mild/Moderate
FR23670480	Male	Deep	1.642335766	3.626666667	2.126666667	0.233333333	IV	Severe
FR23670481	Male	Shallow	1.453781513	3.005952381	1.976190476	0.494047619	IV	Severe
FR23670482	Male	Deep	0.991666667	3.019230769	2.256410256	0.301282051	III	Mild/Moderate
FR23670483	Male	Shallow	0.5625	2.631944444	2.444444444	0.5	III	Mild/Moderate
FR23670484	Male	Shallow	0.991666667	3.019230769	2.256410256	0.301282051	II	Mild/Moderate
FR23670485	Male	Shallow	0.903030303	3.255952381	2.369047619	0.31547619	III	Mild/Moderate
FR23670486	Male	Shallow	0.903030303	3.255952381	2.369047619	0.31547619	II	Mild/Moderate
FR23670488	Male	Deep	1.380952381	3.486666667	2.133333333	0.12	III	Mild/Moderate
FR23670490	Female	Shallow	2.286666667	4.882716049	2.765432099	0.481481481	IV	Severe
FR23670491	Female	Deep	2.286666667	4.882716049	2.765432099	0.481481481	IV	Severe
FR23670493	Male	Shallow	1.538461538	3.308641975	2.320987654	0.296296296	III	Severe
FR23670494	Male	Shallow	0.678832117	2.583333333	1.9375	0.229166667	III	Mild/Moderate
FR23670495	Male	Deep	2.530612245	4.142857143	1.928571429	0.160714286	III	Mild/Moderate
FR23670496	Male	Deep	0.669491525	2.769230769	2.262820513	0.506410256	III	Severe
FR23670497	Female	Shallow	1.433962264	2.953333333	2.446666667	0.553333333	III	Mild/Moderate
FR23670498	Male	Shallow	0.178082192	2.761904762	2.607142857	0.291666667	III	Mild/Moderate
FR23670499	Male	Shallow	2.530612245	4.142857143	1.928571429	0.160714286	III	Severe
FR23670504	Female	Deep	1.066115702	2.993589744	2.166666667	0.044871795	IV	Severe
FR23670505	Female	Shallow	0.669491525	2.769230769	2.262820513	0.506410256	III	Mild/Moderate
FR23670506	Male	Deep	0.744	3.047619048	2.494047619	0.505952381	IV	Severe
FR23670509	Male	Shallow	1.870967742	4.447368421	3.446969697	0.515151515	II	Mild/Moderate
FR23670512	Female	Shallow	1.066115702	2.993589744	2.166666667	0.044871795	IV	Severe
FR23670514	Male	Shallow	1.824	3.75	2.288461538	0.320512821	IV	Severe

FR23670636	Male	Shallow	0.824675325	3.297619048	2.541666667	0.458333333	II	No periodontitis
FR23670637	Male	Shallow	NA	NA	NA	NA	IV	Severe
FR23670663	Male	Deep	0.442176871	2.5	2.083333333	0.275641026	IV	Severe
FR23670666	Male	Shallow	0.555555556	2.630952381	2.125	0.261904762	IV	Severe
FR23670690	Male	Shallow	0.286666667	3.125	2.869047619	0.458333333	III	Mild/Moderate
FR23670708	Male	Deep	0.05982906	2.567901235	2.524691358	0.722222222	IV	Severe
FR25324404	Male	Shallow	0.58	2.722222222	2.185185185	0.462962963	III	Severe
FR25324407	Female	Shallow	0.94214876	3.253623188	2.427536232	0.231884058	III	Mild/Moderate
FR25324409	Female	Deep	1.266666667	3.882352941	3.722689076	0.475	IV	Severe
FR25324410	Female	Shallow	0.217391304	1.976190476	1.797619048	0.041666667	III	Mild/Moderate
FR25324413	Female	Shallow	1.463087248	3.482142857	2.18452381	0.285714286	III	Mild/Moderate
FR25324415	Male	Shallow	0.661417323	2.660714286	2.160714286	0.345238095	IV	Severe
FR25324416	Male	Deep	0	2.458333333	2.633333333	0.95	III	Severe
FR25324423	Male	Shallow	0.04	2.708333333	2.672619048	0.470238095	III	Mild/Moderate
FR25324424	Female	Shallow	0.102362205	1.826388889	1.736111111	0.020833333	IV	Severe
FR25324433	Male	Shallow	0.042424242	1.946428571	1.904761905	0.077380952	III	Severe
FR25324436	Male	Deep	0.307692308	2.769230769	2.666666667	0.480769231	III	Severe
FR25324438	Female	Shallow	0.957894737	2.847328244	2.152671756	0.28030303	III	Mild/Moderate
FR25324440	Female	Deep	0.24691358	2.071428571	1.833333333	0.023809524	III	Mild/Moderate
FR25324441	Female	Deep	0.477124183	2.696428571	2.261904762	0.505952381	III	Mild/Moderate
FR25324447	Female	Deep	0.72	2.486666667	1.766666667	0.04	III	Mild/Moderate
FR25324450	Female	Deep	0	2.894736842	2.894736842	0.894736842	IV	Severe
FR25324457	Female	Deep	0.205673759	2	1.827380952	0.05952381	III	Mild/Moderate
FR25324460	Male	Shallow	0.308270677	3.012345679	2.759259259	0.518518519	III	Mild/Moderate
FR25324464	Male	Deep	0.458333333	2.970238095	2.511904762	0.214285714	IV	Severe
FR25324466	Male	Deep	0.869863014	3.413333333	2.566666667	0.426666667	IV	Severe
FR25324470	Female	Shallow	0.581818182	2.625	2.053571429	0.232142857	III	Severe
FR25324480	Male	Deep	0.284722222	3.358974359	3.096153846	0.224358974	III	Mild/Moderate
FR25324481	Female	Deep	0.904761905	2.833333333	2.113636364	0.106060606	III	Mild/Moderate
FR25324490	Female	Deep	1.918367347	3.079365079	2.333333333	0.19047619	IV	Severe
FR25324492	Female	Shallow	1.12	3.291666667	2.458333333	0.583333333	III	Mild/Moderate
FR25324493	Female	Shallow	2.368421053	3.141666667	2.016666667	0.625	III	Mild/Moderate
FR25324498	Male	Shallow	2.195121951	3.477272727	2.795454545	0.628787879	II	No periodontitis
FR25324504	Female	Deep	0.58041958	3.607142857	3.113095238	0.928571429	III	Severe
FR25324506	Male	Shallow	1.574074074	3.440366972	2.660550459	0.434782609	I	No periodontitis
FR25324522	Male	Shallow	2.2	2.788461538	2.435897436	0.673076923	III	Mild/Moderate
FR25324530	Female	Shallow	0.408759124	3.24691358	2.901234568	0.487654321	III	Mild/Moderate
FR25324534	Male	Shallow	0.171875	2.043209877	1.907407407	0.055555556	III	Mild/Moderate

FR25324545	Male	Shallow	0.134328358	2.125	2.0625	0.048611111	IV	Severe
FR25324554	Male	Shallow	0.8	3	2.333333333	0.30952381	III	Mild/Moderate
FR25324556	Male	Shallow	0.722627737	3.023809524	2.43452381	0.654761905	I	No periodontitis
FR25324563	Female	Shallow	0.75	2.621212121	2.234848485	0.606060606	III	Mild/Moderate
FR25324568	Female	Shallow	0.536585366	2.965277778	2.506944444	0.375	III	Mild/Moderate
FR25324571	Female	Shallow	1.5	2.601449275	2.471014493	0.615942029	III	Mild/Moderate
FR25324575	Male	Deep	0.153846154	2.875	2.732142857	0.68452381	III	Mild/Moderate
FR25324576	Female	Shallow	0.725609756	2.910714286	2.202380952	0.488095238	III	Mild/Moderate
FR25324582	Male	Shallow	0.590551181	3.154761905	2.708333333	0.80952381	IV	Severe
FR25324591	Female	Shallow	0.450704225	3.465277778	3.020833333	0.673611111	III	Mild/Moderate
FR25324592	Female	Shallow	0.008333333	1.976190476	1.970238095	0.041666667	III	Mild/Moderate
FR25324593	Male	Shallow	0.5	3.438271605	2.938271605	0.5	III	Severe
FR25324594	Male	Shallow	0.128787879	3.034722222	2.916666667	0.466666667	III	Mild/Moderate
FR25324595	Male	Deep	0.572463768	2.119047619	1.648809524	0	III	Severe
FR25324596	Female	Deep	0.292134831	2.739130435	2.550724638	0.528985507	III	Severe
FR25324598	Male	Shallow	0.652173913	3.254385965	2.859649123	0.728070175	IV	Severe
FR25324601	Female	Shallow	0.484536082	2.462962963	2.172839506	0.419753086	II	Mild/Moderate
FR25324606	Male	Deep	1.607142857	3.818181818	2.805555556	0.652777778	III	Severe
FR25324610	Female	Deep	0.1	1.737179487	1.66025641	0.019230769	III	Mild/Moderate
FR25324612	Female	Shallow	0.1	2.033333333	1.946666667	0.026666667	III	Mild/Moderate
FR25324619	Female	Deep	0.179487179	3.013888889	2.868055556	0.673611111	III	Mild/Moderate
FR25324622	Male	Deep	1.918367347	3.079365079	2.333333333	0.19047619	III	Mild/Moderate
FR25324624	Female	Shallow	0.625	2.275362319	1.949275362	0.036231884	III	Mild/Moderate
FR25324634	Female	Shallow	0.756578947	2.607142857	1.922619048	0.125	III	Mild/Moderate
FR25324638	Male	Shallow	0.474358974	2.666666667	2.226190476	0.488095238	III	Mild/Moderate
FR25324639	Male	Deep	0.181818182	2.398809524	2.327380952	0.05952381	IV	Severe
FR25324641	Male	Deep	0.666666667	3.111111111	2.641975309	0.765432099	III	Severe
FR25324642	Male	Deep	0.265957447	2.313333333	2.146666667	0.233333333	IV	Severe
FR25324646	Female	Deep	0.875	2.595238095	2.136904762	0.345238095	III	Mild/Moderate
FR25324648	Male	Deep	0	2.185185185	2.185185185	0.115384615	IV	Severe
FR25324649	Male	Deep	0.555555556	2.565217391	2.239130435	0.398550725	IV	Severe
FR25324650	Female	Shallow	0.754966887	2.363095238	1.68452381	0.035714286	III	Mild/Moderate
FR25324652	Male	Shallow	0.25	1.952380952	1.755952381	0.011904762	II	Mild/Moderate
FR25324658	Male	Shallow	1.940677966	3.713333333	2.186666667	0.2	II	Mild/Moderate
FR25324660	Female	Shallow	1.456140351	2.549382716	1.524691358	0.12345679	IV	Severe
FR25324661	Male	Shallow	0.45625	3.351190476	2.916666667	0.232142857	IV	Severe
FR25324666	Female	Deep	0.798245614	2.727272727	2.090909091	0.277777778	III	Mild/Moderate
FR25324667	Female	Shallow	0.444444444	2.976190476	2.666666667	0.44047619	III	Mild/Moderate

FR25324671	Female	Shallow	0.344537815	2.971014493	2.673913043	0.565217391	III	Mild/Moderate
FR25324679	Female	Deep	1.08411215	2.673076923	1.929487179	0.185897436	III	Mild/Moderate
FR25324683	Female	Deep	2.368421053	3.141666667	2.016666667	0.625	IV	Severe
FR25324688	Female	Shallow	0.6	3.564102564	3.083333333	0.570512821	III	Severe
FR25324808	Female	Deep	0.793333333	3.255952381	2.547619048	0.44047619	III	Severe
FR25324817	Male	Shallow	0.704918033	3.409395973	2.832214765	0.38	III	Mild/Moderate
FR25324839	Male	Shallow	0.5	2.441558442	2.136363636	0.365384615	IV	Severe
FR25324850	Male	Shallow	0.368421053	1.797619048	1.547619048	0.035714286	IV	Severe
FR25324856	Female	Deep	0.482758621	2.340277778	2.048611111	0.201388889	III	Mild/Moderate
FR25324858	Female	Shallow	0.222222222	1.948717949	1.769230769	0.025641026	III	Severe
FR25324865	Male	Deep	0.128787879	3.034722222	2.916666667	0.466666667	III	Mild/Moderate
FR25324872	Female	Shallow	2.061728395	3.484848485	2.21969697	0.272727273	IV	Severe
FR25324881	Female	Shallow	0.185185185	2.544871795	2.384615385	0.493589744	III	Mild/Moderate
FR25324882	Male	Deep	0.926229508	2.91025641	2.185897436	0.474358974	IV	Severe
FR25324883	Female	Shallow	1.756097561	3.72	2.333333333	0.41025641	III	Severe
FR25324884	Female	Shallow	0.4453125	3.266666667	2.886666667	0.473333333	III	Mild/Moderate
FR25324886	Male	Deep	0.316666667	3.571428571	3.345238095	0.922619048	III	Severe
FR25324887	Female	Shallow	0.106382979	3.470238095	3.410714286	0.541666667	III	Mild/Moderate
FR25324888	Male	Shallow	0.287671233	2.169811321	1.943396226	0.12345679	III	Severe
FR25324889	Female	Deep	0.336363636	3.104166667	2.847222222	0.513888889	IV	Severe
FR25324890	Male	Shallow	0.050955414	1.851190476	1.803571429	0.023809524	IV	Severe
FR25324891	Male	Shallow	0.308270677	3.012345679	2.759259259	0.518518519	III	Severe
FR25324893	Female	Deep	0.666666667	3.111111111	2.641975309	0.765432099	IV	Severe
FR25324894	Female	Deep	0.45625	3.351190476	2.916666667	0.232142857	III	Mild/Moderate
FR25324895	Male	Deep	0.622222222	2.993055556	2.409722222	0.555555556	III	Mild/Moderate
FR25324897	Male	Deep	0.319444444	2.75	2.430555556	0.125	III	Mild/Moderate
FR25324899	Male	Shallow	0.75	2.621212121	2.234848485	0.606060606	III	Mild/Moderate
FR25324900	Female	Shallow	0.869863014	3.413333333	2.566666667	0.426666667	III	Mild/Moderate
FR25324902	Female	Deep	0.46969697	3.444444444	3.013888889	0.513888889	IV	Severe
FR25324903	Female	Shallow	0.104938272	2.726190476	2.625	0.553571429	III	Mild/Moderate
FR25324904	Male	Deep	1.044871795	2.964285714	1.994047619	0.261904762	III	Mild/Moderate
FR25324905	Female	Deep	0.068965517	2.00617284	1.944444444	0.049382716	III	Mild/Moderate
FR25324906	Male	Shallow	NA	NA	NA	NA	III	Mild/Moderate
FR25324907	Female	Shallow	0.299145299	2.611111111	2.368055556	0.576388889	III	Mild/Moderate
FR25324908	Male	Shallow	0.283687943	2.738095238	2.5	0.571428571	III	Mild/Moderate
FR25324909	Male	Shallow	1.438596491	2.913043478	1.724637681	0.02173913	I	No periodontitis
FR25324910	Male	Deep	0.154320988	1.803571429	1.654761905	0.017857143	III	Mild/Moderate
FR25324911	Female	Shallow	0.830065359	2.820987654	2.037037037	0.135802469	III	Mild/Moderate

FR25324912	Male	Shallow	0.192771084	3.213333333	3.106666667	0.66	III	Mild/Moderate
FR25324913	Male	Shallow	0.319444444	2.75	2.430555556	0.125	III	Mild/Moderate
FR25324914	Male	Shallow	0.111111111	2.08974359	1.987179487	0.083333333	III	Mild/Moderate
FR25324915	Female	Shallow	0.4453125	3.266666667	2.886666667	0.473333333	III	Mild/Moderate
FR25324917	Male	Deep	0.1875	2.222222222	2.079365079	0.03968254	III	Mild/Moderate
FR25324919	Male	Deep	0.709090909	3.19047619	2.494047619	0.416666667	III	Severe
FR25324920	Male	Shallow	1.145833333	4.111111111	3.092592593	0.308641975	III	Mild/Moderate
FR25324921	Male	Shallow	1.12	3.291666667	2.458333333	0.583333333	IV	Severe
FR25324922	Male	Shallow	0.516556291	3.576923077	3.076923077	0.442307692	III	Severe
FR25324923	Male	Shallow	0.6	3.564102564	3.083333333	0.570512821	III	Mild/Moderate
FR25324924	Female	Shallow	1.459677419	3.847826087	2.536231884	0.398550725	III	Mild/Moderate
FR25324925	Male	Shallow	0.520661157	3.333333333	2.876811594	0.695652174	III	Mild/Moderate
FR25324929	Male	Shallow	0.413793103	2.757763975	2.385093168	0.290123457	III	Mild/Moderate
FR25324931	Female	Deep	0.081632653	2.487179487	2.41025641	0.269230769	III	Mild/Moderate
FR25324932	Female	Shallow	0.704918033	3.409395973	2.832214765	0.38	III	Mild/Moderate
FR25324933	Female	Deep	0.659574468	3.58	3.166666667	0.646666667	III	Severe
FR25324934	Male	Shallow	1.266666667	3.882352941	3.722689076	0.475	III	Severe
FR25324935	Female	Shallow	0.659574468	3.58	3.166666667	0.646666667	III	Severe
FR25324936	Male	Deep	0.793333333	3.255952381	2.547619048	0.44047619	III	Mild/Moderate
FR25324937	Female	Deep	0.251968504	1.986666667	1.773333333	0.046666667	III	Mild/Moderate
FR25324938	Female	Deep	0.798245614	2.727272727	2.090909091	0.277777778	IV	Severe
FR25324939	Male	Shallow	0.345679012	2.482142857	2.148809524	0.351190476	IV	Severe
FR25324940	Female	Shallow	1.940677966	3.713333333	2.186666667	0.2	IV	Severe
FR25324942	Female	Shallow	0.131944444	2.011904762	1.898809524	0.029761905	III	Mild/Moderate
FR25324943	Female	Deep	0.030864198	2.553571429	2.523809524	0.648809524	III	Mild/Moderate
FR25324944	Female	Shallow	1.521367521	3.507575758	2.159090909	0.772727273	III	Mild/Moderate
FR25324945	Male	Deep	0.253164557	2.035714286	1.797619048	0.053571429	IV	Severe
FR25324946	Male	Shallow	0.303030303	2.005952381	1.708333333	0.023809524	III	Mild/Moderate
FR25324948	Male	Deep	0.721311475	3.546296296	3.138888889	0.861111111	III	Severe
FR25324950	Male	Deep	1.066666667	3.119047619	2.166666667	0.589285714	IV	Severe
FR25324951	Male	Shallow	0.175438596	2.820987654	2.697530864	0.567901235	IV	Severe
FR25324952	Male	Shallow	2.8375	4.885964912	2.894736842	0.403508772	III	Mild/Moderate
FR25324953	Female	Shallow	0.236220472	1.987179487	1.794871795	0.083333333	III	Mild/Moderate
FR25324956	Female	Shallow	1.547619048	4.118055556	2.763888889	0.361111111	II	Mild/Moderate
FR25324957	Female	Shallow	0.8671875	3.229166667	2.458333333	0.527777778	IV	Mild/Moderate
FR25324958	Male	Shallow	0.441666667	2.553333333	2.2	0.513333333	IV	Severe
FR25324959	Male	Deep	0.373626374	3	2.762237762	0.784722222	IV	Severe
FR25324960	Female	Shallow	0.801587302	3.273333333	2.6	0.68	II	No periodontitis

FR25324961	Female	Shallow	1.586206897	4.773333333	3.24	0.526666667	III	Mild/Moderate
FR25324962	Female	Shallow	2.1953125	4.363636364	2.234848485	0.204545455	I	No periodontitis
FR25324963	Female	Shallow	0.216216216	3.373333333	3.16	0.426666667	II	No periodontitis
FR25324964	Male	Shallow	0.297520661	3.097222222	2.847222222	0.673611111	II	Mild/Moderate
FR25324966	Female	Shallow	0.428571429	2.932098765	2.74691358	0.660493827	III	Mild/Moderate
FR25324967	Female	Shallow	0.802816901	2.916666667	2.352380952	0.648148148	I	No periodontitis
FR25324968	Female	Shallow	0.625899281	4.133333333	3.553333333	0.426666667	III	Mild/Moderate
FR25324969	Female	Shallow	0.66	3.906666667	3.246666667	0.586666667	II	Mild/Moderate
FR25324970	Male	Shallow	0.676056338	2.923611111	2.256944444	0.173611111	II	Mild/Moderate
FR25324971	Female	Shallow	0.390728477	2.641975309	2.277777778	0.283950617	II	Mild/Moderate
FR25324972	Male	Shallow	0.173913043	3.029761905	2.863095238	0.845238095	III	Mild/Moderate
FR25324973	Female	Shallow	1.348684211	3.529411765	2.189542484	0.493589744	III	Mild/Moderate
FR25324974	Female	Shallow	2.1953125	4.363636364	2.234848485	0.204545455	II	Mild/Moderate
FR25324976	Male	Shallow	0.488636364	2.65	2.338582677	0.765151515	III	Mild/Moderate
FR25324981	Female	Shallow	0.520661157	3.333333333	2.876811594	0.695652174	I	No periodontitis
FR25324982	Female	Shallow	0.154362416	3.032051282	2.884615385	0.641025641	III	Severe
FR25324983	Male	Deep	0.265151515	2.756944444	2.513888889	0.263888889	II	Mild/Moderate
FR25324986	Male	Shallow	0.555555556	2.565217391	2.239130435	0.398550725	III	Severe
FR25324989	Female	Deep	1.161904762	3.173913043	2.289855072	0.144927536	IV	Severe
FR25324995	Female	Deep	0.43902439	3.653846154	3.307692308	0.58974359	III	Mild/Moderate
FR25324996	Female	Shallow	0.344537815	2.971014493	2.673913043	0.565217391	III	Mild/Moderate
FR25324997	Male	Deep	0.75	2.938271605	2.49382716	0.604938272	III	Severe
FR25325000	Male	Deep	0.208955224	2.535714286	2.369047619	0.351190476	III	Mild/Moderate
FR25325001	Male	Shallow	0.267716535	2.196969697	1.939393939	0.03030303	III	Mild/Moderate
FR25325003	Female	Deep	0.770992366	2.767857143	2.166666667	0.363095238	III	Mild/Moderate
FR25325004	Male	Deep	0.324561404	2.708333333	2.451388889	0.694444444	III	Severe
FR25325009	Male	Deep	0.041666667	1.895833333	1.868055556	0.041666667	IV	Severe
FR25325011	Male	Deep	0	2.066666667	2.066666667	0.033333333	III	Mild/Moderate
FR25325012	Male	Shallow	0.243421053	3.308176101	3.094339623	0.407407407	II	No periodontitis
FR25325014	Male	Deep	1.650943396	3.2	2.033333333	0.32	IV	Severe
FR25325021	Female	Shallow	0.801587302	3.273333333	2.6	0.68	III	Mild/Moderate
FR25325026	Female	Shallow	0.503649635	2.948717949	2.506410256	0.467948718	III	Mild/Moderate
FR25325027	Female	Shallow	1.270967742	2.950617284	1.734567901	0.191358025	II	No periodontitis
FR25325028	Male	Deep	0.925	2.717391304	2.18115942	0.47826087	IV	Severe
FR25325030	Male	Shallow	1.139130435	2.820512821	1.980769231	0.166666667	III	Mild/Moderate
FR25325032	Female	Deep	0.46969697	3.444444444	3.013888889	0.513888889	III	Mild/Moderate
FR25325035	Male	Shallow	NA	NA	NA	NA	IV	Mild/Moderate
FR25325036	Male	Shallow	0	3.821428571	3.833333333	0.677777778	III	Mild/Moderate

FR25325038	Female	Deep	0.408759124	3.24691358	2.901234568	0.487654321	III	Mild/Moderate
FR25325040	Female	Shallow	0.674242424	2.866666667	2.407407407	0.567901235	II	No periodontitis
FR25325041	Male	Shallow	0.083333333	1.833333333	1.794871795	0.070512821	II	Mild/Moderate
FR25325042	Female	Shallow	1.124137931	3.62	2.533333333	0.566666667	II	No periodontitis
FR25325044	Female	Shallow	0.651376147	2.773809524	2.351190476	0.511904762	III	Mild/Moderate
FR25325046	Male	Shallow	1	3.409090909	2.742424242	0.772727273	III	Mild/Moderate
FR25325048	Female	Shallow	0.214285714	3.136904762	2.958333333	0.613095238	IV	Severe
FR25325053	Male	Deep	0.235294118	2.791666667	2.625	0.642857143	IV	Severe
FR25325056	Female	Shallow	1.756097561	3.72	2.333333333	0.41025641	IV	Severe
FR25325060	Male	Shallow	0.080882353	2.476190476	2.410714286	0.642857143	IV	Severe
FR25325062	Male	Deep	0.43902439	3.653846154	3.307692308	0.58974359	III	Mild/Moderate
FR25325069	Female	Deep	NA	NA	NA	NA	IV	Severe
FR25325070	Female	Shallow	0.233333333	2.401234568	2.228395062	0.388888889	II	Mild/Moderate
FR25325073	Female	Deep	0.84	3.567901235	2.919753086	0.50617284	III	Mild/Moderate
FR25325083	Female	Deep	0.94214876	3.253623188	2.427536232	0.231884058	III	Severe
FR25325094	Female	Deep	1.421875	2.856060606	2.166666667	0.590909091	IV	Severe
FR25325096	Female	Deep	0.444444444	2.976190476	2.666666667	0.44047619	III	Mild/Moderate
FR25325105	Male	Shallow	0.935779817	3.083333333	2.304347826	0.355072464	III	Mild/Moderate
FR25325113	Male	Shallow	0.453488372	2.404761905	2.172619048	0.339285714	II	No periodontitis
FR25325150	Male	Deep	0.262411348	2.845679012	2.617283951	0.283950617	IV	Severe
FR25325160	Female	Shallow	0.586538462	2.958333333	2.534722222	0.520833333	IV	Severe
FR25325170	Male	Shallow	0.583333333	3.464285714	2.880952381	0.43452381	IV	Severe
FR25325171	Female	Shallow	2.2	2.788461538	2.435897436	0.673076923	IV	Severe
FR25325173	Male	Shallow	0.802816901	2.916666667	2.352380952	0.648148148	III	Mild/Moderate
FR25325178	Male	Deep	0.171641791	2.94047619	2.803571429	0.630952381	IV	Severe
FR25325179	Male	Shallow	1.905660377	2.966666667	2.125	0.216666667	III	Mild/Moderate
FR25325180	Male	Deep	0.235294118	2.791666667	2.625	0.642857143	IV	Severe
FR25325181	Female	Shallow	0.263888889	3.185897436	2.942307692	0.641025641	III	Mild/Moderate
FR25325182	Female	Deep	0.158730159	2.648148148	2.524691358	0.604938272	IV	Severe
FR25325189	Female	Deep	2.114285714	3.201388889	2.211538462	0.397435897	III	Severe
FR25325190	Female	Shallow	0.428571429	2.932098765	2.74691358	0.660493827	III	Mild/Moderate
FR25325191	Female	Deep	1.318471338	3.351851852	2.074074074	0.179012346	III	Mild/Moderate
FR25325192	Female	Shallow	0.398305085	2.916666667	2.590277778	0.326388889	I	No periodontitis
FR25325194	Male	Shallow	1.225806452	3.220238095	2.31547619	0.636904762	III	Mild/Moderate
FR25325197	Female	Deep	1	3.409090909	2.742424242	0.772727273	IV	Severe
FR25325199	Female	Shallow	0.26	2.796296296	2.555555556	0.586419753	III	Mild/Moderate
FR25325200	Male	Deep	0.086092715	2.018518519	1.938271605	0.00617284	III	Mild/Moderate
FR25325207	Female	Shallow	1.574074074	3.440366972	2.660550459	0.434782609	IV	Severe

FR25325218	Female	Deep	0.826530612	2.827160494	2.327160494	0.549382716	III	Mild/Moderate
FR25325234	Female	Deep	2.136986301	3.126666667	2.086666667	0.04	III	Mild/Moderate
FR25325237	Female	Deep	0.406666667	3.205128205	2.802469136	0.333333333	III	Mild/Moderate
FR25325239	Male	Deep	0.205607477	3.138888889	2.986111111	0.479166667	IV	Severe
FR25325242	Male	Shallow	0.1875	2.782051282	2.647435897	0.391025641	III	Mild/Moderate
FR25325243	Female	Shallow	1.042553191	3.154320988	2.24691358	0.259259259	III	Mild/Moderate
FR25325250	Male	Shallow	0.984375	2.291666667	1.916666667	0.226190476	III	Severe
FR25325251	Male	Shallow	0.557377049	2.456790123	2.037037037	0.530864198	III	Mild/Moderate
FR25325252	Female	Deep	0.616666667	2.422619048	2.202380952	0.404761905	III	Mild/Moderate
FR25325257	Male	Shallow	0.04	2.708333333	2.672619048	0.470238095	II	Mild/Moderate
FR25325260	Female	Shallow	0.780701754	3.320512821	2.75	0.429487179	III	Severe
FR25325263	Female	Deep	0.352	2.743589744	2.461538462	0.269230769	IV	Severe
FR25325268	Male	Shallow	0.358024691	3.401234568	3.043209877	0.50617284	III	Mild/Moderate
FR25325269	Male	Deep	1.444444444	3.095238095	1.96031746	0.134920635	III	Mild/Moderate
FR25325271	Female	Shallow	0.297520661	3.097222222	2.847222222	0.673611111	III	Mild/Moderate
FR25325272	Female	Deep	0.509677419	2.976190476	2.505952381	0.220238095	III	Mild/Moderate
FR25325273	Male	Shallow	0.562043796	2.678571429	2.220238095	0.130952381	III	Mild/Moderate
FR25325274	Male	Deep	0.718309859	3.06	2.72	0.826666667	III	Mild/Moderate
FR25325275	Female	Deep	0.737288136	2.987654321	2.450617284	0.450617284	IV	Severe
FR25325276	Female	Deep	0.821782178	3.916666667	3.287878788	0.431818182	IV	Severe
FR25325279	Male	Shallow	0.968085106	3.080246914	2.518518519	0.172839506	III	Mild/Moderate
FR25325280	Male	Shallow	0.216216216	3.373333333	3.16	0.426666667	IV	Severe
FR25325281	Male	Shallow	0.317365269	2.964071856	2.646706587	0.31547619	III	Mild/Moderate
FR25325282	Male	Shallow	0.441666667	2.553333333	2.2	0.513333333	III	Mild/Moderate
FR25325283	Male	Shallow	1.402777778	3.193333333	2.52	0.306666667	III	Mild/Moderate
FR25325284	Female	Shallow	0.755102041	3.240740741	2.783950617	0.401234568	III	Mild/Moderate
FR25325285	Male	Shallow	1.634615385	3.733333333	2.788888889	0.705882353	III	Mild/Moderate
FR25325286	Female	Shallow	1.634615385	3.733333333	2.788888889	0.705882353	II	No periodontitis
FR25325287	Female	Shallow	1.217391304	2.827380952	2.327380952	0.380952381	II	No periodontitis
FR25325288	Male	Deep	0.013986014	2.988095238	2.976190476	0.613095238	IV	Severe
FR25325289	Female	Shallow	0.484536082	2.462962963	2.172839506	0.419753086	I	No periodontitis
FR25325290	Male	Shallow	0.917808219	2.972222222	2.351851852	0.166666667	II	Mild/Moderate
FR25325293	Female	Shallow	0.795454545	3.166666667	2.493589744	0.544871795	IV	Severe
FR25325294	Male	Shallow	0.064516129	2.785714286	2.738095238	0.369047619	III	Mild/Moderate
FR25325295	Female	Deep	0.795454545	3.166666667	2.493589744	0.544871795	IV	Severe
FR25325296	Female	Shallow	1.402777778	3.193333333	2.52	0.306666667	III	Mild/Moderate
FR25325297	Female	Deep	0.674242424	2.866666667	2.407407407	0.567901235	III	Mild/Moderate
FR25325298	Female	Shallow	0.214285714	2.685897436	2.570512821	0.788461538	III	Mild/Moderate

FR25325300	Female	Shallow	0.134751773	2.470238095	2.357142857	0.535714286	III	Mild/Moderate
FR25325302	Female	Shallow	0.263888889	3.185897436	2.942307692	0.641025641	I	No periodontitis
FR25325303	Female	Shallow	0.722627737	3.023809524	2.43452381	0.654761905	III	Mild/Moderate
FR25325304	Female	Deep	0.98125	2.987654321	2.018518519	0.12345679	IV	Severe
FR25325305	Male	Shallow	0.223076923	3.179487179	2.993589744	0.551282051	III	Severe
FR25325306	Female	Shallow	0.057692308	1.910714286	1.857142857	0.05952381	II	Mild/Moderate
FR25325307	Female	Deep	0.292993631	3.136904762	2.863095238	0.523809524	III	Mild/Moderate
FR25325308	Female	Shallow	0.917808219	2.972222222	2.351851852	0.166666667	III	Mild/Moderate
FR25325309	Female	Deep	0.588888889	2.775641026	2.435897436	0.512820513	IV	Mild/Moderate
FR25325311	Female	Shallow	0.527777778	2.472222222	2.206666667	0.393333333	IV	Severe
FR25325312	Female	Shallow	1.061643836	3.049382716	2.092592593	0.172839506	III	Mild/Moderate
FR25325313	Male	Deep	0.453488372	2.404761905	2.172619048	0.339285714	III	Severe
FR25325314	Female	Shallow	0.166666667	2.222222222	2.055555556	0.027777778	II	Mild/Moderate
FR25325315	Female	Shallow	NA	NA	NA	NA	I	No periodontitis
FR25325316	Female	Deep	0.965517241	2.856060606	2.007575758	0.613636364	IV	Severe
FR25325317	Female	Shallow	0.069767442	1.948717949	1.891025641	0.038461538	I	No periodontitis
FR25325318	Female	Shallow	0.033333333	2.275641026	2.243589744	0.070512821	I	No periodontitis
FR25325319	Female	Shallow	NA	NA	NA	NA	IV	Severe
FR25325320	Male	Shallow	0.524271845	2.566666667	2.206666667	0.32	III	Mild/Moderate
FR25325321	Male	Shallow	0.503649635	2.948717949	2.506410256	0.467948718	III	Mild/Moderate
FR25325323	Female	Deep	2.114285714	3.201388889	2.211538462	0.397435897	IV	Mild/Moderate
FR25325324	Female	Deep	0.821782178	3.916666667	3.287878788	0.431818182	III	Mild/Moderate
FR25325325	Female	Shallow	0.583333333	3.464285714	2.880952381	0.43452381	III	Mild/Moderate
FR25325326	Male	Shallow	1.363095238	3.142857143	1.779761905	0.017857143	III	Severe
FR25325327	Male	Deep	0.516556291	3.576923077	3.076923077	0.442307692	III	Severe
FR25325328	Female	Deep	0.733333333	4.073333333	3.34	0.593333333	III	Mild/Moderate
FR25325329	Male	Deep	0.651376147	2.773809524	2.351190476	0.511904762	III	Mild/Moderate
FR25325331	Female	Shallow	0.588888889	2.775641026	2.435897436	0.512820513	II	Mild/Moderate
FR25325332	Female	Shallow	1.124137931	3.62	2.533333333	0.566666667	II	Mild/Moderate
FR25325333	Male	Shallow	0.383838384	3.230769231	2.987179487	0.58974359	III	Mild/Moderate
FR25325334	Male	Deep	0.84	3.567901235	2.919753086	0.50617284	III	Mild/Moderate
FR25325335	Male	Shallow	0.8671875	3.229166667	2.458333333	0.527777778	III	Mild/Moderate
FR25325336	Male	Deep	0.606666667	3.017857143	2.476190476	0.672619048	III	Mild/Moderate
FR25325337	Male	Deep	1.322222222	3.897435897	3.134615385	0.378205128	III	Severe
FR25325338	Female	Shallow	0.585585586	3.6	3.058333333	0.283333333	II	Mild/Moderate
FR25325340	Male	Deep	1.204301075	3.285714286	2.423611111	0.284722222	III	Mild/Moderate
FR25325341	Female	Shallow	0.261261261	3.011904762	2.839285714	0.791666667	II	No periodontitis
FR25325342	Male	Shallow	0.737288136	2.987654321	2.450617284	0.450617284	II	No periodontitis

FR25325343	Male	Deep	0.316666667	3.571428571	3.345238095	0.922619048	IV	Severe
FR25325344	Female	Shallow	1.839506173	4.259259259	2.428571429	0.505952381	III	Mild/Moderate
FR25325345	Male	Shallow	0.197916667	1.825396825	1.674603175	0.079365079	III	Severe
FR25325347	Female	Shallow	0.26	2.796296296	2.555555556	0.586419753	III	Severe
FR25325348	Female	Deep	0.28313253	2.851190476	2.571428571	0.5	III	Severe
FR25325349	Female	Shallow	1.586206897	4.773333333	3.24	0.526666667	III	Mild/Moderate
FR25325350	Male	Shallow	0.622377622	3.319444444	2.701388889	0.784722222	III	Mild/Moderate
FR25325351	Male	Deep	0.622377622	3.319444444	2.701388889	0.784722222	III	Mild/Moderate
FR25325353	Female	Deep	0.070967742	2.648148148	2.580246914	0.475308642	IV	Severe
FR25325355	Female	Shallow	0.070967742	2.648148148	2.580246914	0.475308642	IV	Severe
FR25325356	Female	Shallow	0.430379747	3.095238095	2.69047619	0.505952381	III	Mild/Moderate
FR25325357	Female	Shallow	0.509677419	2.976190476	2.505952381	0.220238095	III	Mild/Moderate
FR25325359	Male	Deep	0.406666667	3.205128205	2.802469136	0.333333333	III	Mild/Moderate
FR25325360	Male	Shallow	0.653225806	3.277777778	2.715277778	0.555555556	III	Mild/Moderate
